# Variable reproducibility in genome-scale public data: A case study using ENCODE ChIP sequencing resource

**DOI:** 10.1016/j.febslet.2015.11.027

**Published:** 2015-12-21

**Authors:** Guillaume Devailly, Anna Mantsoki, Tom Michoel, Anagha Joshi

**Affiliations:** The Roslin Institute, University of Edinburgh, Easter Bush Campus, Midlothian EH25 9RG, United Kingdom

**Keywords:** ENCODE, encyclopaedia of DNA elements, TFBS, transcription factor binding site, ChIP, chromatin immunoprecipitation, ChIP-seq, ChIP followed by massively parallel sequencing, UV, ultraviolet, FAANG, functional annotation of animal genomes, UCSC, University of California, Santa Cruz, TSS, transcriptional start site, FRiP, Fraction of Reads in Peaks, FPKM, Fragments per Kilo base per Million, Encyclopaedia of DNA element, Chromatin immunoprecipitation sequencing, Transcription factor, Data integration

## Abstract

Genome-wide data is accumulating in an unprecedented way in the public domain. Re-mining this data shows great potential to generate novel hypotheses. However this approach is dependent on the quality (technical and biological) of the underlying data. Here we performed a systematic analysis of chromatin immunoprecipitation (ChIP) sequencing data of transcription and epigenetic factors from the encyclopaedia of DNA elements (ENCODE) resource to demonstrate that about one third of conditions with replicates show low concordance between replicate peak lists. This serves as a case study to demonstrate a caveat concerning genome-wide analyses and highlights a need to validate the quality of each sample before performing further associative analyses.

## Highlights

• Analysis of 135 ChIP sequencing experiments from ENCODE belonging to 57 conditions with replicates.
• A third of conditions are discordant between replicates, and another fourth have different sensitivity.
• Data analysis or integration helps determine biological integrity of individual samples.

## 1 Introduction

The ENCODE project has set up a model to generate large data to understand mammalian transcription control by pooling resources across the globe. The easy accessibility of these data, including processed files, has resulted into many researchers world-wide using the ENCODE resource to learn new biology, either by integrating existing data [1] or by integrating with their own new data [2]. An especially valuable part of the ENCODE resource concerns the identification of the binding sites of transcription factors and chromatin modifiers at genome-wide scale using chromatin immuno-precipitation (ChIP) followed by high throughput sequencing (ChIP-seq). This relies on a complex experimental setup involving antibodies, a fragmentation step, chemical or UV cross-link and library preparation from very small amount of DNA [3]. Using these data, regulators have been assigned various roles that are sometimes contradictory [4]. This suggests that despite the high technical quality control of individual samples by the ENCODE consortium [5], serving as a model for many other consortia (BLUEPRINT Epigenetics, Roadmap Epigenomics, FAANG), the post-processing of samples to determine their biological relevance is still incomplete. To investigate this issue, we performed a systematic analysis of the conditions of ChIP sequencing data with replicate experiments in the ENCODE resource.

The ENCODE consortium at UCSC provides 690 ChIP sequencing data sets for transcription factors and transcription regulators, performed using 189 different antibodies in 91 cell lines, sometimes under different cell treatments. It contains 57 conditions where ChIP-seq for the same factor in the same cell line with the same treatment (or absence of treatment) were done multiple times (between 2 and 5, for a total of 135 experiments and corresponding peak lists) and not merged. These experiments were not merged because they differ in one or more of the following: antibody used for the ChIP, laboratory that performed the experiment, or library preparation protocol for the sequencing. It is not mentioned whether these replicates give redundant information, complementary information, or if one of the replicates is of higher quality than the other(s), leaving the end user unarmed to decide which peak list should be used. Notably, these peak lists have also not been merged in other ENCODE data repositories such as encodeproject.org or factorbook.org [6]. Therefore, for each of the 57 conditions with unmerged replicates, we compared individual experiment quality using various approaches and identified 18 conditions where more than 50% of a peak list was irreproducible in the other experiment(s). In these cases, downstream analyses of one peak list or another can lead to different results.

## 2 Materials and methods

ChIP-seq experiments with replicates in the ENCODE TFBS dataset were identified and uniform TFBS peaks files for the corresponding samples were downloaded from the website http://hgdownload.cse.ucsc.edu/goldenPath/hg19/encodeDCC/wgEncodeAwgTfbsUniform/ (Uniform SPP peaks). Common and sample specific peaks were identified using bedtools [7], with a minimal overlap of one base pair. Additionally, for 6 conditions with 2 replicates, alternative peakSeq peak lists were downloaded from http://ftp.ebi.ac.uk/pub/databases/ensembl/encode/integration_data_jan2011/byDataType/peaks/jan2011/peakSeq/optimal/hub/. Bam files for the corresponding experiments were downloaded from https://www.encodeproject.org/, and merged using Samtools [8]. The mean coverages around peaks were computed using Repitools [9]. Statistically significant differentially bound regions were identified using MAnorm [10] with a 150 base pair read shift. Peaks were annotated with the closest TSS using bedtools and GENCODE v21 transcripts annotations [11], after converting the annotation table into hg19 coordinates using liftOver (99.83% conversion success). *De novo* motif analysis was performed using HOMER [12] function findMotifsGenome.pl. Cluster analysis of all ENCODE ChIP-seq was done by transformation of the ENCODE Regulation ‘Txn Factor’ track into a binary matrix (genomic regions × experiments). The analysis including calculation of Pearson correlations between experiments and hierarchical clustering was performed using R functions cor() and hclust(). R scripts used for the entire analysis are available at (https://github.com/gdevailly/ENCODE-TFBS-replicate-quality).

## 3 Results

### 3.1 A third of ENCODE conditions with replicates are of low concordance, while about a fourth has sensitivity issues

We identified 57 conditions within the ENCODE transcription and epigenetic factors ChIP sequencing data where the same experiment was done multiple times (between 2 and 5) for the same factor in the same cell line with the same treatment (or absence of treatment), and the replicates were provided without being merged. For example, the USF1 ChIP-seq in A549 cells treated with 0.02% of ethanol was performed two times by the HudsonAlpha laboratory with the same antibody but with two different library preparation protocols (the “*PCR1x*” and the “*V0422111*” protocols). Two corresponding peak lists are provided by the ENCODE (“*wgEncodeAwgTfbsHaibA549Usf1V0422111Etoh02UniPk.narrowPeak*” and “*wgEncodeAwgTfbsHaibA549Usf1Pcr1xEtoh02UniPk*.*narrowPeak*”) and both experiments are in different lanes in the UCSC track (respective experiment ID numbers 68 and 69, http://hgdownload.cse.ucsc.edu/goldenPath/hg19/encodeDCC/wgEncodeRegTfbsClustered/wgEncodeRegTfbsClustered.bed.gz).

**Fig. 1:**
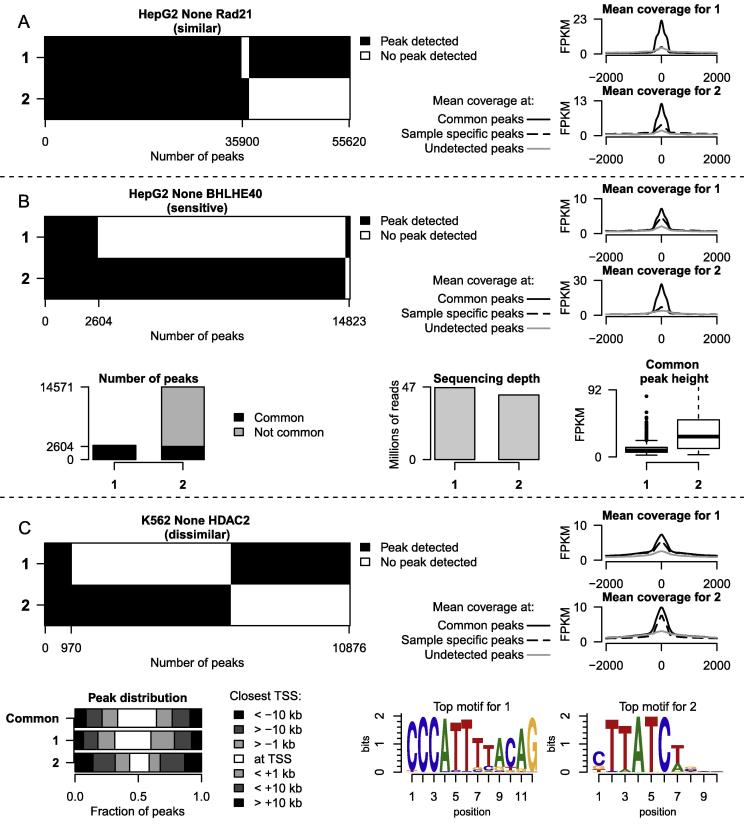
Assessing variability across TF ChIP-seq replicate experiments. *A, B and C:* Examples of the classification based on peak overlap. For each panel, numbers on the *y*-axis represent experiment ID (full names available in Figs. S5–61A). Number on the *x*-axis indicates number of peaks. Black: detected peaks. Grey: no peaks were called in that region. *Coverages plot:* Mean coverage at detected and undetected peaks. Black lines: mean coverage at common peaks. Dash line: mean coverage at detected peaks that were not in common with other experiments. Grey line: mean coverage at undetected peaks that were called only in other experiments. FPKM: Fragments per kilo base per millions. *Number of peaks barplot:* Number of peaks called in each experiment of BHLHE40 ChIP-seq in untreated HepG2. Black: peaks called in every experiment. Grey: peaks not called in every experiment. *Number of reads barplot:* Millions of uniquely aligned reads for each experiment of BHLHE40 ChIP-seq in untreated HepG2. *Common peak height boxplot:* Boxplot of FPKM at common peaks for every experiment of BHLHE40 ChIP-seq in untreated HepG2. *Peak distribution plot:* Distance from the closest TSS was compute for every peak from both experiments of HDAC2 ChIP-seq in untreated K562. Common peaks were frequently overlapping a TSS. Peaks from experiment number 1 were generally closer to a TSS than peaks from experiment number 2. *Motif logos:* Motif logo of the top *de novo* motif discovery results from peak list of experiment number 1 (left) and experiment number 2 (right).

We first compared the peak overlap between replicate experiments to identify a set of common peaks, detected in every replicate. Using the fraction of common peaks in each experiment as a measure of replicate consistency, we classified the 57 conditions in three categories (similar, sensitive and dissimilar). If the overlap between all replicate peak lists and the common peak list was 50% or more (i.e. if at least 50% of the peaks were reproducible in every replicate), then the condition was labelled as “*similar*” (Fig. 1A). Within the non-similar conditions, a special case was made for conditions where one peak list was at least two times bigger than another and more than 70% of the smaller peaks list overlapped with the common peak list. This was labelled as having differences of sensitivity between replicates, and the condition was classified as “*sensitive*” (Fig. 1B) where the differences across replicates might reflect differences in ChIP efficiency, with one experiment with a lower false positive rate, while the other a lower false negative rate. The remaining conditions were labelled as “*dissimilar*” (Fig. 1C). Of the 57 conditions with replicates, 26 were classified as “*similar*”, 13 were “*sensitive*” and 18 were “*dissimilar*” (Table S1). Five of the 18 “dissimilar” conditions used different antibodies suggesting the differences might reflect undocumented isoform specificity of these antibodies. The complete analysis of overlapping peak lists for each condition is available as Figs. S4–60, and is summarized in Table S1.

In order to check whether the discrepancies between peak lists from different replicate experiments might be due to a bias in the peak detection (or peak calling) procedure instead of representing true ChIP enrichment differences, we obtained the raw read counts for each sample from the ENCODE resource. For each of the 135 experiments (from 57 conditions), we computed mean coverage at “*common peaks*” (detected in all replicate experiments targeting a given TF in a given cell line under a given treatment), at peaks detected in a given experiment but not in any other replicate (“*sample specific peaks*”), and at peaks detected in any of the other replicates, but missed in a given experiment (“*undetected peaks*”, Fig. 1, coverage plots). For the majority of 135 experiments, the mean coverage at common and sample specific peaks was higher than at undetected peaks, demonstrating that differences in peaks indeed largely reflect differences in ChIP enrichment signals. In a handful of exceptional cases, mean coverage at undetected peaks was very close to mean coverage at sample specific peaks (experiment 1 for Rad21 ChIP-seq in HepG2, Fig. S53C), or peaks were called in one experiment despite a low read enrichment (experiment 1 for NRSF ChIP-seq in HepG2, Fig. S36C). These exceptions might be due to potential artefacts in peak detection. Mean coverage at common peaks was almost always higher than at sample specific peaks, confirming that intersecting peak lists indeed selects peaks of higher signal to noise ratio. Notable exceptions to this are replicate 2 for CHD1 ChIP-seq in H1-hESC (Fig. S9C), c-Myc ChIP-seq in H1-hESC (Fig. S10C), Pol2 ChIP-seq in K562 (Fig. S50C) and ATF3 ChIP-seq in K562 (Fig. S5C).

Peak lists generated by another peak caller (peakSeq) for 6 replicates (2 similar, 2 sensitive and 2 dissimilar) classified the samples in the same groups (Fig. S1. This emphasizes that differences observed between replicate experiments reflect differences in raw data and they were not introduced by biased peak detection.

### 3.2 ChIP-seq signal-to-noise ratio, but not sequencing depth, is always positively correlated with peak list size

The “*sensitive*” conditions mainly represent conflicts due to a large variation between the numbers of peaks detected across replicates. We therefore systematically investigated criteria responsible for the variability in number of detected peaks. One of the main factors influencing the number of detected peaks is sequencing depth [13]. For each experiment, we thus compared the number of peaks and the number of uniquely aligned reads. Though the number of detected peaks and number of reads frequently showed a positive correlation in general (e.g. Pol2 ChIP-seq in GM12878, Figs. S43D and E), this was not the case for 3 out of 13 (23%) conditions (Fig. S2A): BHLHE40 ChIP-seq in HepG2 (Fig. 1B, number of peaks and millions of reads plots), CEBP ChIP-seq in HepG2 (Figs. S7D and E) and COREST ChIP-seq in K562 (Figs. S13D and E). We also explored if differences in signal over noise ratio (i.e. the level of enrichment obtained after the ChIP) of these ChIP-seq could explain the differences in the number of detected peaks. The FRiP (Fraction of Reads in Peaks) metric used by the ENCODE consortium [5] is sensitive to the number of detected peaks. We therefore computed peak FPKM (Fragments per Kilo base per Million) for each experiment at common peaks detected in every replicate experiments (Fig. 1C, common peak height plot, Figs. S5–61F). For the 13 “*sensitive*” conditions, the relationship between the total number of peaks detected in an experiment and the median peak FPKM at common peaks was always positive (Fig. S2B). This analysis therefore suggests that the level of ChIP enrichment (i.e. the signal over noise ratio) is a better predictor of number of detected peaks than the sequencing depth in a ChIP-seq experiments. Lower ChIP enrichment therefore cannot practically be compensated for by increasing sequencing depth in order to detect more peaks. This also highlights the importance of normalizing data according to their signal over noise ratio, using appropriate tools, such as MAnorm [10] and DiffBind [14], when comparing two peak lists from ChIP-seq of the same factor in different cell types or conditions. We applied MAnorm to find statistically different bound regions between replicate experiments for 6 conditions with replicates (Fig. S1). In three (JUND ChIP-seq in HepG2, YY1 ChIP-seq in GM12878 and Pol2 ChIP-seq in GM12891) of six conditions, more than 50% of the peaks show statistically significant differential binding between replicates. For the other three conditions (RAD21 ChIP-seq in K562, CMYC ChIP-seq in HeLa-S3 and PAX5 ChIP-seq in GM12878), less than 25% of the peaks show statistically significant differential binding between replicates. Thus overall (but not always), MAnorm (or similar methods) can indeed help producing a robust peak list shared by all replicate experiments. However, it should be noted that false positive peaks are likely to display low number of reads in each replicate experiment, and thus are unlikely to be called differentially bound across experiments.

### 3.3 Data analysis and integration to determine biological validity of a sample

The promoter proximity of binding and its preference for specific sequence motifs are characteristics of many transcription factors and can be used as a proxy to establish biological validity of ChIP-seq samples. We annotated peaks with their distance to the nearest transcription start sites (TSS). This revealed differences between replicates, especially for dissimilar conditions (Fig. 1C, peak distribution plot, and Figs. S5–61G). We also performed *de novo* motif discovery on all 135 experiments. Out of the 18 conditions with dissimilar peak lists, 6 (33%) showed a discrepancy between the *de novo* motifs identified in the replicate experiments (Fig. 1C, motif logos, Fig. S3 and Table S1). This was the case for one of the 13 sensitive conditions (8%), and one of the 26 similar conditions (4%). We then systematically investigated the replicates for the dissimilar conditions to determine whether these or any other evidence put higher confidence on one or few replicate(s) over other(s). We first illustrate two cases where additional analysis showed that one replicate appears more relevant than the other.

1. HDAC2 experiments in K562 cell line – histone deacetylase HDAC2 experiments in K562 cell line were generated by the Broad and HudsonAlpha laboratories using different antibodies. When comparing to other ENCODE ChIP-seq experiments, HudsonAlpha HDAC2 ChIP-seq clusters with P300 (as identified by the Sydh laboratory) while Broad HDAC2 clusters with HDAC6 (Fig. S4A). In H1-hESC cell line, HDAC2 (HudsonAlpha antibody) and P300 cluster together as well. The discrepancy between the two HDAC2 experiments is likely to be due to different antibody specificities. Wang et al. [15] identified that a cell-line specific secondary motif that mediates the binding of HDAC2 in K562 was a GATA motif. Accordingly, the GATA motif is the top motif enriched in the HudsonAlpha sample (*P* value < 1*e*−1216), whereas the REST-NRSF motif is the top enriched motif in the Broad sample (*P* value < 1*e*−275). As GATA1 is a master regulator of erythropoiesis and K562 is a leukaemia derived cell line, the HudsonAlpha HDAC2 experiment (and antibody) appears more reliable than the Broad HDAC2 in K562.
2. CMYC experiments in H1-hESC cell line – CMYC replicate experiments in H1-hESC cell line were generated by the Stanford and UTA laboratories. Amongst H1-hESC samples, the Stanford CMYC replicate clusters together with MAX and is part of a larger cluster including RNA polymerase II and other promoter associated factors, such as TAF1 and TBP. This is in accordance with the proven role of CMYC in RNA polymerase II elongation [16]. The UTA CMYC sample in contrast does not cluster with any of the above samples, putting the biological integrity of this sample in doubt. Corroborating this, the CMYC motif is enriched in the Stanford, but not the UTA sample (Figs. S3G and H).

Although further analysis is indeed a way forward as demonstrated above, it does not always provide sufficient evidence to prefer one experiment over other for the same condition. For instance, the two CHD1 ChIP-seq experiments in H1-hESC are dissimilar (Fig. S9A). Interestingly, the “common” peaks did not select for peaks of higher coverage for one of the replicates (Fig. S9C). *De novo* motif discovery detected different primary (Figs. S3D and E) and secondary motifs in each experiment, but neither appears more biologically relevant than the other. When peak overlap of all CHD1 experiments in the ENCODE resource is clustered, neither of the two experiments appear closer than the other to the CHD1 ChIP-seq experiments done in GM12878 and in K562. Taken together, we can only conclude that both experiments are dissimilar, cannot be intersected or merged, and that our current knowledge is insufficient to select the most biologically relevant experiment.

## 4 Discussion

The ENCODE ChIP-seq data is of great value to computational and non-computational biologists alike and is widely used by the scientific community [1], [2]. One great strength of this consortium is that its transparency and extensive data release policy. Taking advantage of this, we noted that the data contains several different replicate experiments for the same factor in the same cell line under the same treatment (or absence of treatment), without indication about the consistency between replicates or recommendations about which peak list to use. We performed an independent assessment of the consistency between these replicate experiments by categorizing the conditions with replicates in three groups: similar, sensitive and dissimilar. We found 18 of 57 showed a very low overlap between peak lists from replicate experiments. Assuming that a discordance is due to only one faulty sample amongst the replicate experiments, a simple extrapolation of these results to all 690 ENCODE ChIP-seq samples puts the biological validity of about 14% of them into question. To examine this, we compared the clustering of binding sites of multiple factors studied by ChIP-seq across cell types. Thirty-eight factors have been studied by ENCODE in both H1-hESC and K562 cell lines. NRSF in K562 clusters together with P300 while it does not cluster together with P300 in H1-hESC. SP1 and SP2 cluster together in K562 while SP1 shows highest peak overlap with P300 in H1-hESC cells. Due to the absence of replicates, it is impossible to know from this data alone whether these factors have a context-specific (cell type specific) binding profile, or if the observed differences are emerging from variable quality between experiments.

The practical question still remains which replicate to use or what is the best way to merge replicates. Intersecting peak lists is the most conservative approach, and retains a small number of high quality peaks. Although the most reasonable approach in most situations, it suffers a drawback: replicating a ChIP-seq experiment will effectively reduce the number of peaks detected. We therefore suggest other strategies, on a case-by-case basis (Table S1). For most conditions in the similar or sensitive category, we suggest to use the union of peak lists. For conditions in the dissimilar category, we used additional analyses or information to suggest one peak list likely to be more reliable than the other(s). For example, for the CMYC ChIP-seq in H1-hESC, we recommend using replicate 1 based on *de novo* motif discovery (Figs. S1E and F). For RNA pol2 phosphoS2 ChIP-seq in K562, we suggest replicate 2 with a higher proportion of peaks downstream of a TSS. In other cases, we prioritized replicate(s) based on the peak overlap with ChIP-seq of the same factor in other cell line(s) or under a different treatment (Fig. S3).

Taken together, this study demonstrates that the high standards set for technical quality control achieved by the ENCODE consortium does not guarantee the robustness of the sample. This highlights a potential caveat concerning mining of the vast amounts of data deposited in the public domain in general, where even high technical quality of the data is not always documented.

## Competing interests

The authors declare no competing interests.

## Appendix A Supplementary data

Supplementary Figs. S1–61 **Fig. S1** ENCODE SPP peaks, ENCODE peakSeq peaks, and MAnorm SPP peaks comparison. For six conditions with replicates, peak overlap between both replicates experiment is compared from SPP peaks (A, D, G, J, M and P) and from peakSeq peaks (B, E, H, K, N and Q). A, D, G, J, M and P panels are the same than in Figure S5-61B. Statistically differentially bound SPP peaks were then identified by MAnorm [10] (C, F, I, L, O and R). MAnorm definition of common peaks is slightly more stringent than the one we used in panels A, D, G, J, M and P, hence the small discrepancies in total peak numbers. **Fig. S2** Influence of sequencing depth and common peaks height on the number of peak detected. Scatterplot of the difference between (x-axis) sequencing depth (ΔReads, A) or median peak height (ΔMedian FPKM, B) and the difference between (y-axis) number of detected peaks (ΔPeaks). Two times the average absolute deviation was taken for conditions of more than two replicates. Black points: Sensitive conditions. Grey points: other (similar and dissimilar) conditions. Dash line: linear regression of the black points (sensitive category). **Fig. S3** Differences in de novo motif discovery from different replicate experiments of 8 experimental conditions. Top de novo motifs identified by Homer is displayed for each experiment for 8 conditions with replicates, as well as the top motif identified from “common” peaks only. **Fig. S4** Comparison of various peak lists with ChIP-seq of the same factor done in other cell lines or conditions. Pearson correlation coefficient have been calculated between pairs of HDAC2, HDAC6, eGDP-HDAC8 and P300 (A), P300 (B), ATF3 (C), NRSF (D), and RNA Pol2 (E) peak lists in various cell lines of the ENCODE dataset. Results is shown as heatmaps. Colour code indicates the strength of the correlation: Blue: −0.5, white: 0, red: 0.5, black: 1. **Figs. S5–S61** Systematic analysis of each ChIP-seq of the same factor in the same cell line under the same treatment. A. Bold text: cell line, treatment (none = no treatment) and targeted factor of the ChIP-seq experiments. Parenthesis: replicate consistency classification. Box: name of each experiment according to ENCODE at UCSC. B. Number on the x-axis indicates number of peaks. Black: detected peaks. Grey: no peaks were called in that region. C. Mean coverage at peaks for each experiment. Black lines: mean coverage at common peaks. Dash line: mean coverage at detected peaks that were not in common with other experiments. Grey line: mean coverage at undetected peaks that were called only in other experiments. FPKM: Fragments per kilo base per millions. D. Number of peaks called in each experiment. Black: peaks called in every experiment. Grey: peaks not called in every experiment. E. Millions of uniquely aligned reads for each experiment. F. Boxplot of FPKM at common peaks for every experiments. G. Proportion of peaks according to their distance from the closest TSS, for common peaks and for each experiments.

Supplementary Table S1 Summary of the analysis. Column descriptions: Condition: Cell line, treatment (none = no treatment) and targeted factor of the ChIP-seq experiments. Number of replicates: number of ENCODE ChIP-seq targeting the same factor in the same cell line under the same treatment. Lab: Were the experiments done in multiple laboratories? Antibody: Were the experiments used different antibodies? Protocol: Were the experiments done with different protocols for ChIP and/or library preparation? Classification: Classification according to consistency between peak lists (see main text for details). ENCODE quality flag: experiment quality flag according to ENCODE. Consistency in motif discovery: Consistency between *de novo motif* discovery from each peak lists. Advised peak list: Peak list that may be used for future analysis. Reasons: Why we recommend these peak lists.

Supplementary Table S2 Metadata for each experiments. Column descriptions: Lab: Code name of the laboratory of origin of the experiment. Cell: Cell line name. Treatment: Treatment applied in cell culture (none = no treatment). Antibody: ENCODE code for unique antibody. This differs from column TF only when different antibodies where used in different experiments. TF: Name of the targeted factor. Name: UCSC file name code for the experiment. RepName: code name for the condition in which each replicate experiments belongs to. Accession: ENCODE portal accession code for each experiment.

Supplementary file advisedPeakLists.zip This zip archive contains 56 advised peak lists (see main text and supplementary table 1). Files are bed like tab delimited text files, with 4 columns: chromosome, peak start, peak end, and peak score.
