# Supplementary material for: Variable reproducibility in genome-scale public data: A case study using ENCODE ChIP sequencing resource
Source: FEBS Lett. 2015 Dec 21;589(24):3866–70. doi: 10.1016/j.febslet.2015.11.027 (PMC4686001; doi:10.1016/j.febslet.2015.11.027)
Supplement: Supplementary Figs. S1–61 — Fig. S1 ENCODE SPP peaks, ENCODE peakSeq peaks, and MAnorm SPP peaks comparison. For six conditions with replicates, peak overlap between both replicates experiment is compared from SPP peaks (A, D, G, J, M and P) and from peakSeq peaks (B, E, H, K, N and Q). A, D, G, J, M and P panels are the same than in Figure S5-61B. Statistically differentially bound SPP peaks were then identified by MAnorm [10] (C, F, I, L, O and R). MAnorm definition of common peaks is slightly more stringent than the one we used in panels A, D, G, J, M and P, hence the small discrepancies in total peak numbers. Fig. S2 Influence of sequencing depth and common peaks height on the number of peak detected. Scatterplot of the difference between (x-axis) sequencing depth (ΔReads, A) or median peak height (ΔMedian FPKM, B) and the difference between (y-axis) number of detected peaks (ΔPeaks). Two times the average absolute deviation was taken for conditions of more than two replicates. Black points: Sensitive conditions. Grey points: other (similar and dissimilar) conditions. Dash line: linear regression of the black points (sensitive category). Fig. S3 Differences in de novo motif discovery from different replicate experiments of 8 experimental conditions. Top de novo motifs identified by Homer is displayed for each experiment for 8 conditions with replicates, as well as the top motif identified from “common” peaks only. Fig. S4 Comparison of various peak lists with ChIP-seq of the same factor done in other cell lines or conditions. Pearson correlation coefficient have been calculated between pairs of HDAC2, HDAC6, eGDP-HDAC8 and P300 (A), P300 (B), ATF3 (C), NRSF (D), and RNA Pol2 (E) peak lists in various cell lines of the ENCODE dataset. Results is shown as heatmaps. Colour code indicates the strength of the correlation: Blue: −0.5, white: 0, red: 0.5, black: 1. Figs. S5–S61 Systematic analysis of each ChIP-seq of the same factor in the same cell line under the same treatment. A. Bold text: ce [file mmc1.pdf]

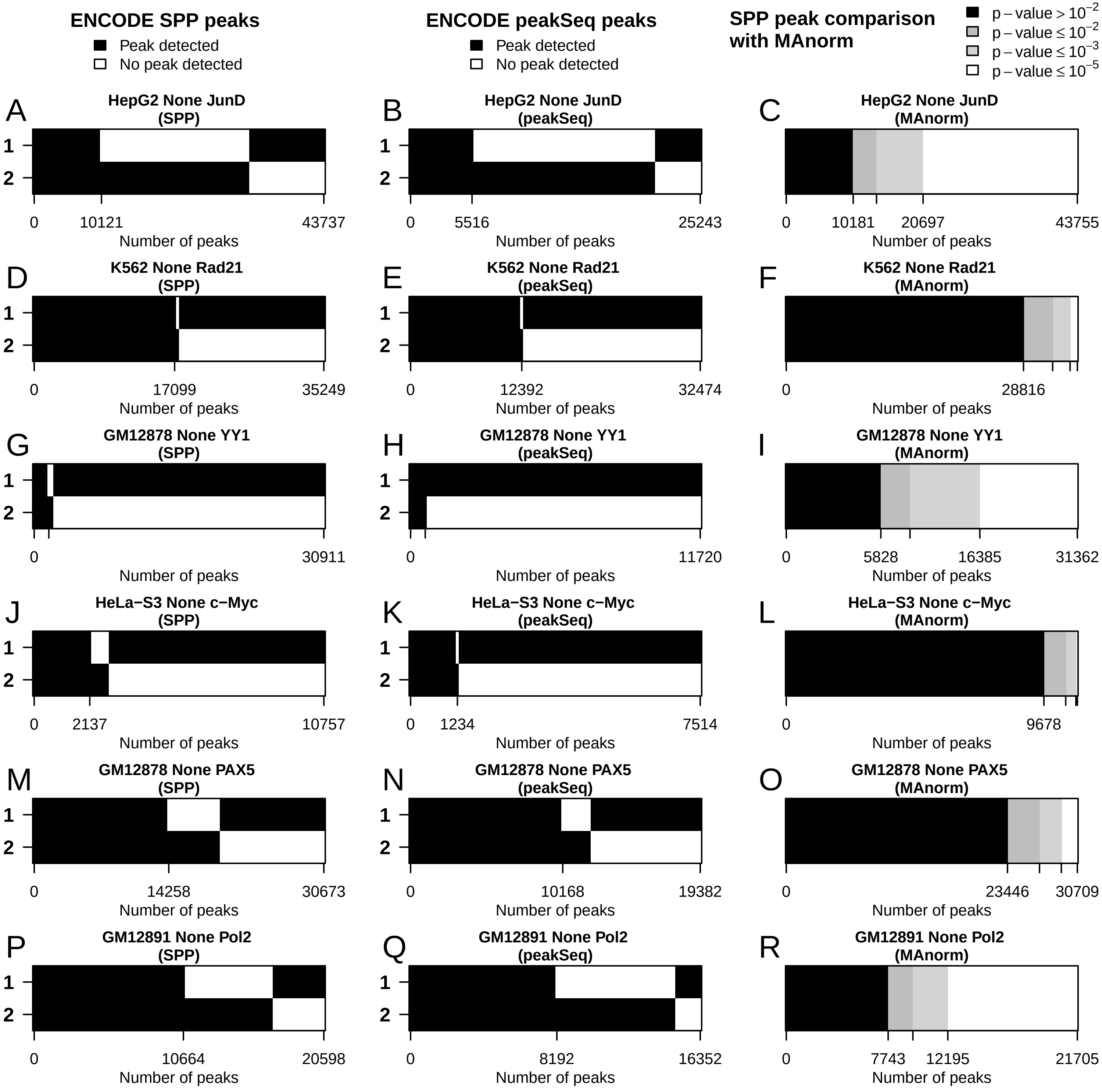

Supplementary figure 1: ENCODE SPP peaks, ENCODE peakSeq peaks, and MAnorm SPP peaks comparison.

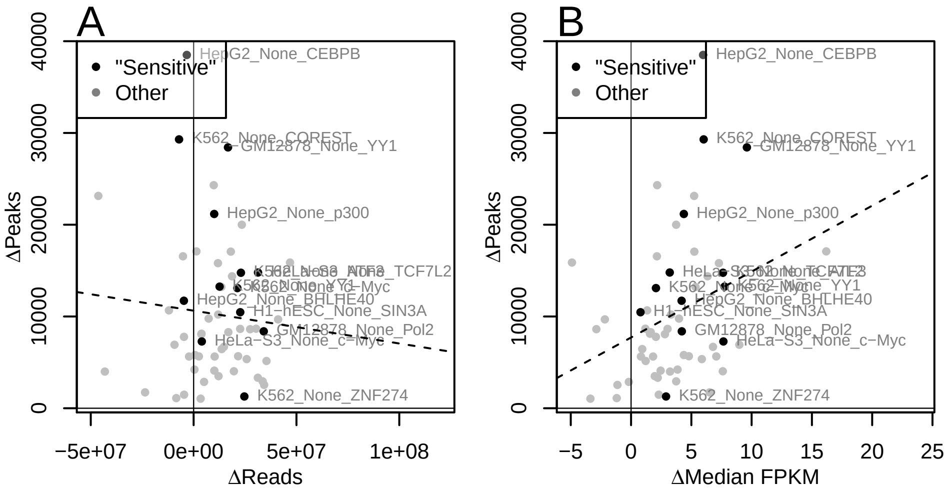

Supplementary figure 2: Influence of sequencing depth and common peaks height on the number of peaks detected

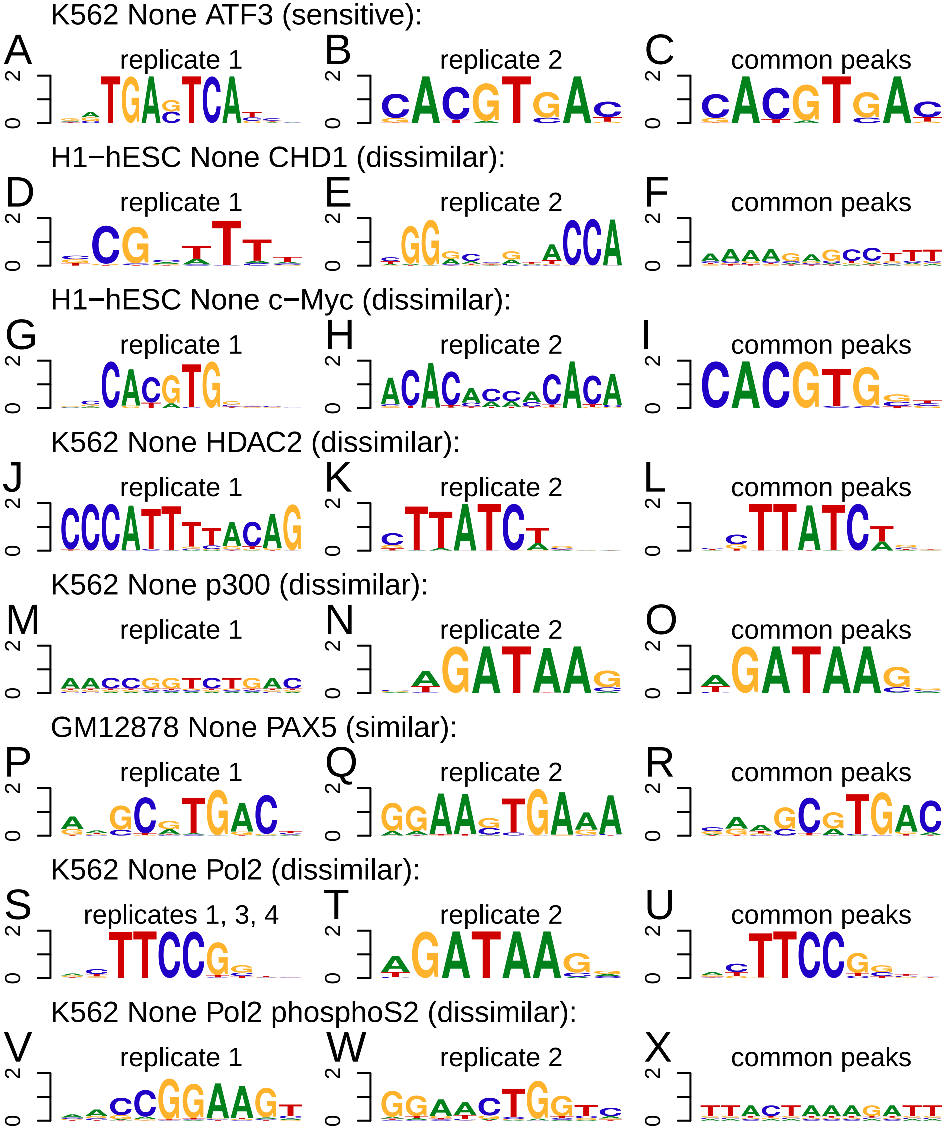

Supplementary figure 3: Differences in de novo motif discovery from different replicate experiments of 8 experimental conditions.

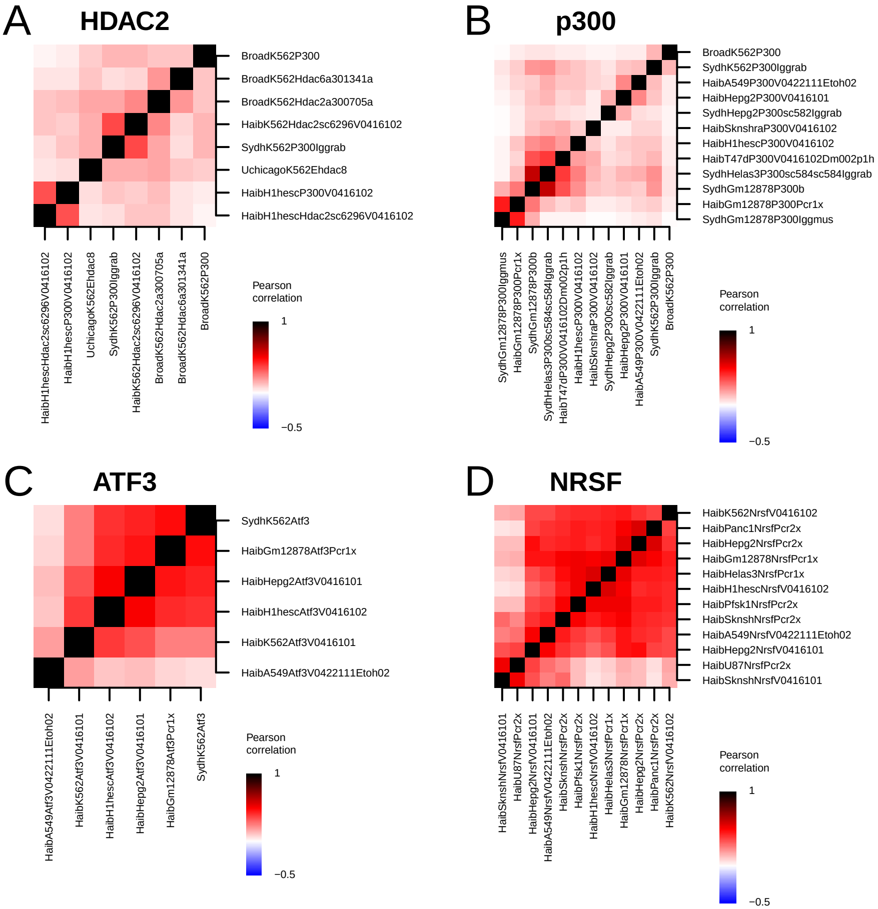

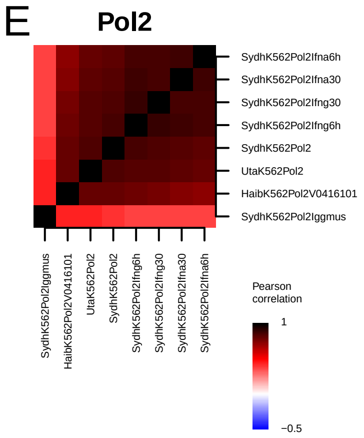

Supplementary figure 4: Comparison of various peak lists with ChIP−seq of the same factor done in other cell lines or conditions

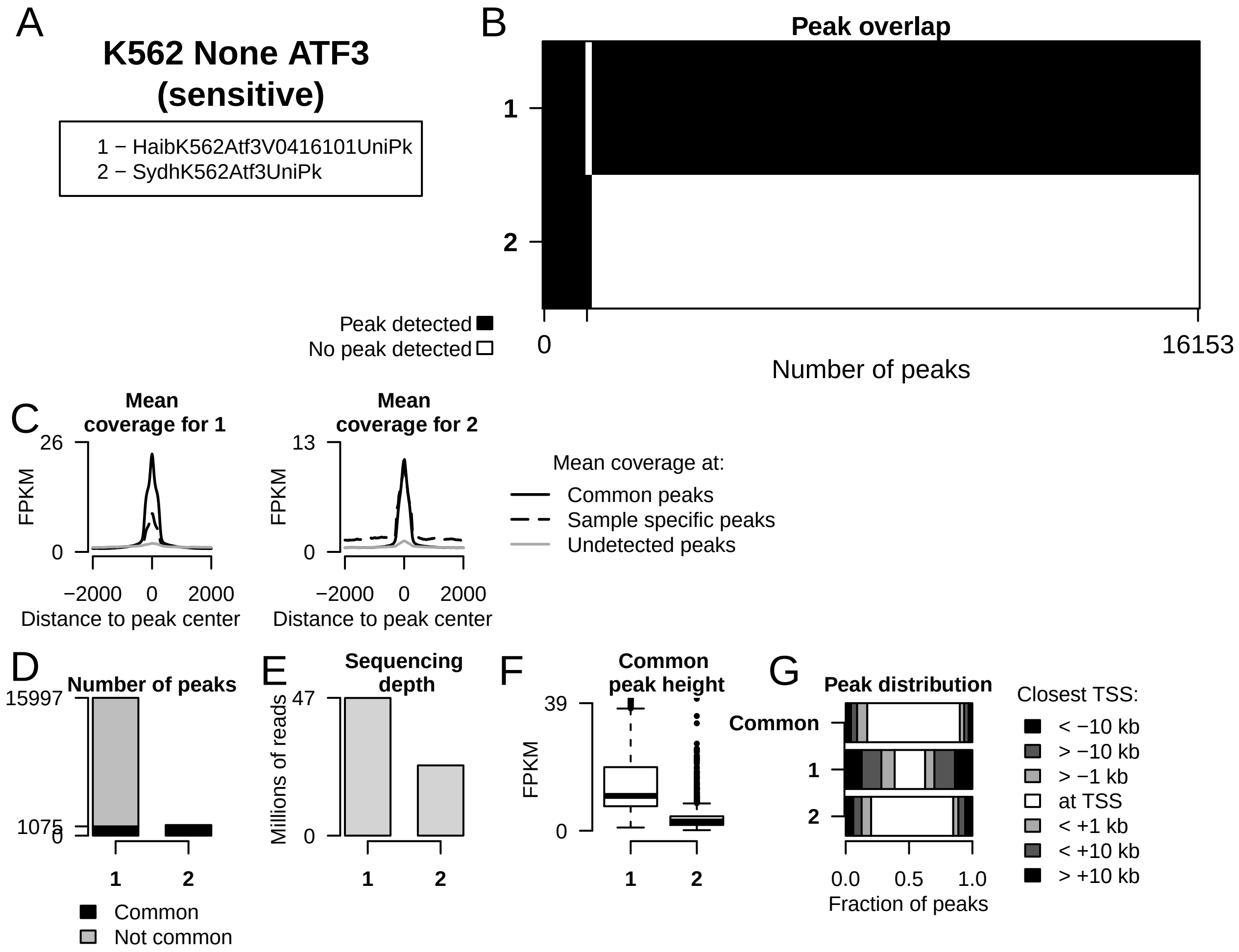

Supplementary figure 5: K562 None ATF3 (sensitive)

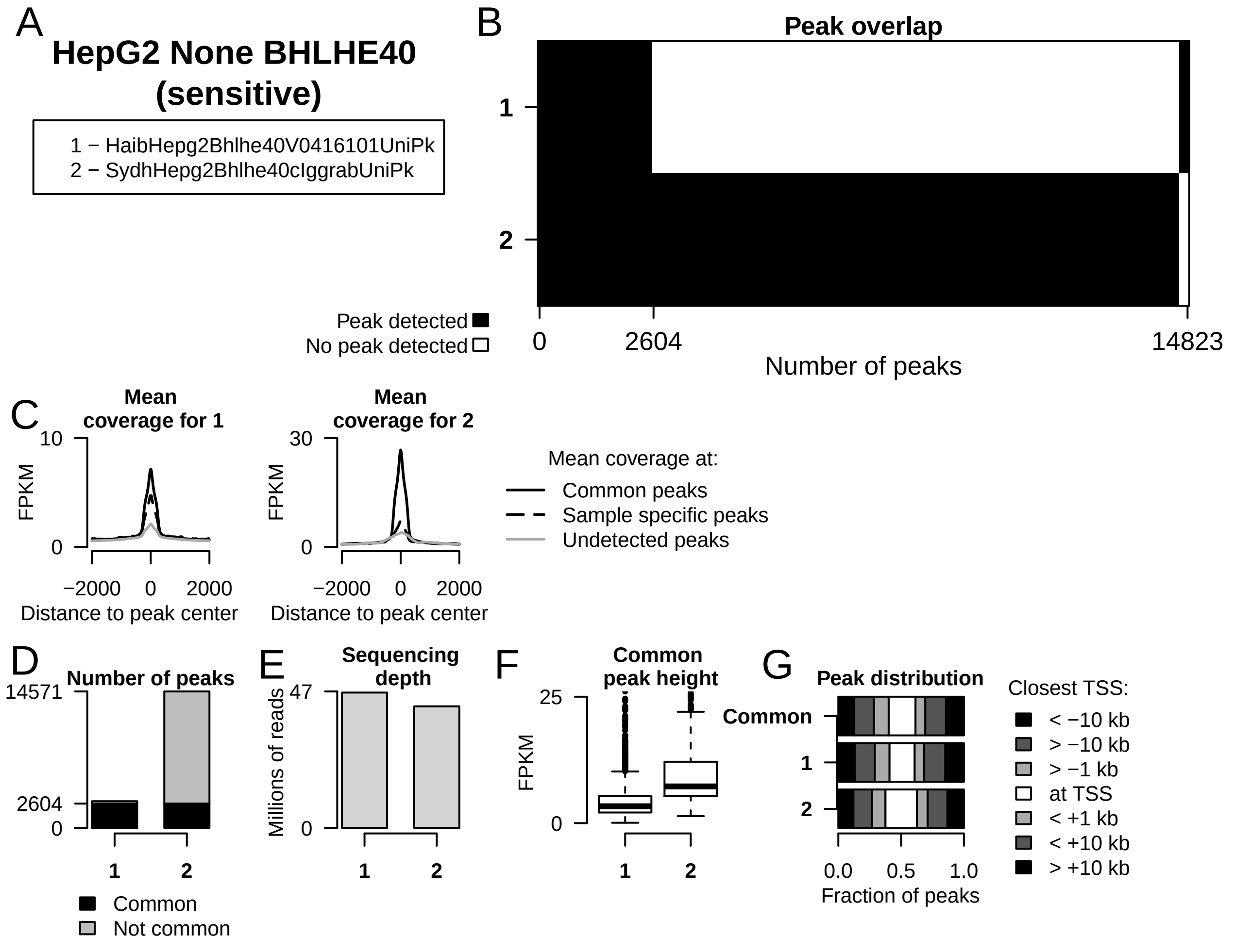

Supplementary figure 6: HepG2 None BHLHE40 (sensitive)

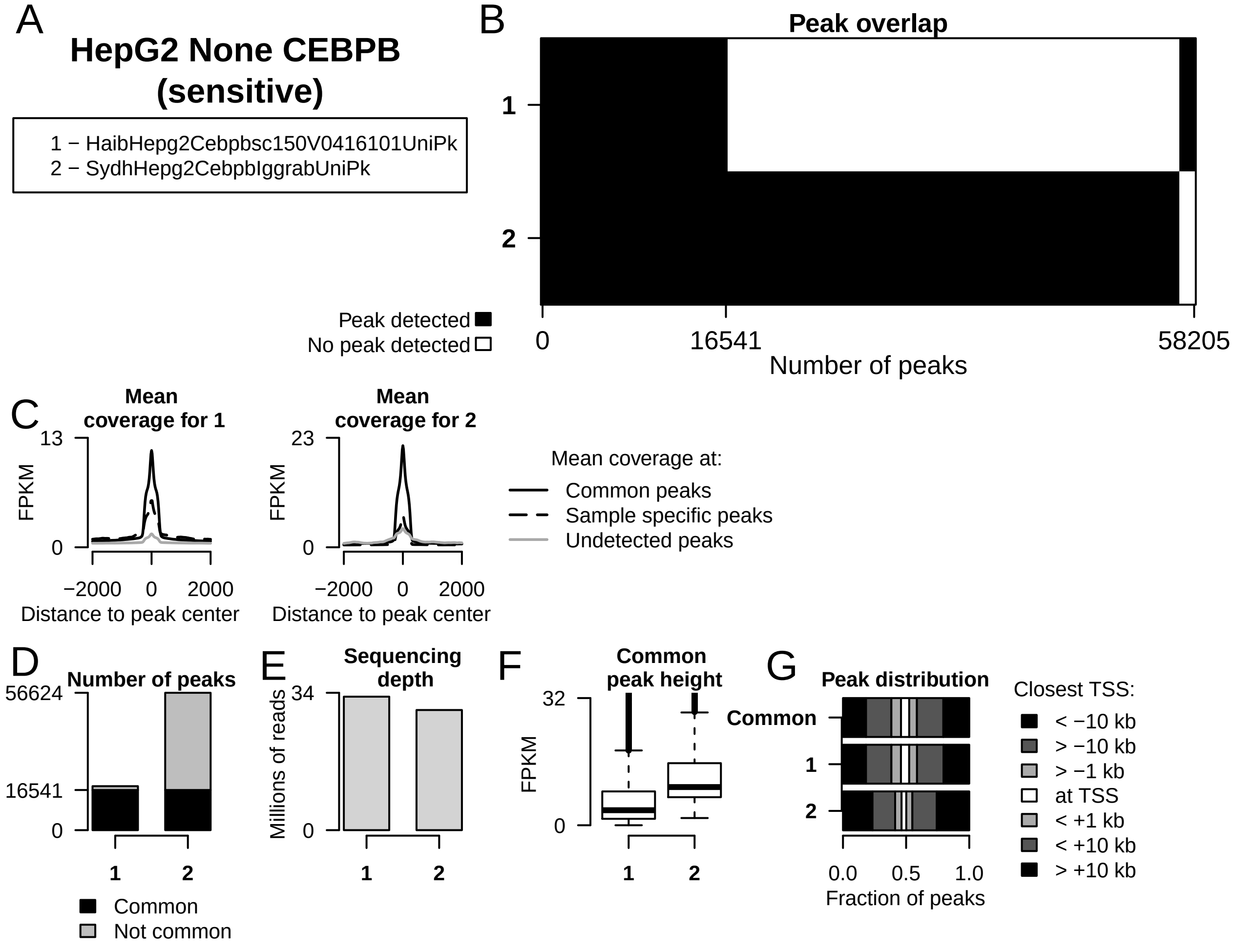

Supplementary figure 7: HepG2 None CEBPB (sensitive)

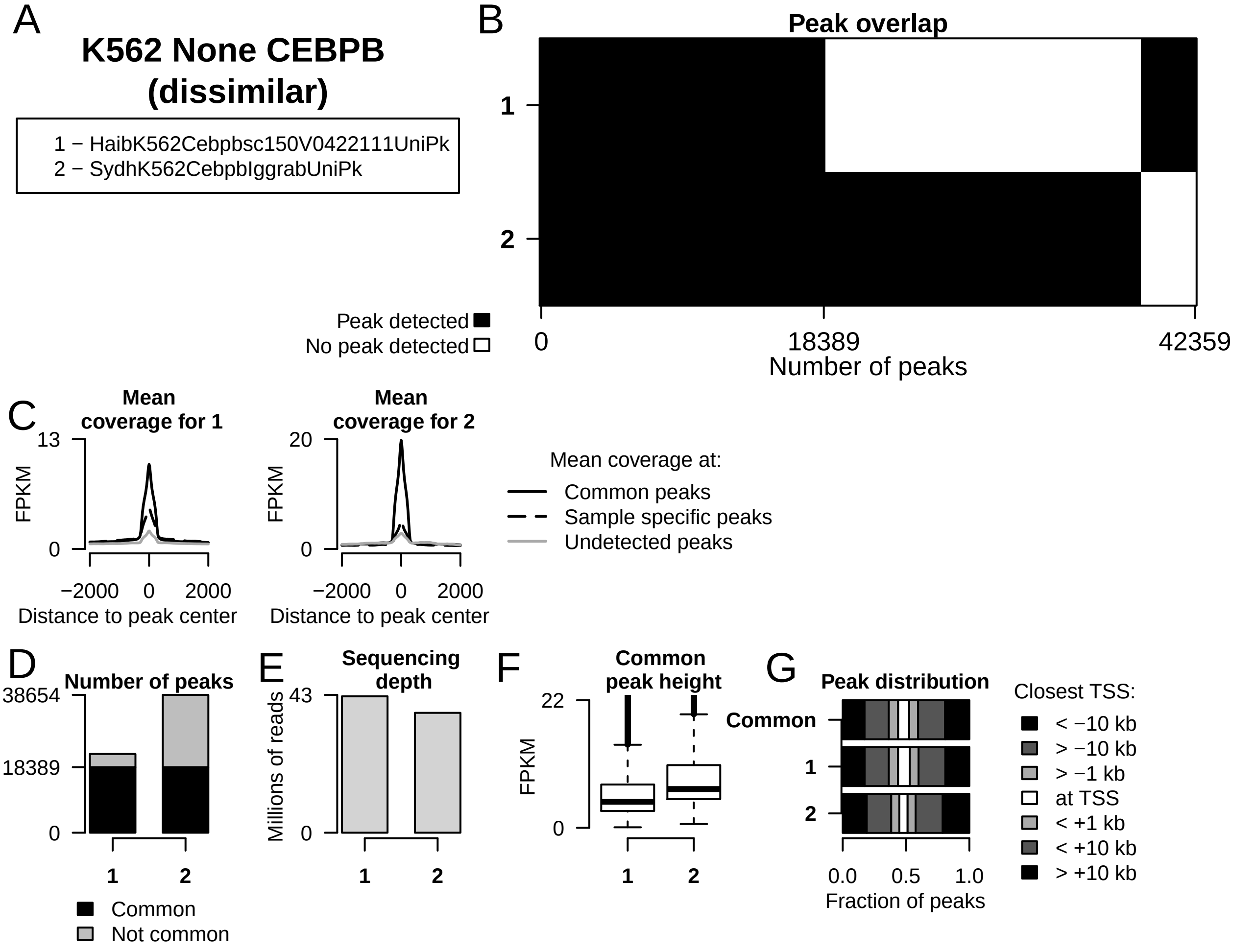

Supplementary figure 8: K562 None CEBPB (dissimilar)

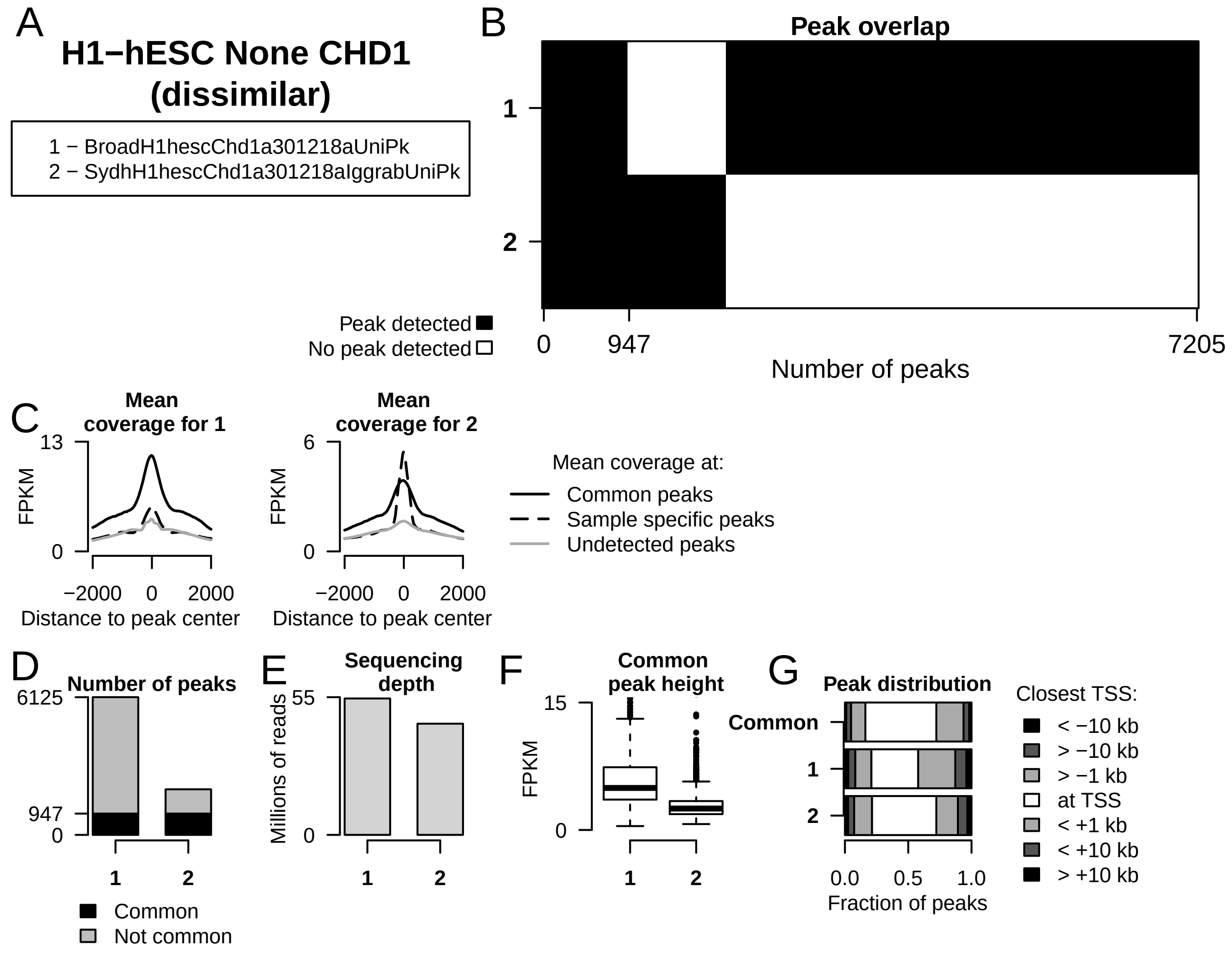

Supplementary figure 9: H1−hESC None CHD1 (dissimilar)

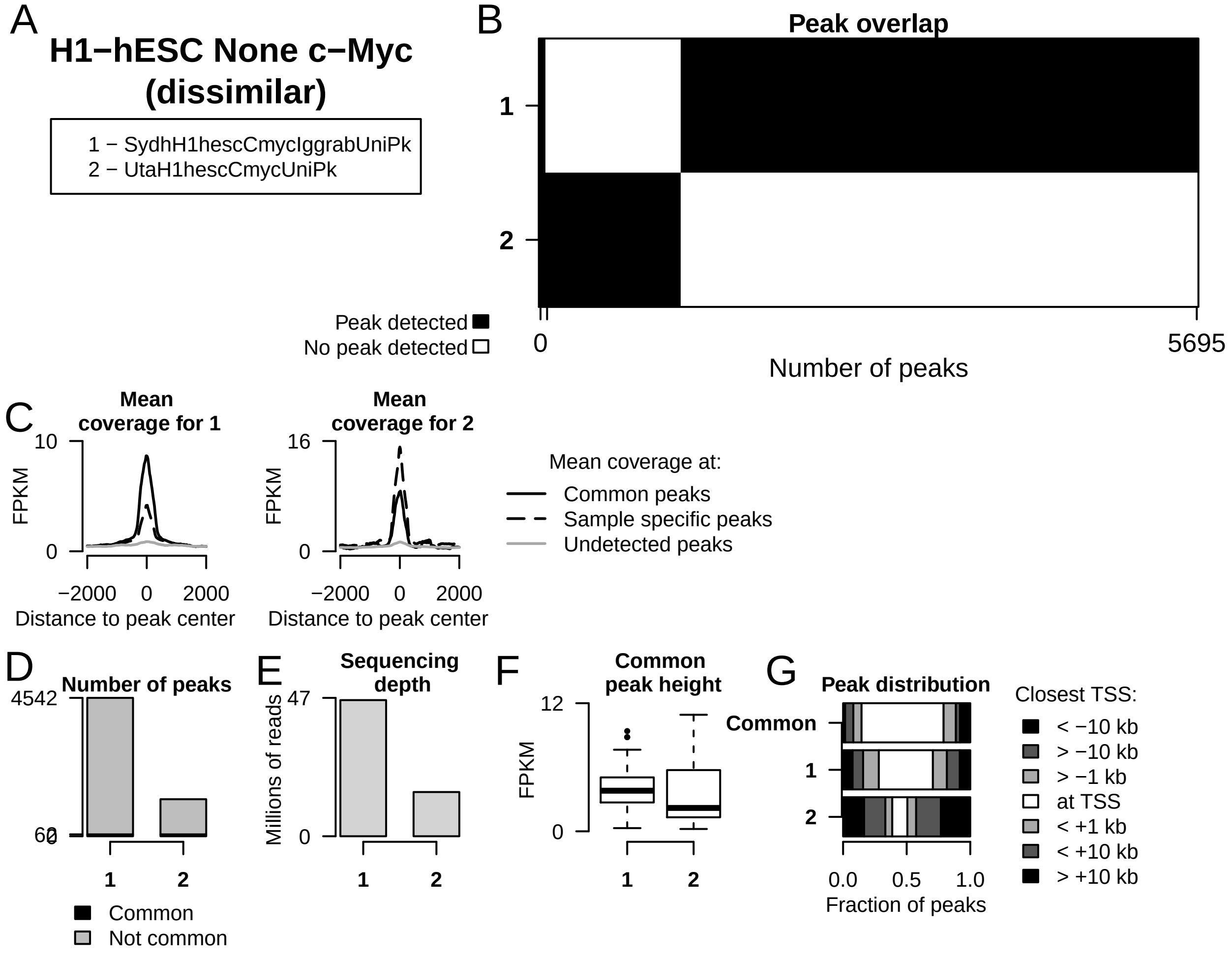

Supplementary figure 10: H1−hESC None c−Myc (dissimilar)

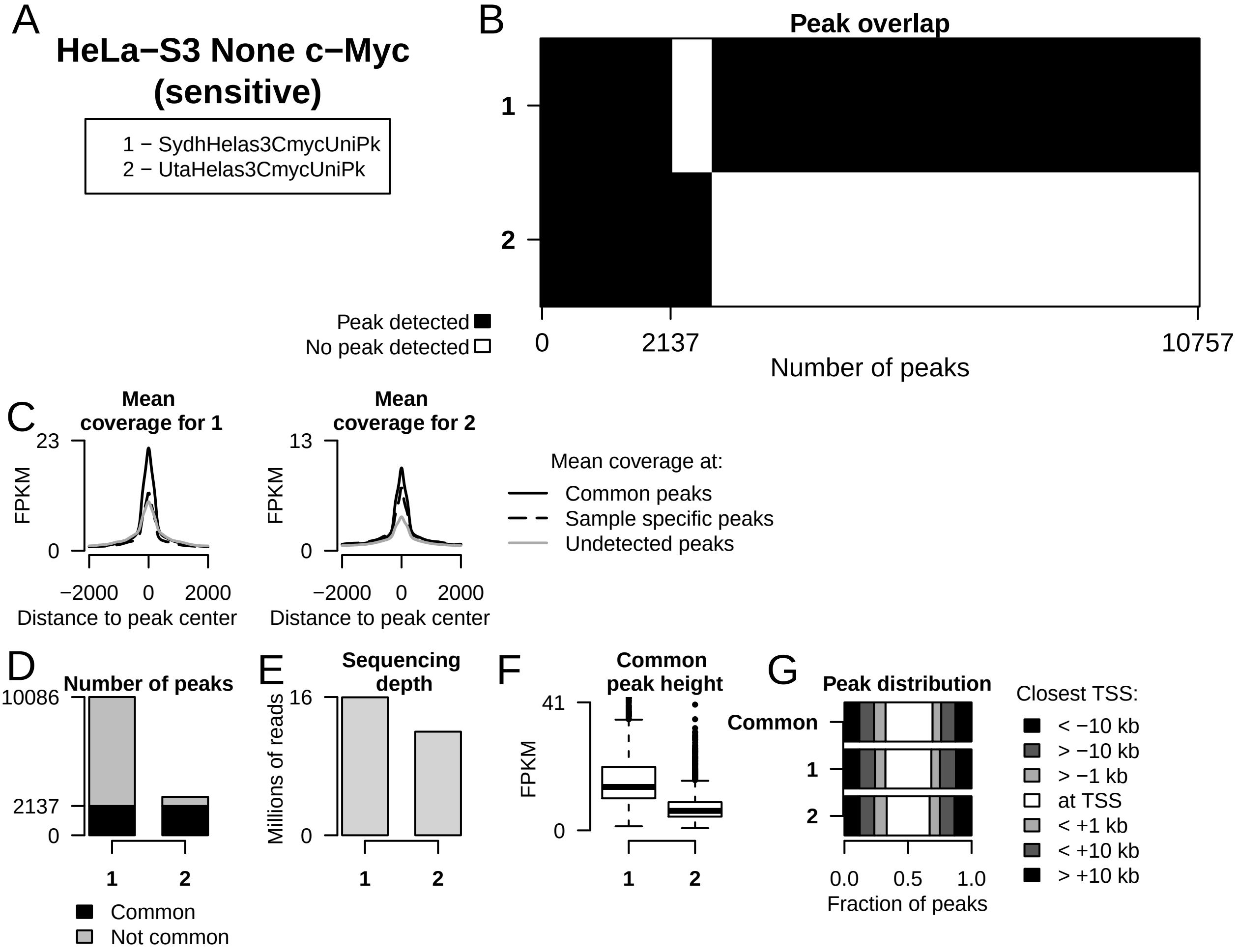

Supplementary figure 11: HeLa−S3 None c−Myc (sensitive)

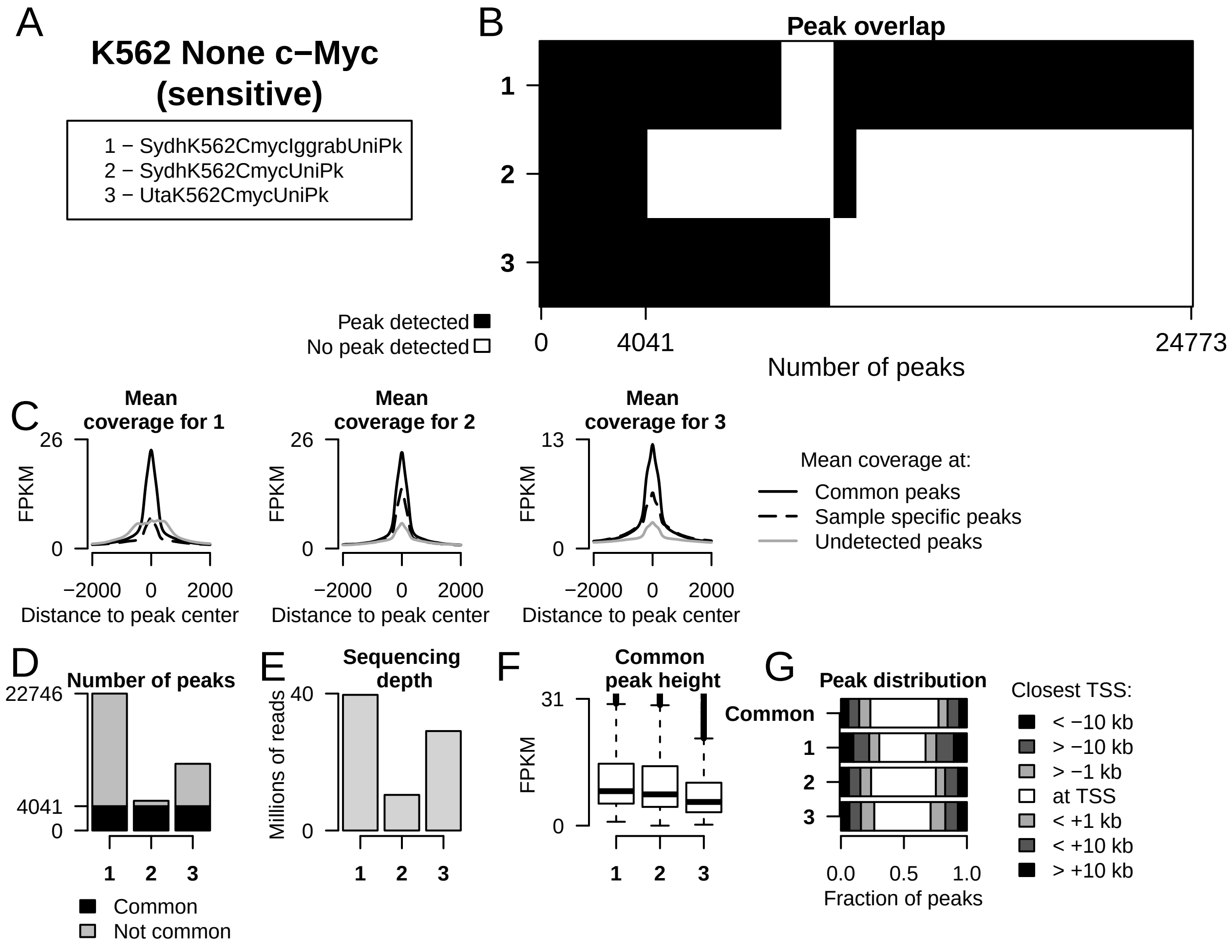

Supplementary figure 12: K562 None c−Myc (sensitive)

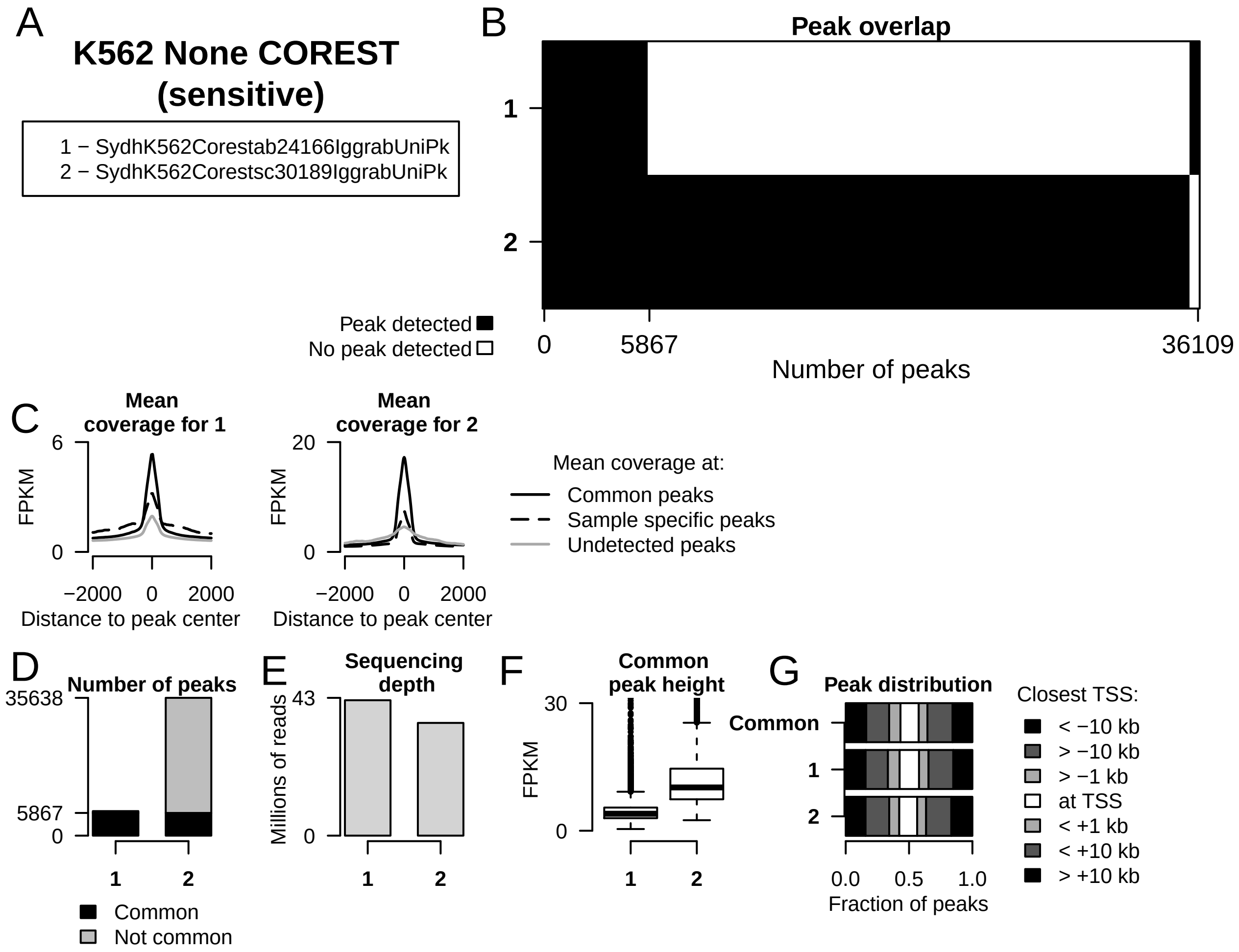

Supplementary figure 13: K562 None COREST (sensitive)

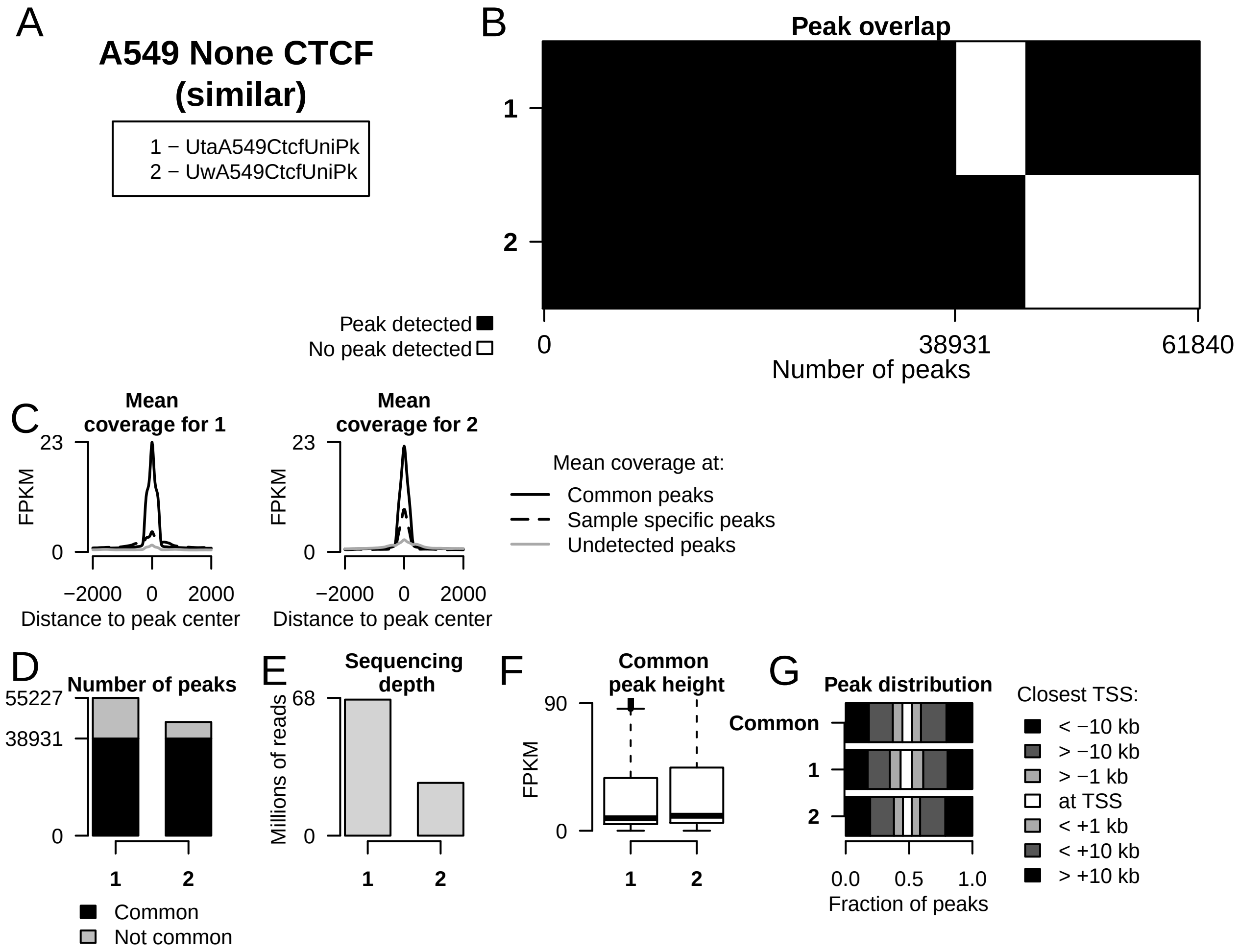

Supplementary figure 14: A549 None CTCF (similar)

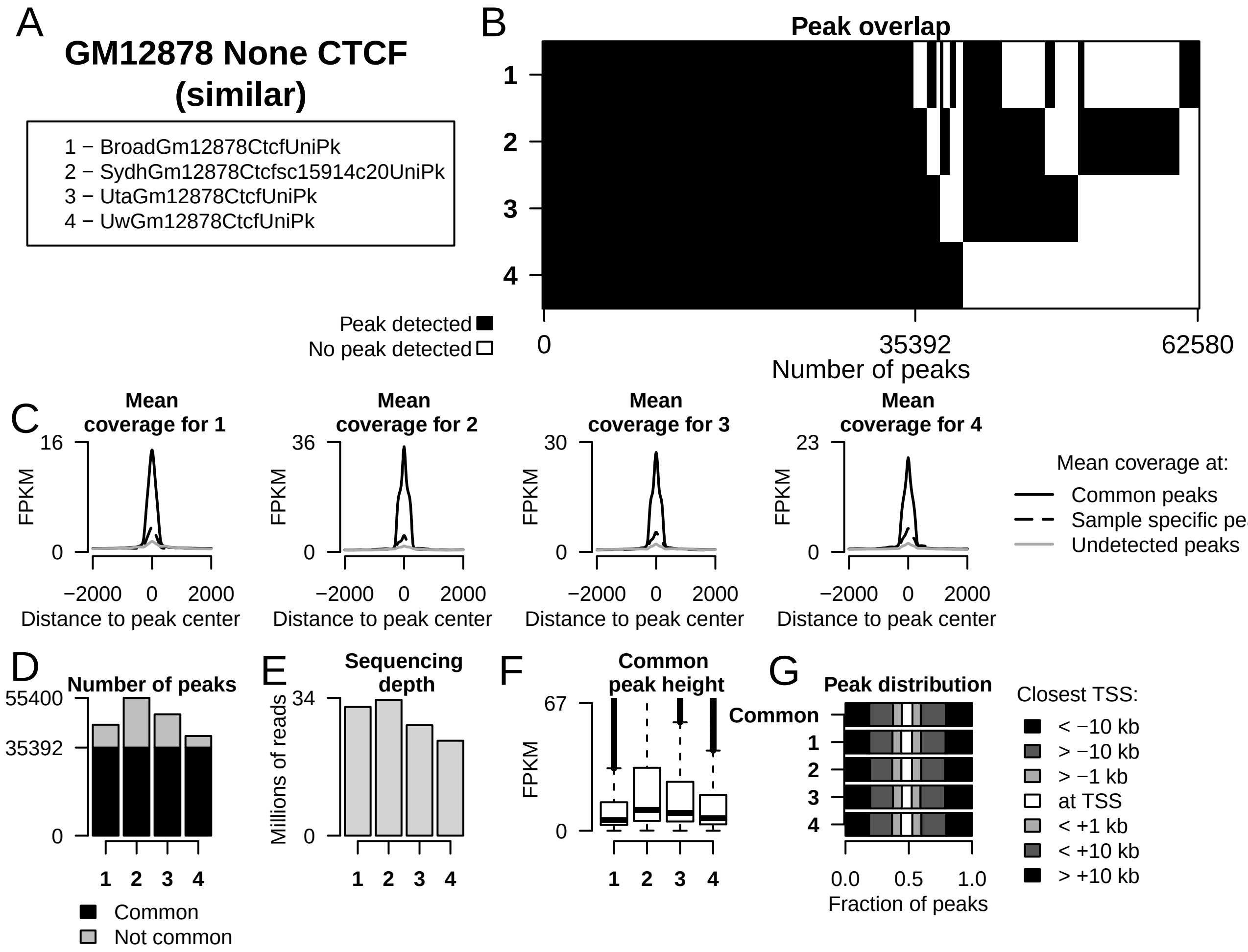

Supplementary figure 15: GM12878 None CTCF (similar)

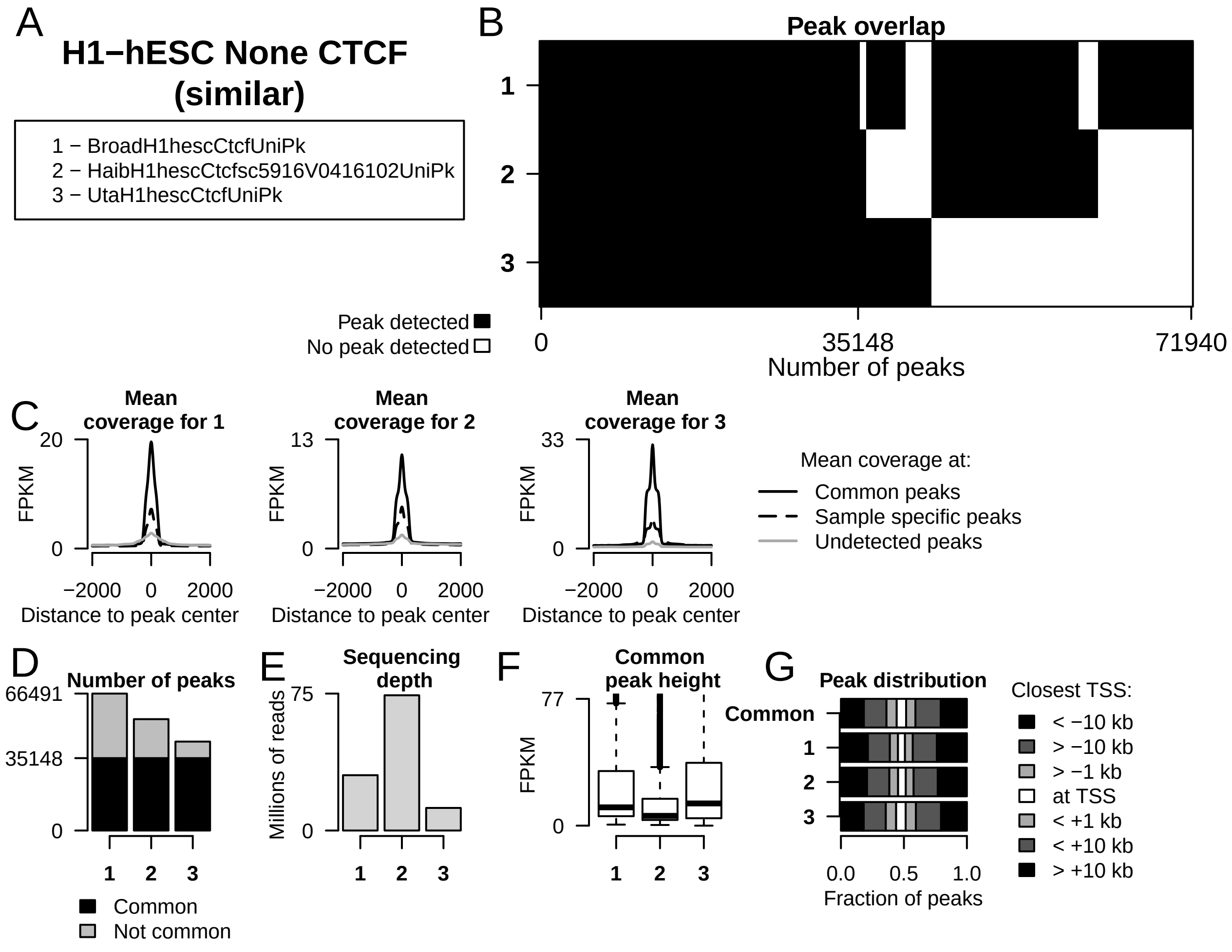

Supplementary figure 16: H1−hESC None CTCF (similar)

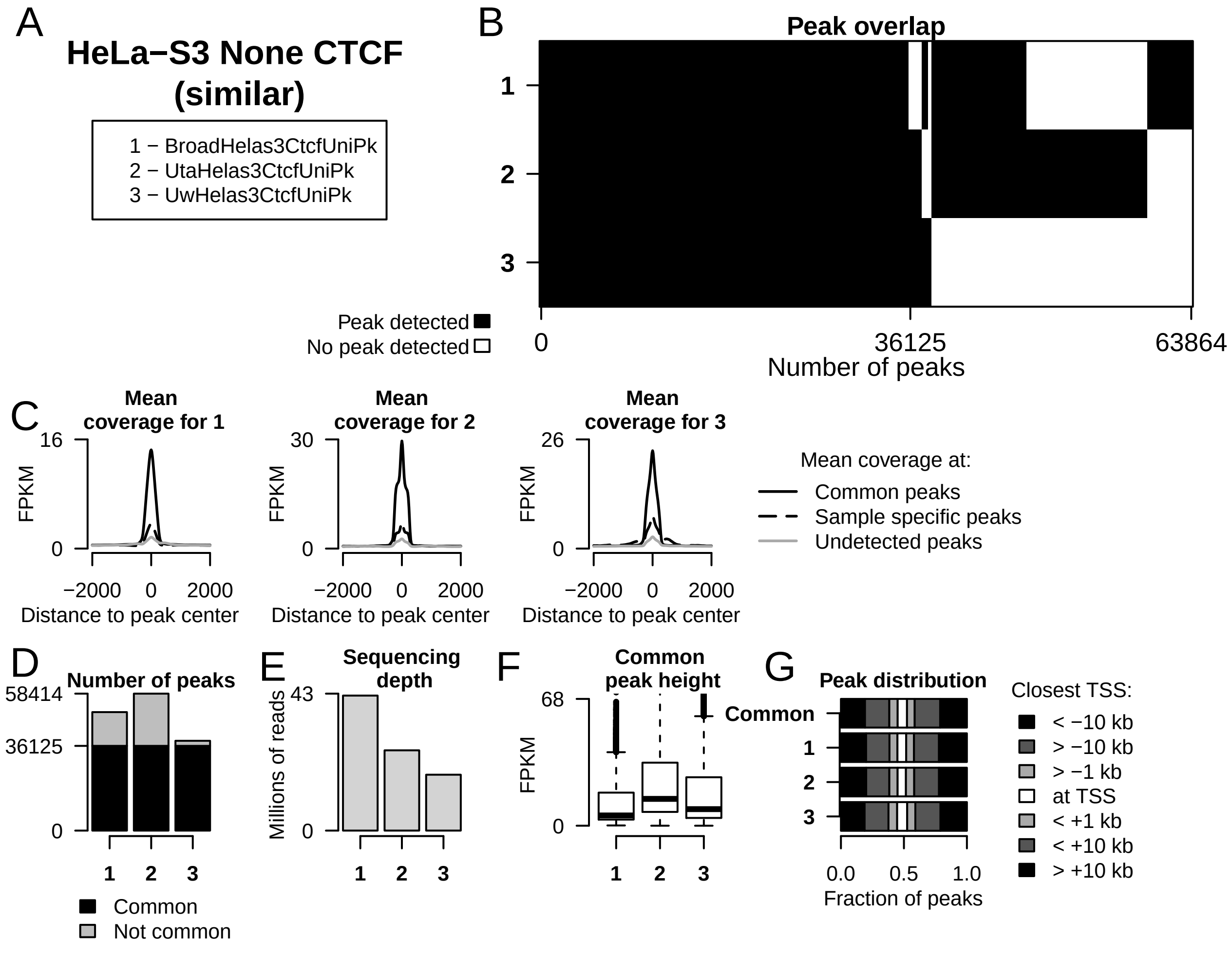

Supplementary figure 17: HeLa−S3 None CTCF (similar)

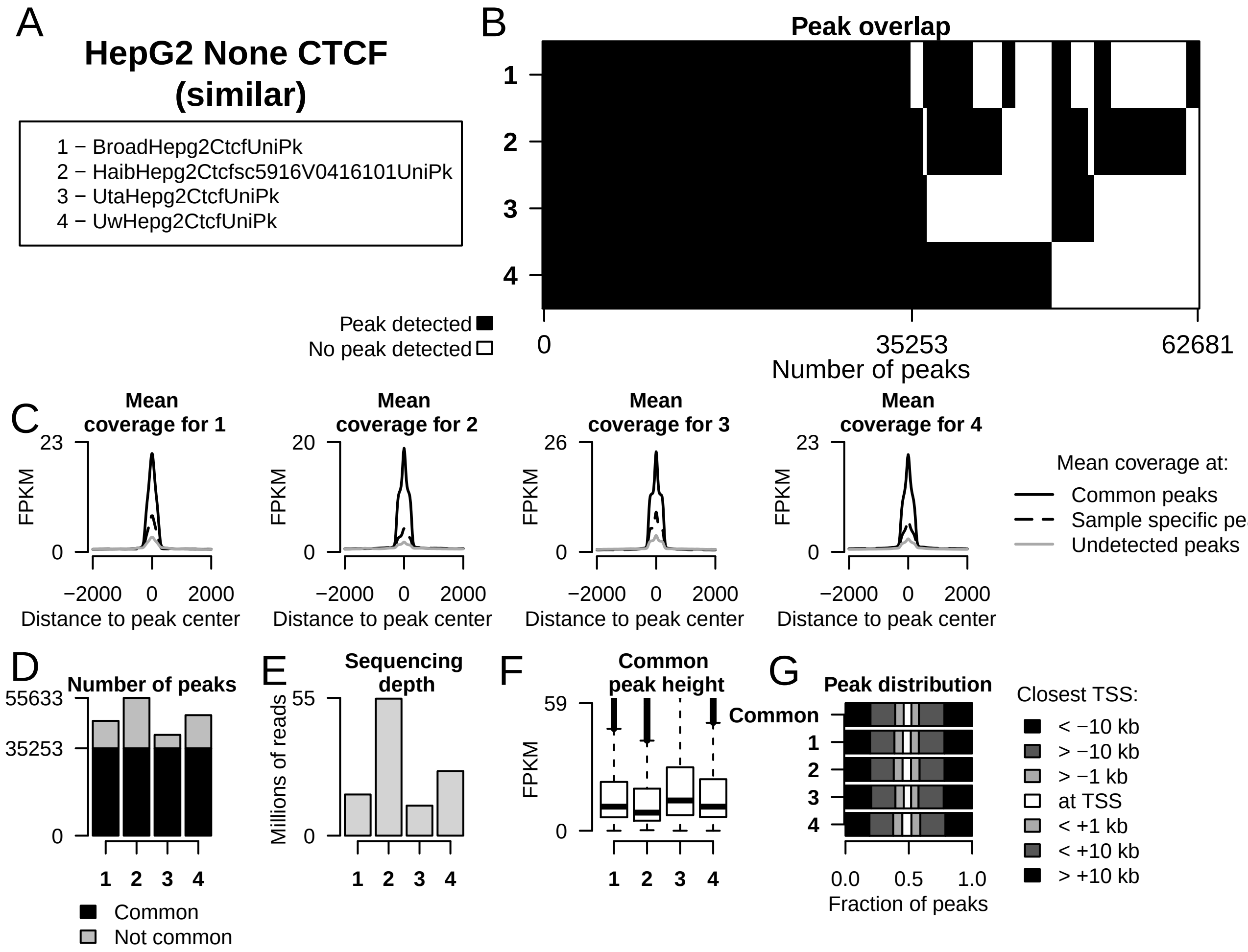

Supplementary figure 18: HepG2 None CTCF (similar)

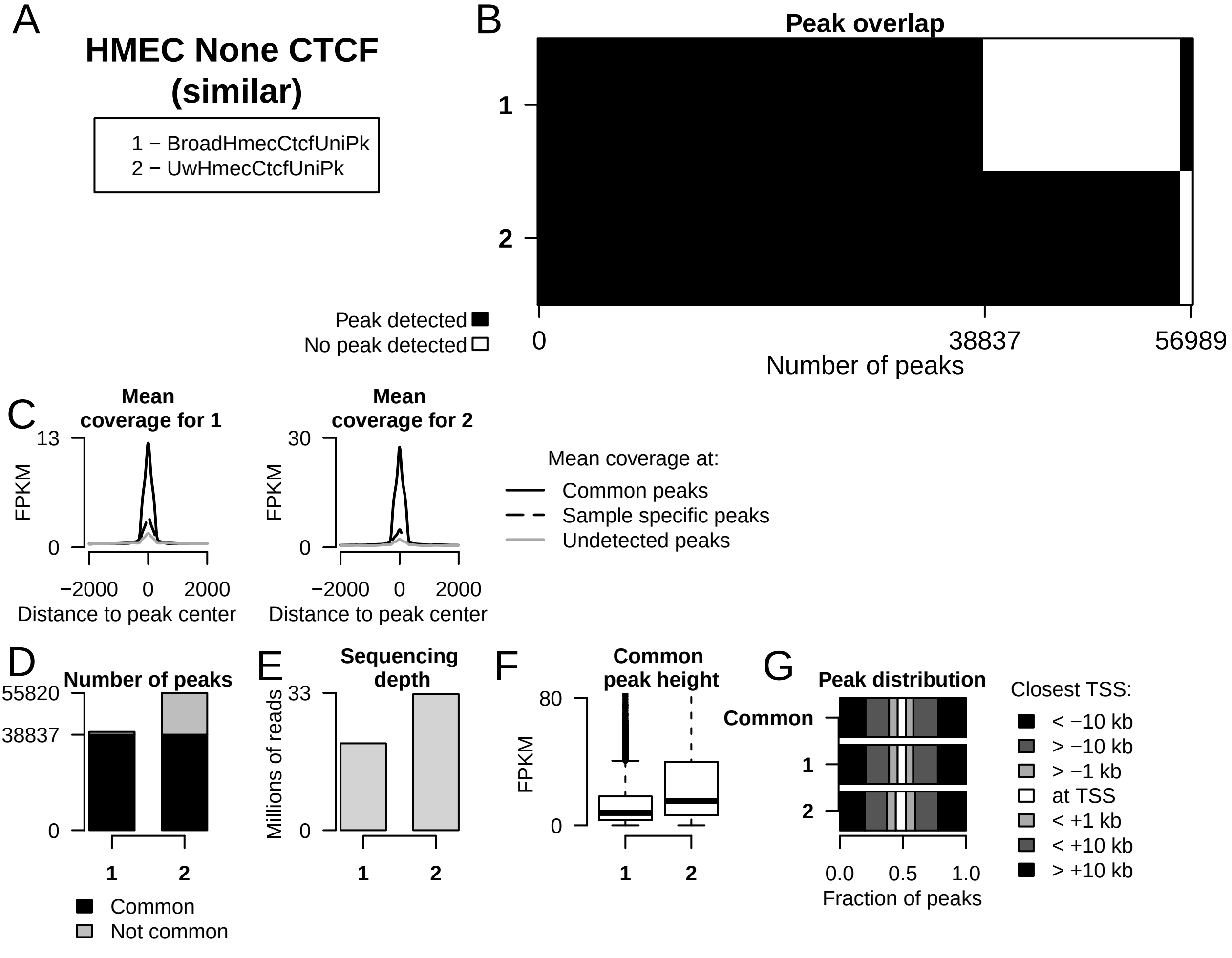

Supplementary figure 19: HMEC None CTCF (similar)

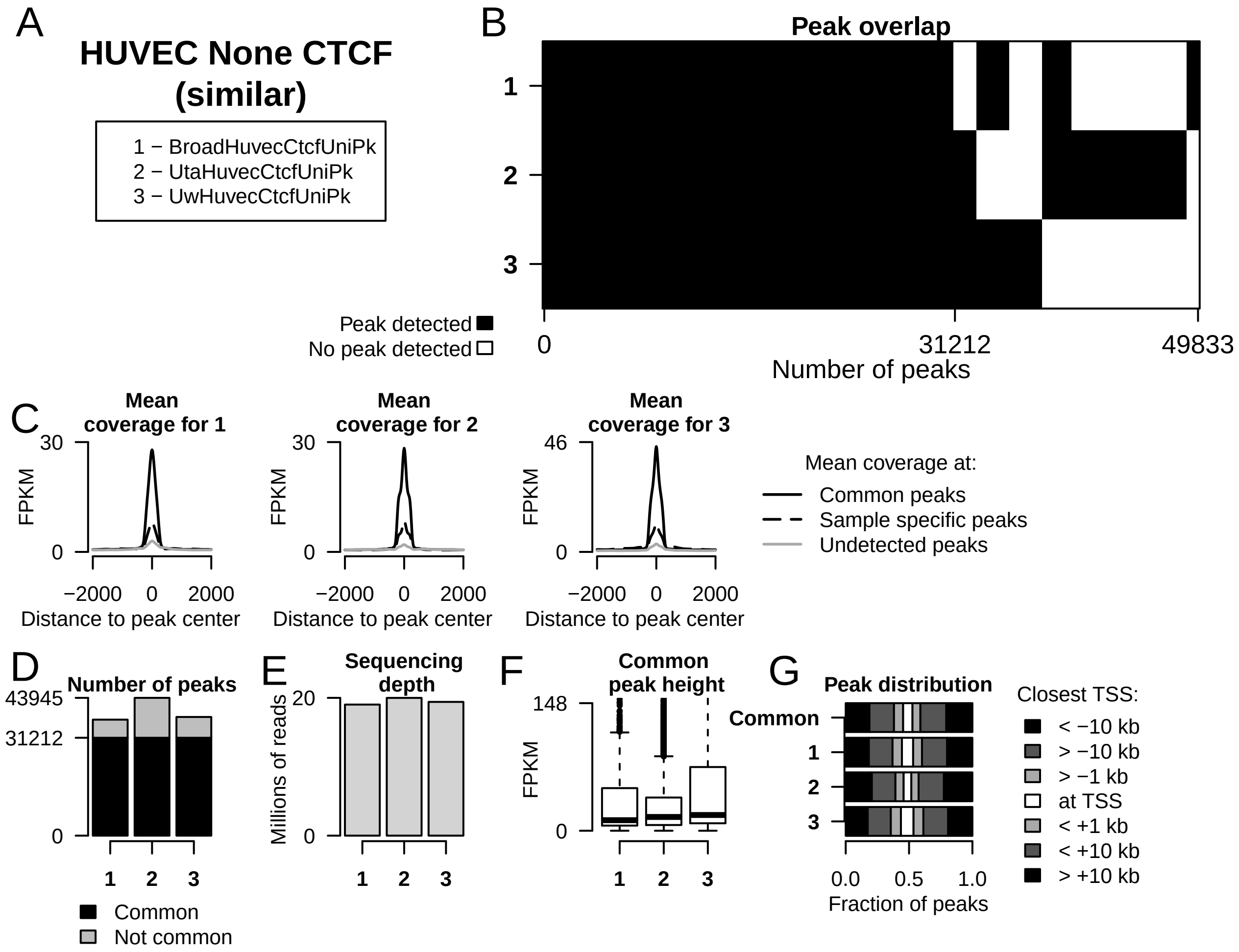

Supplementary figure 20: HUVEC None CTCF (similar)

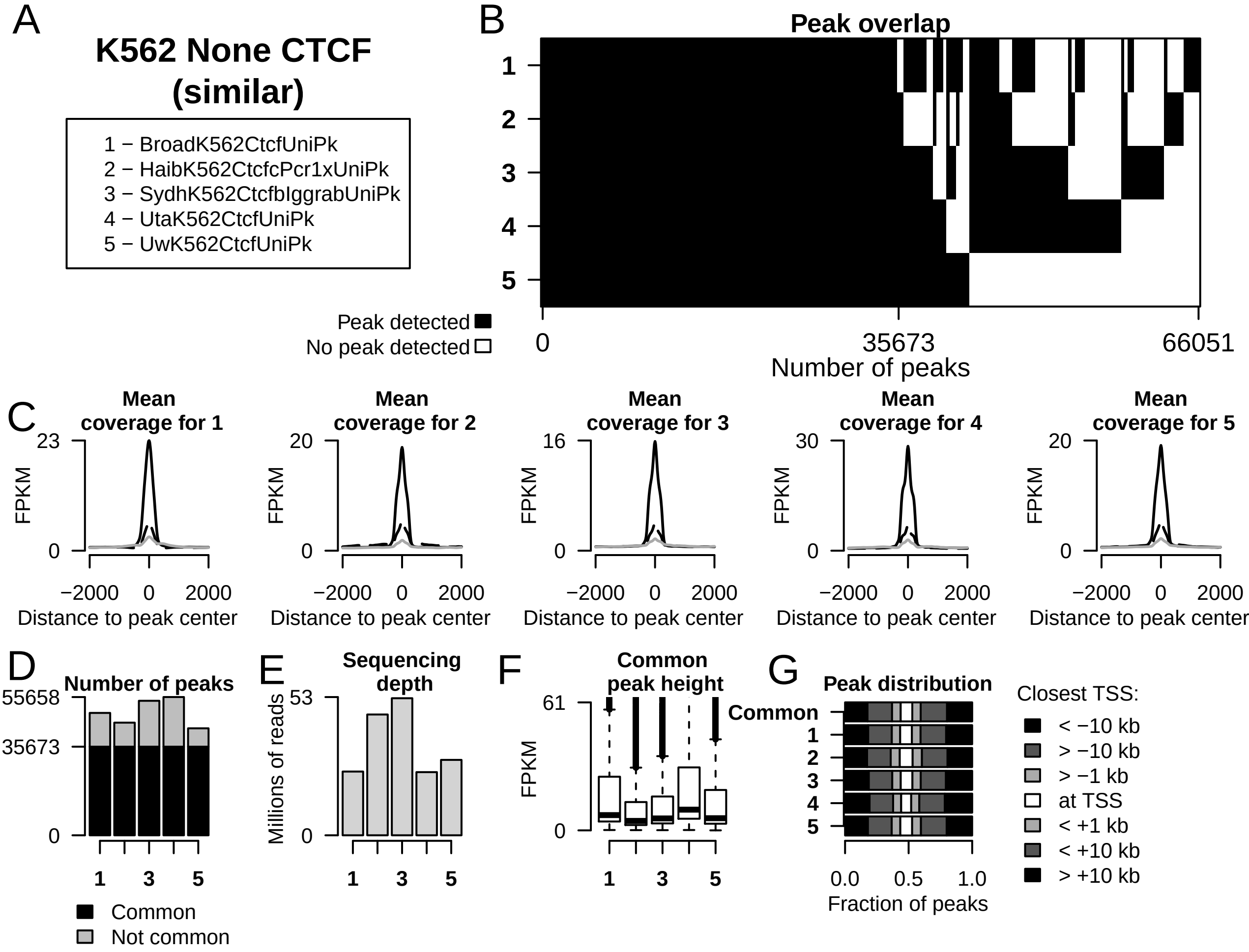

Supplementary figure 21: K562 None CTCF (similar)

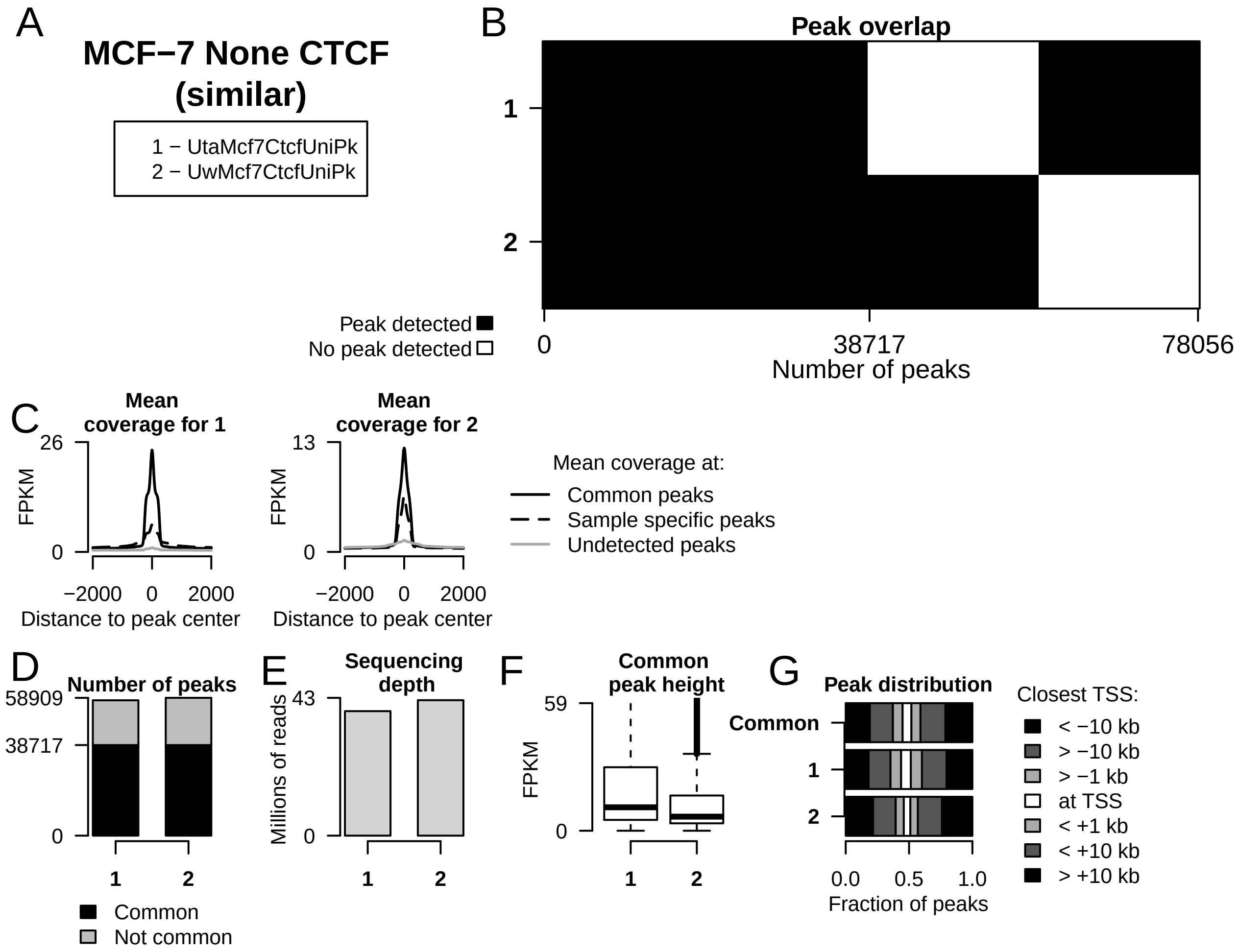

Supplementary figure 22: MCF−7 None CTCF (similar)

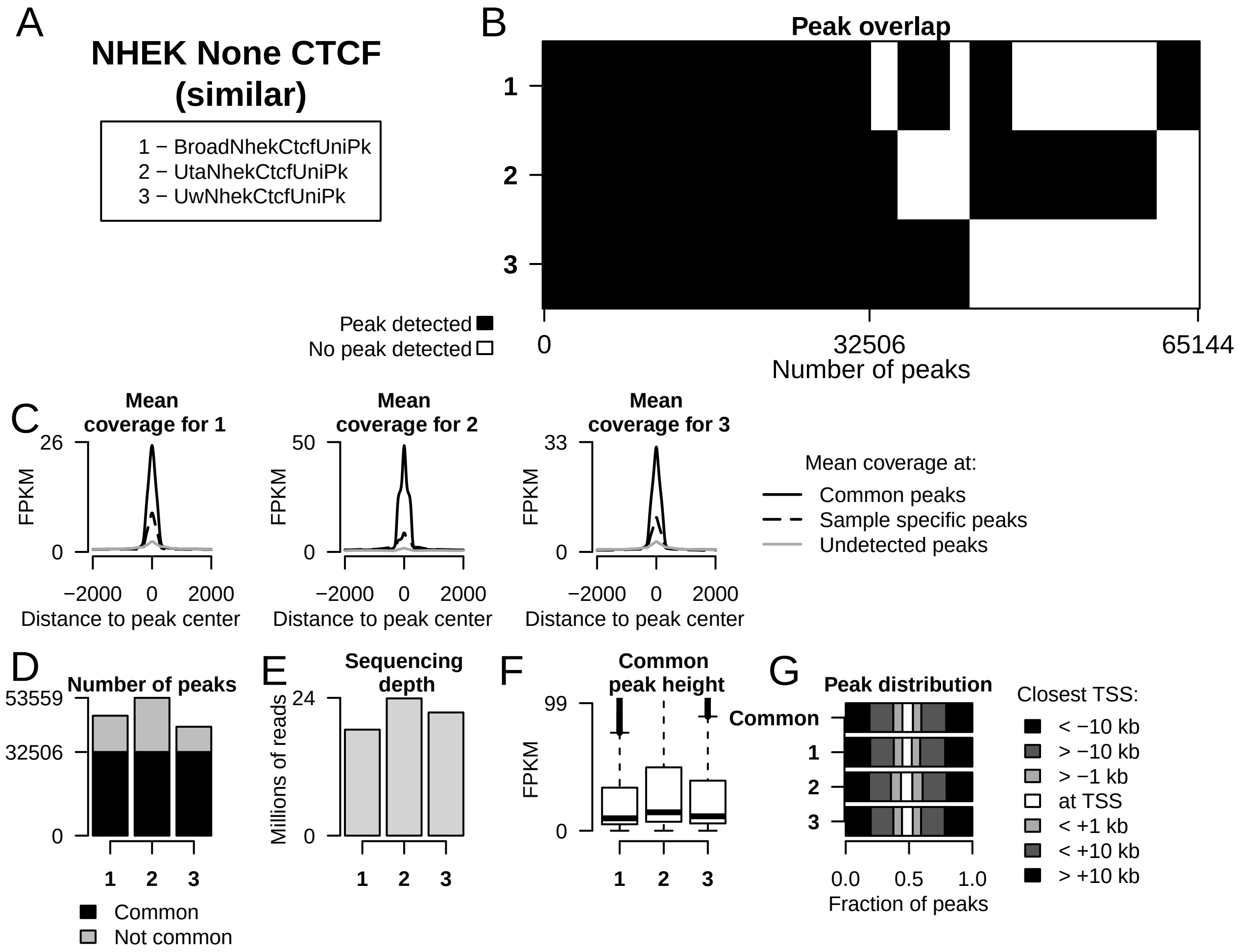

Supplementary figure 23: NHEK None CTCF (similar)

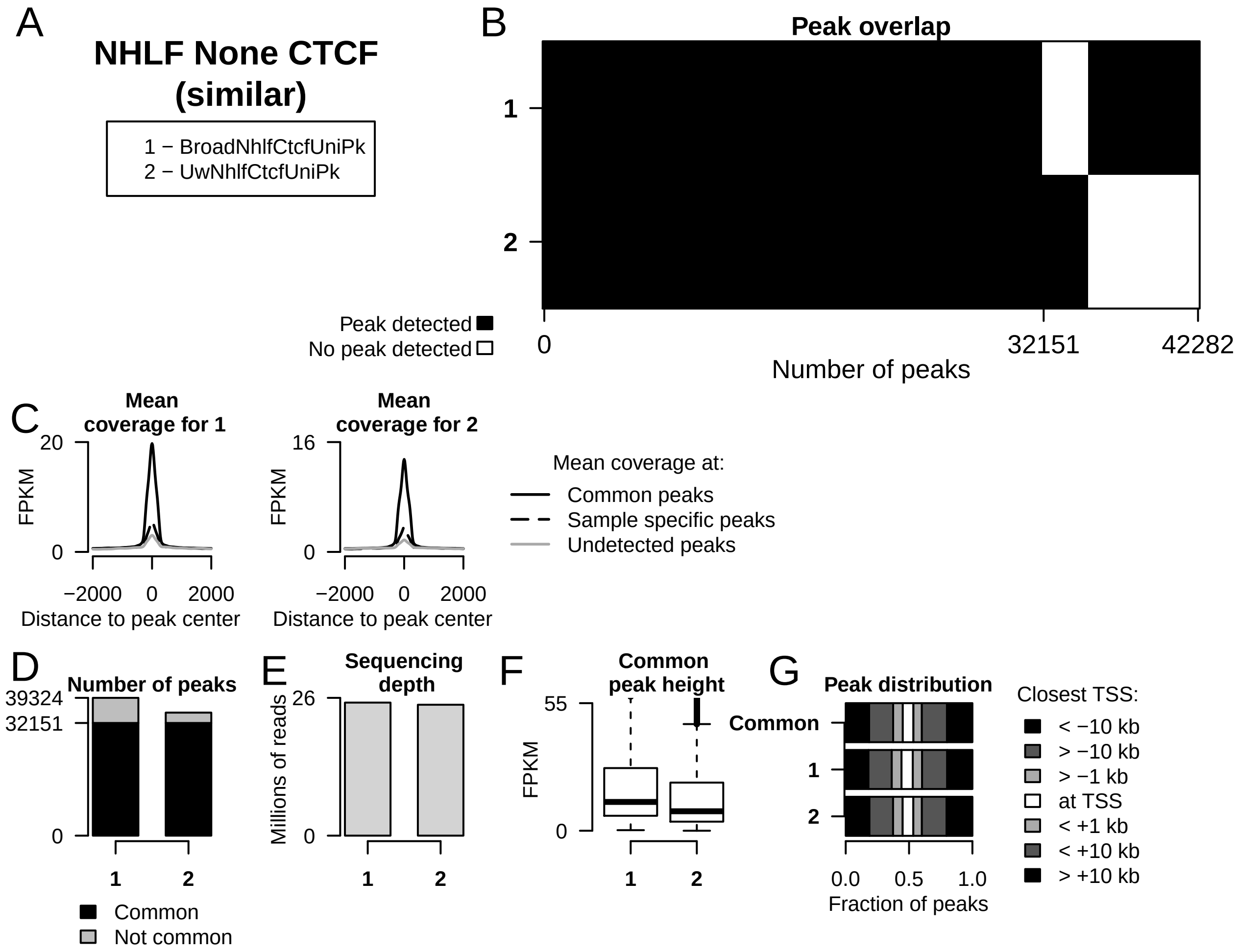

Supplementary figure 24: NHLF None CTCF (similar)

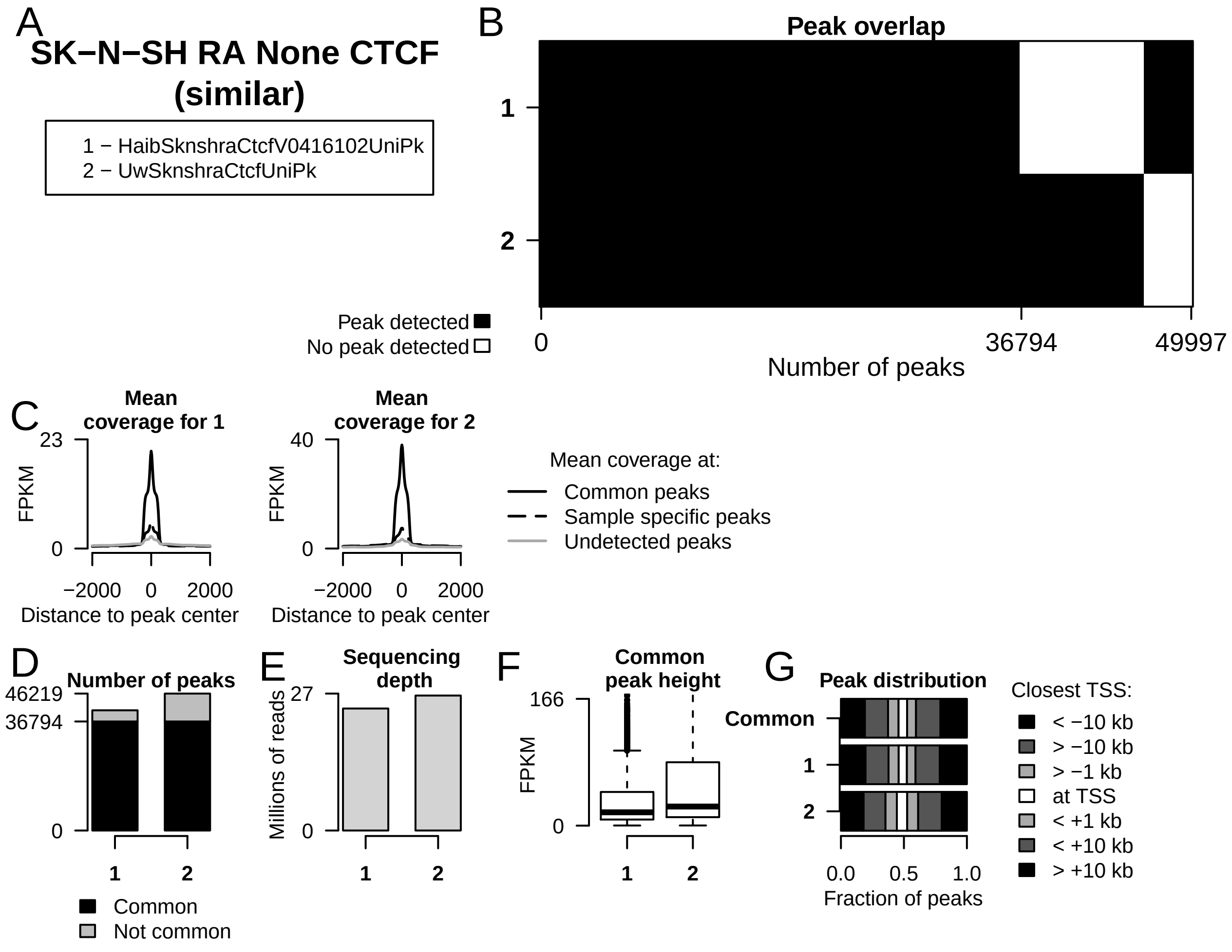

Supplementary figure 25: SK−N−SH RA None CTCF (similar)

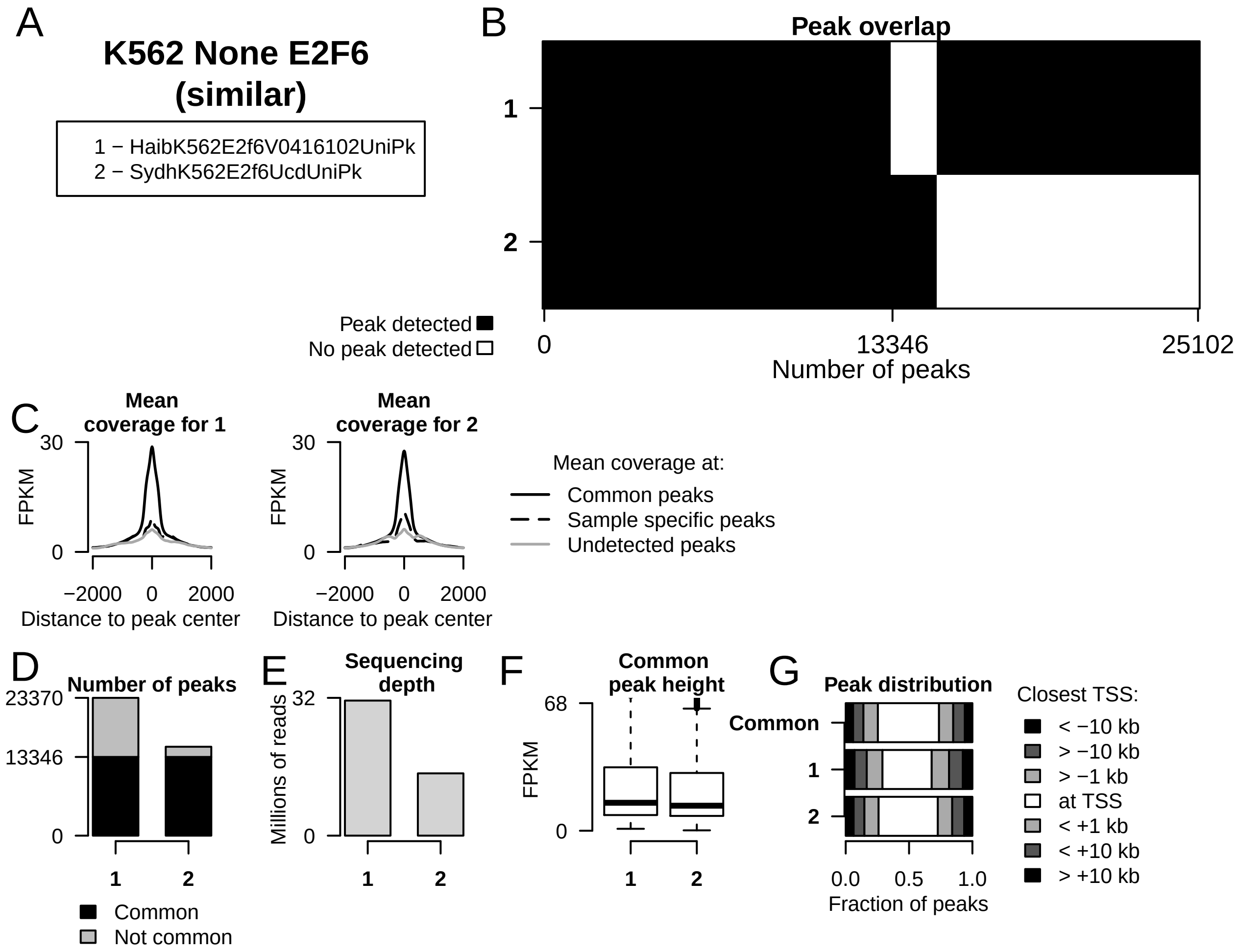

Supplementary figure 26: K562 None E2F6 (similar)

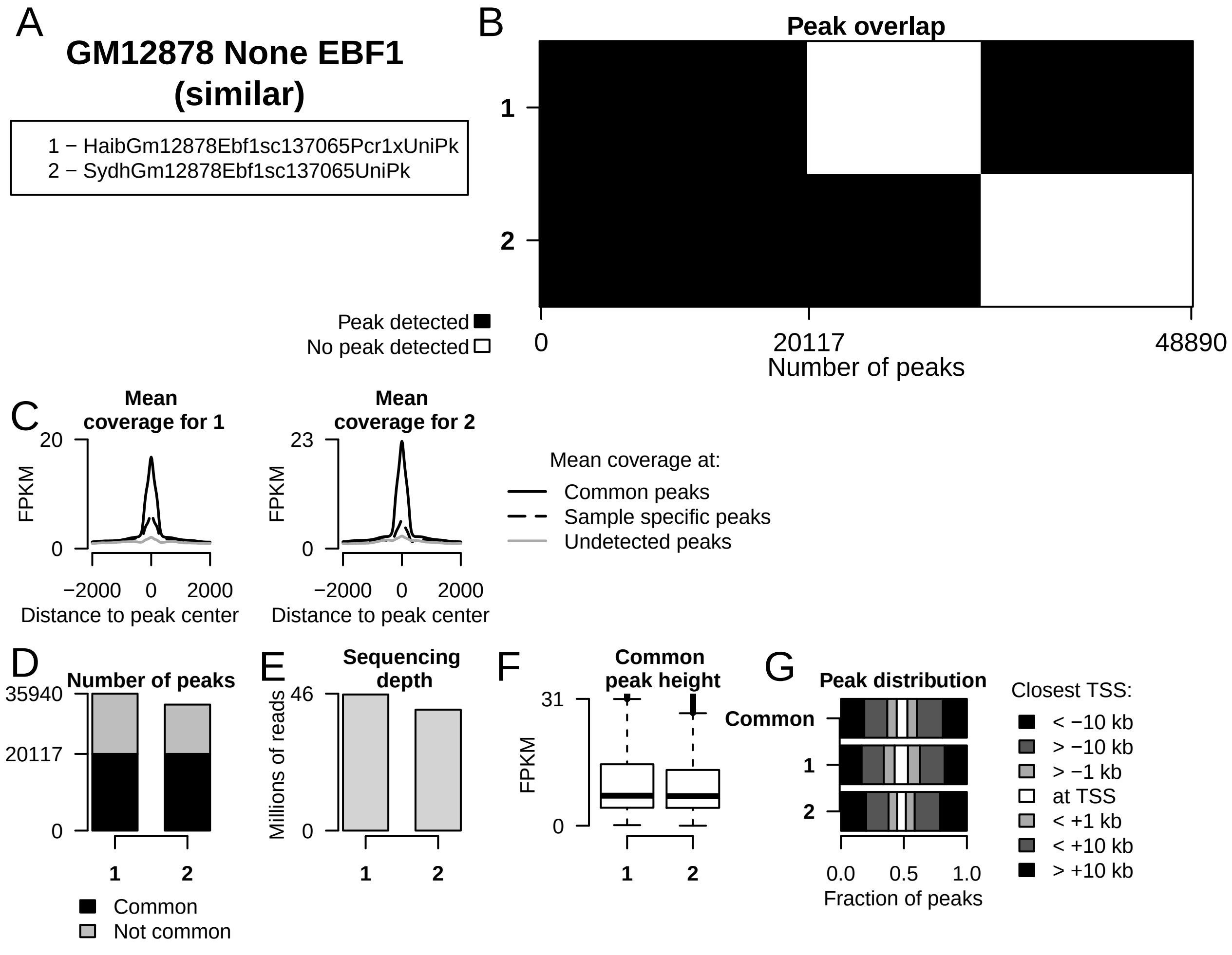

Supplementary figure 27: GM12878 None EBF1 (similar)

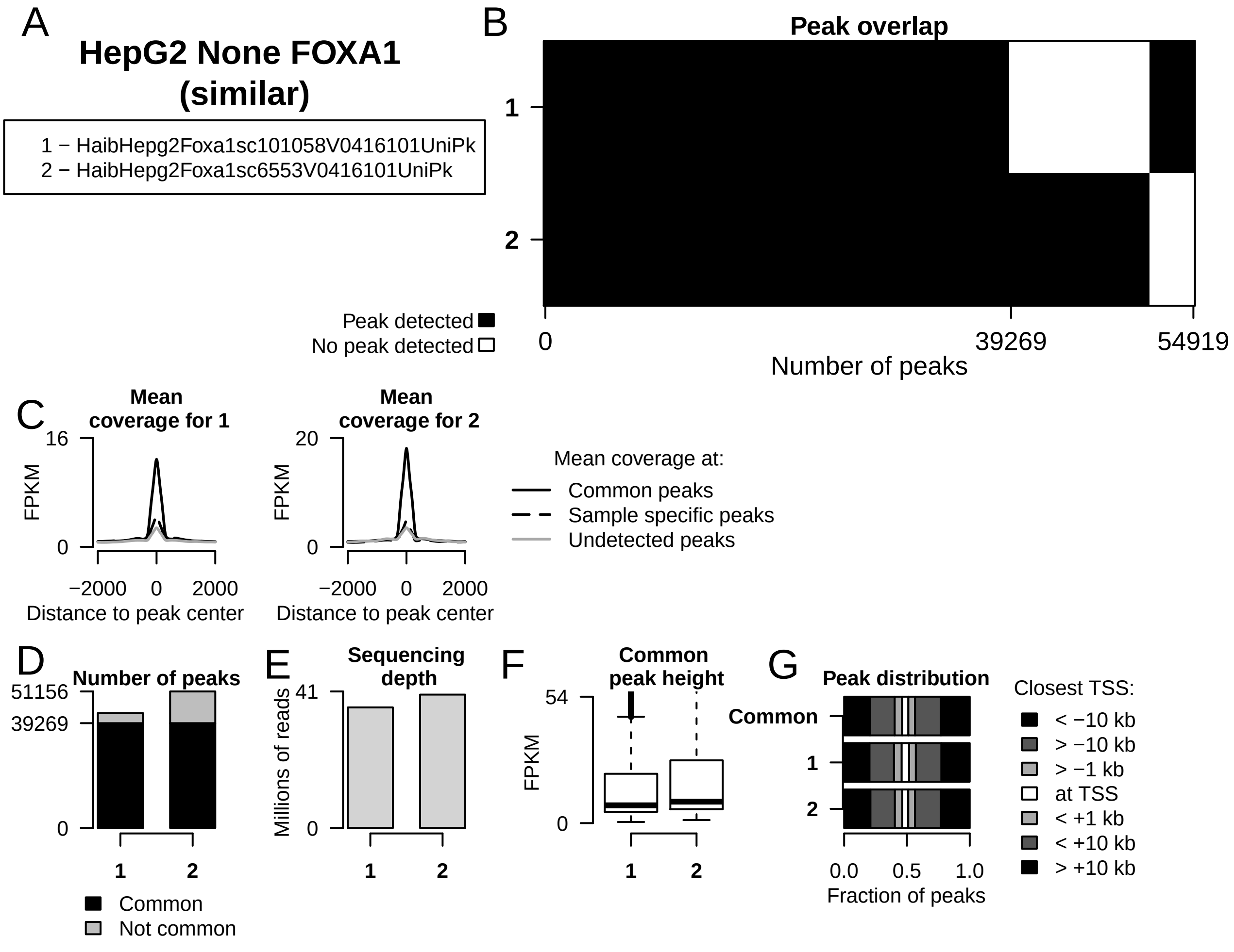

Supplementary figure 28: HepG2 None FOXA1 (similar)

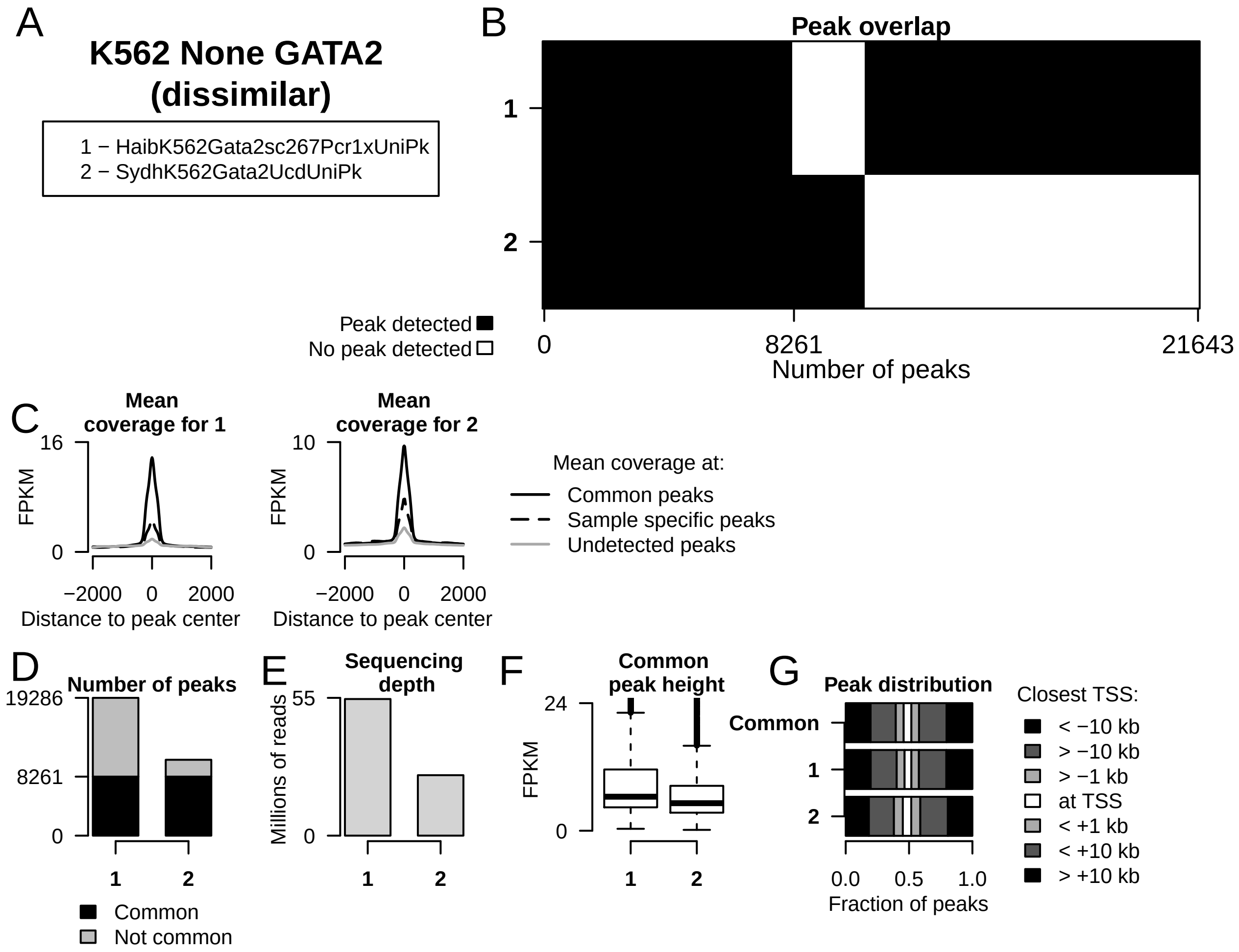

Supplementary figure 29: K562 None GATA2 (dissimilar)

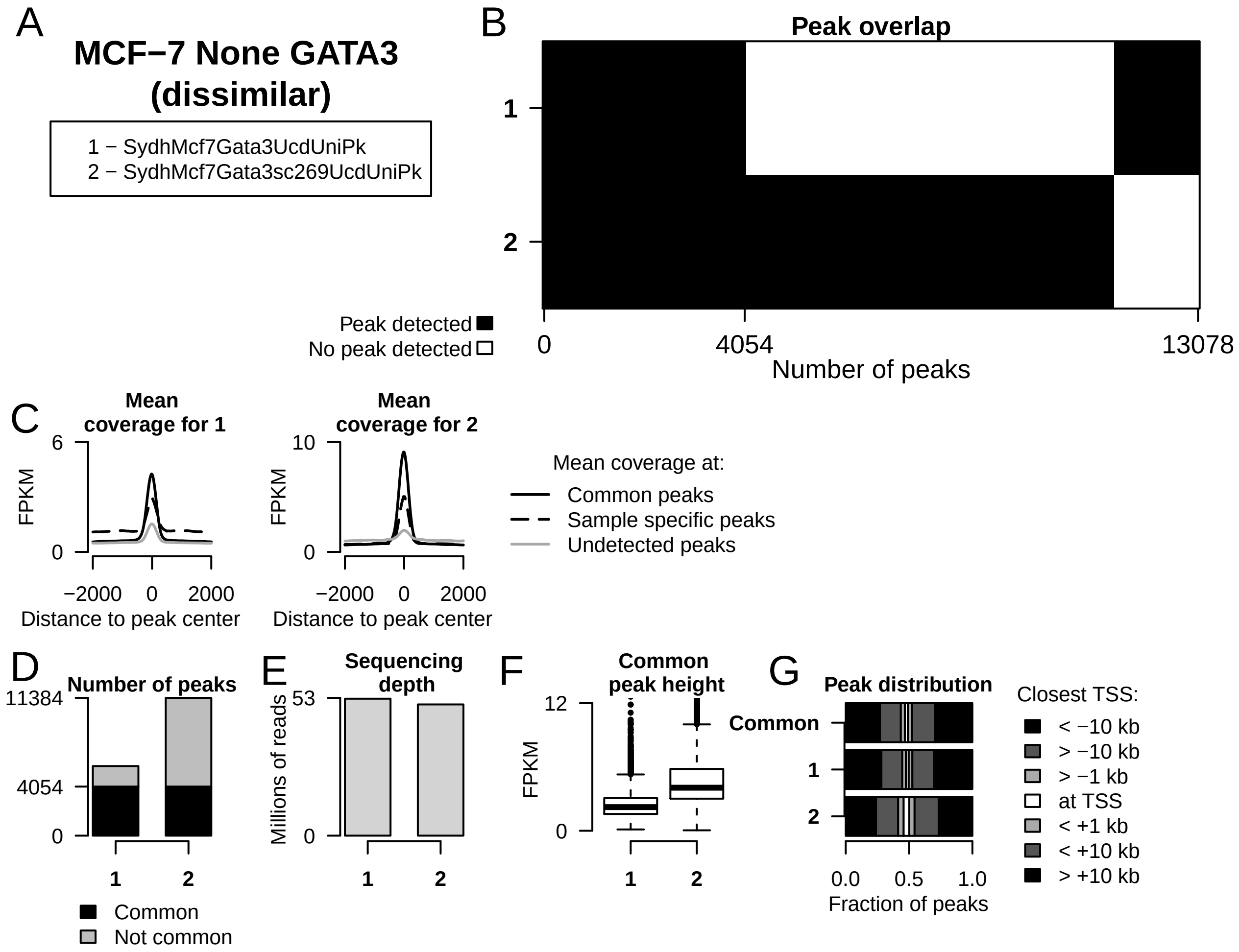

Supplementary figure 30: MCF−7 None GATA3 (dissimilar)

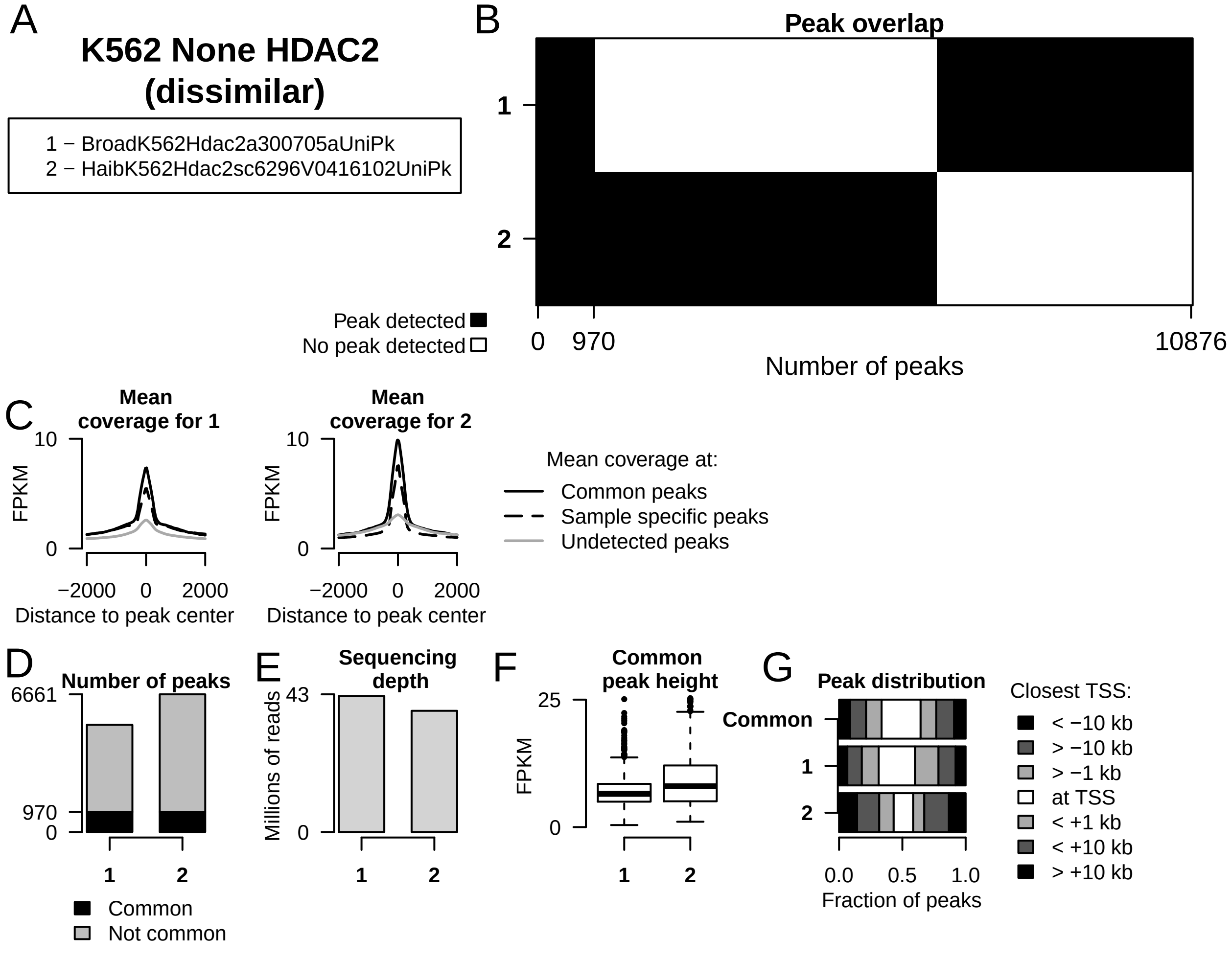

Supplementary figure 31: K562 None HDAC2 (dissimilar)

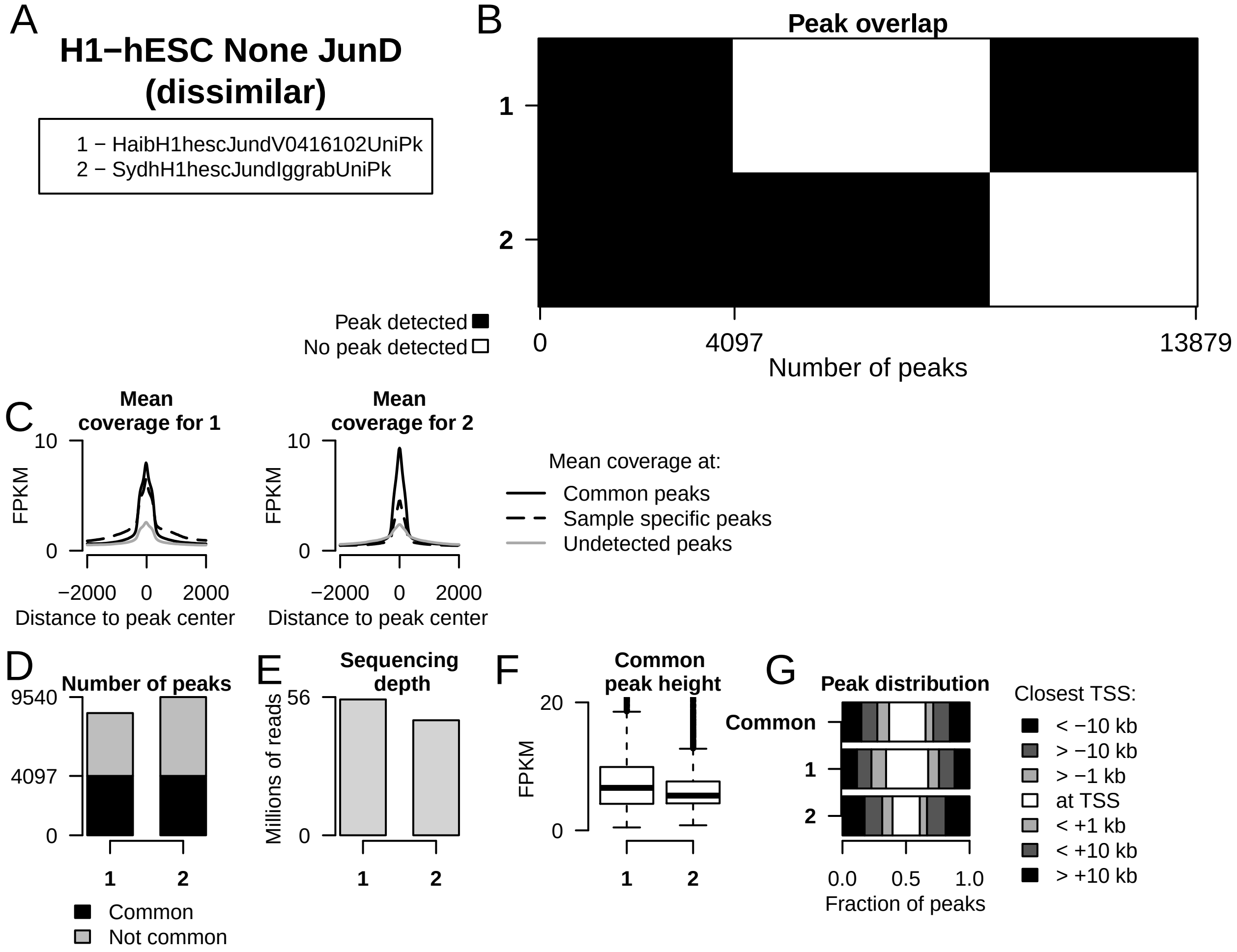

Supplementary figure 32: H1−hESC None JunD (dissimilar)

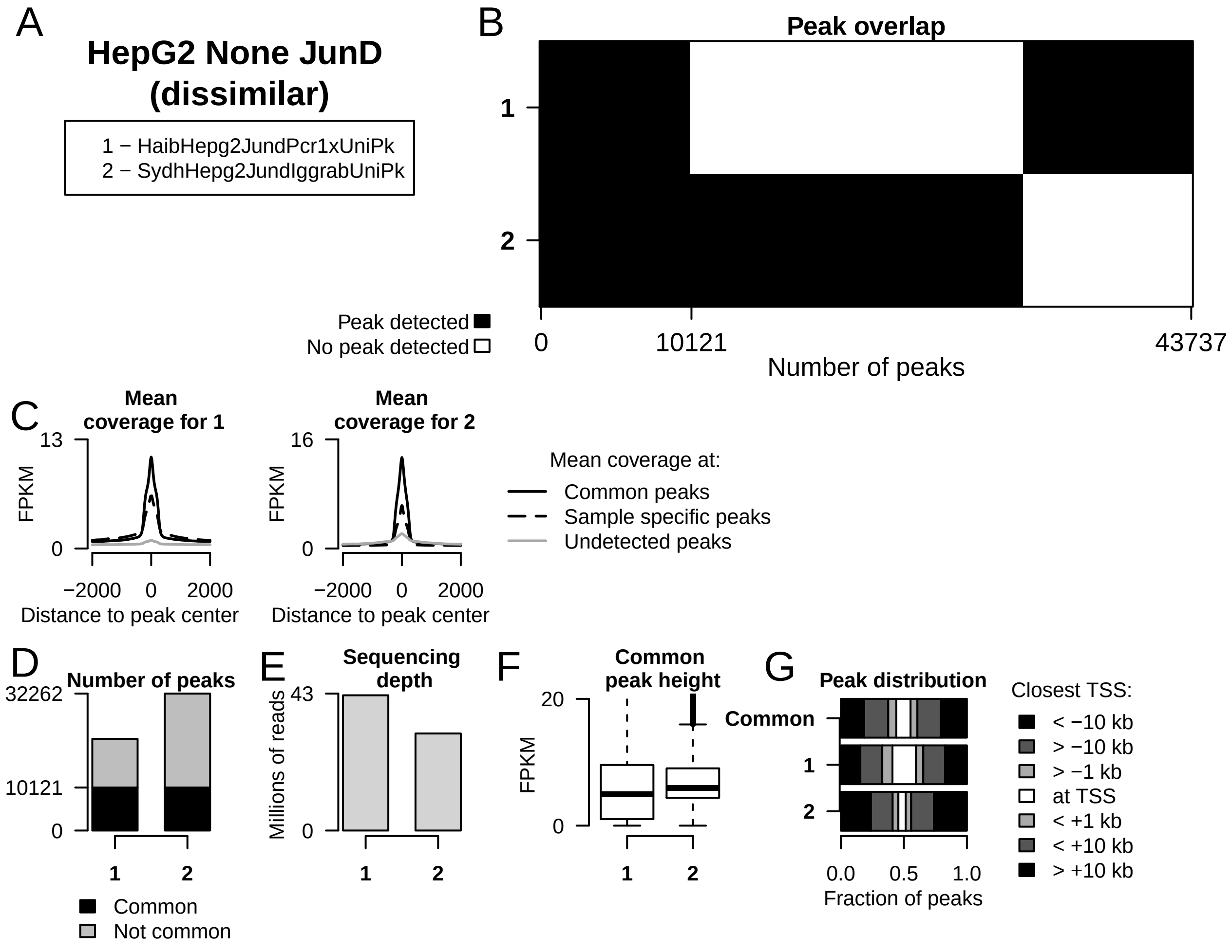

Supplementary figure 33: HepG2 None JunD (dissimilar)

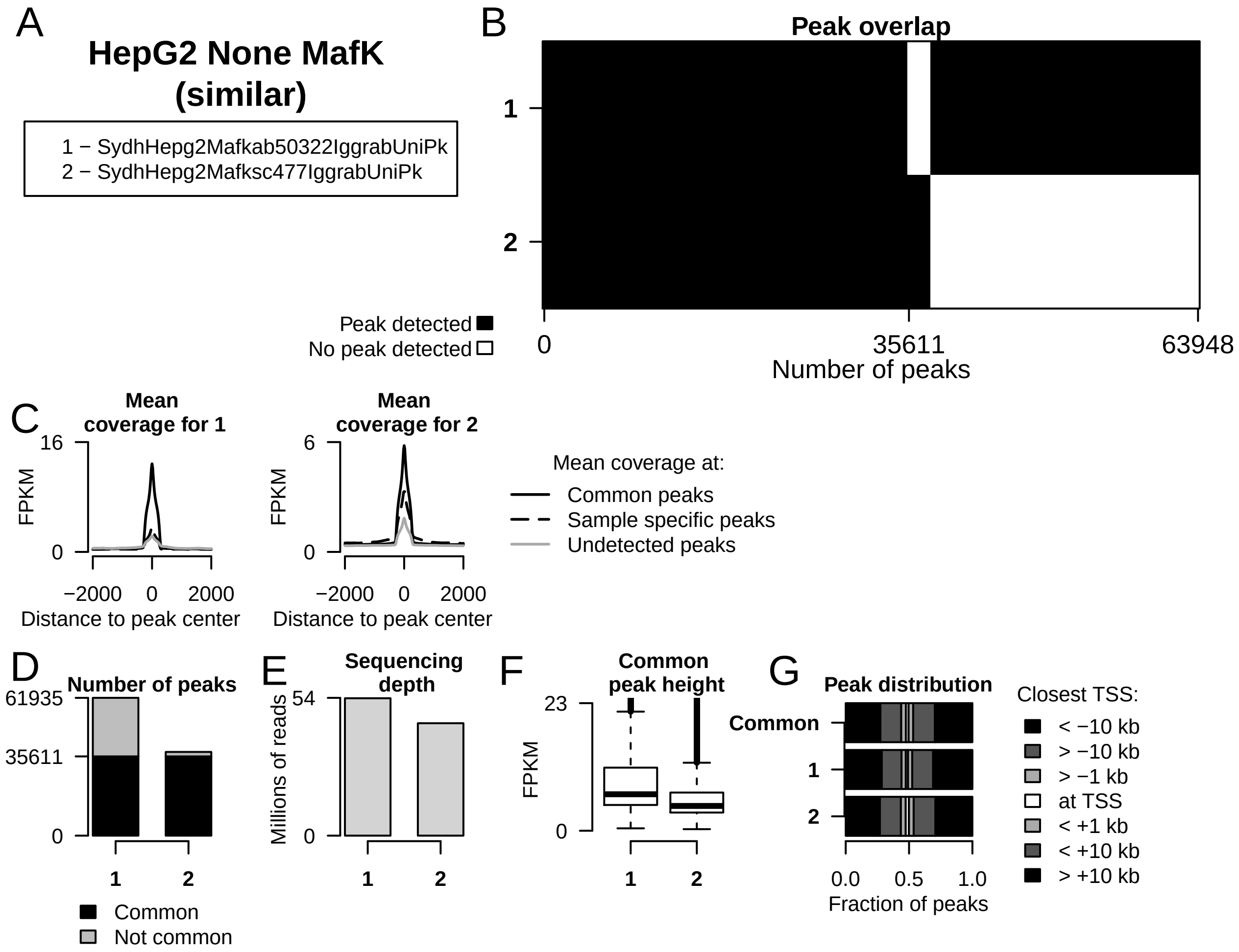

Supplementary figure 34: HepG2 None MafK (similar)

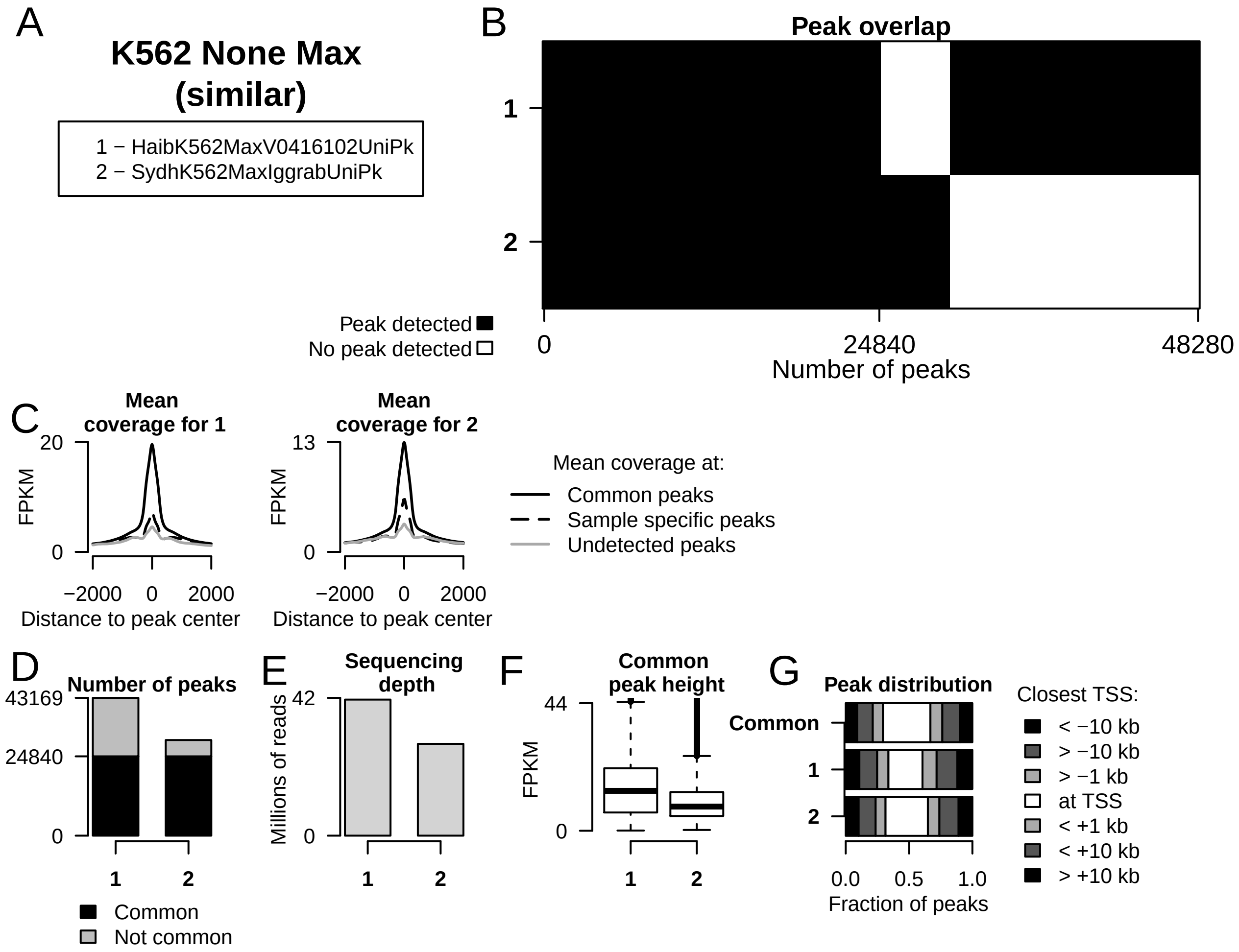

Supplementary figure 35: K562 None Max (similar)

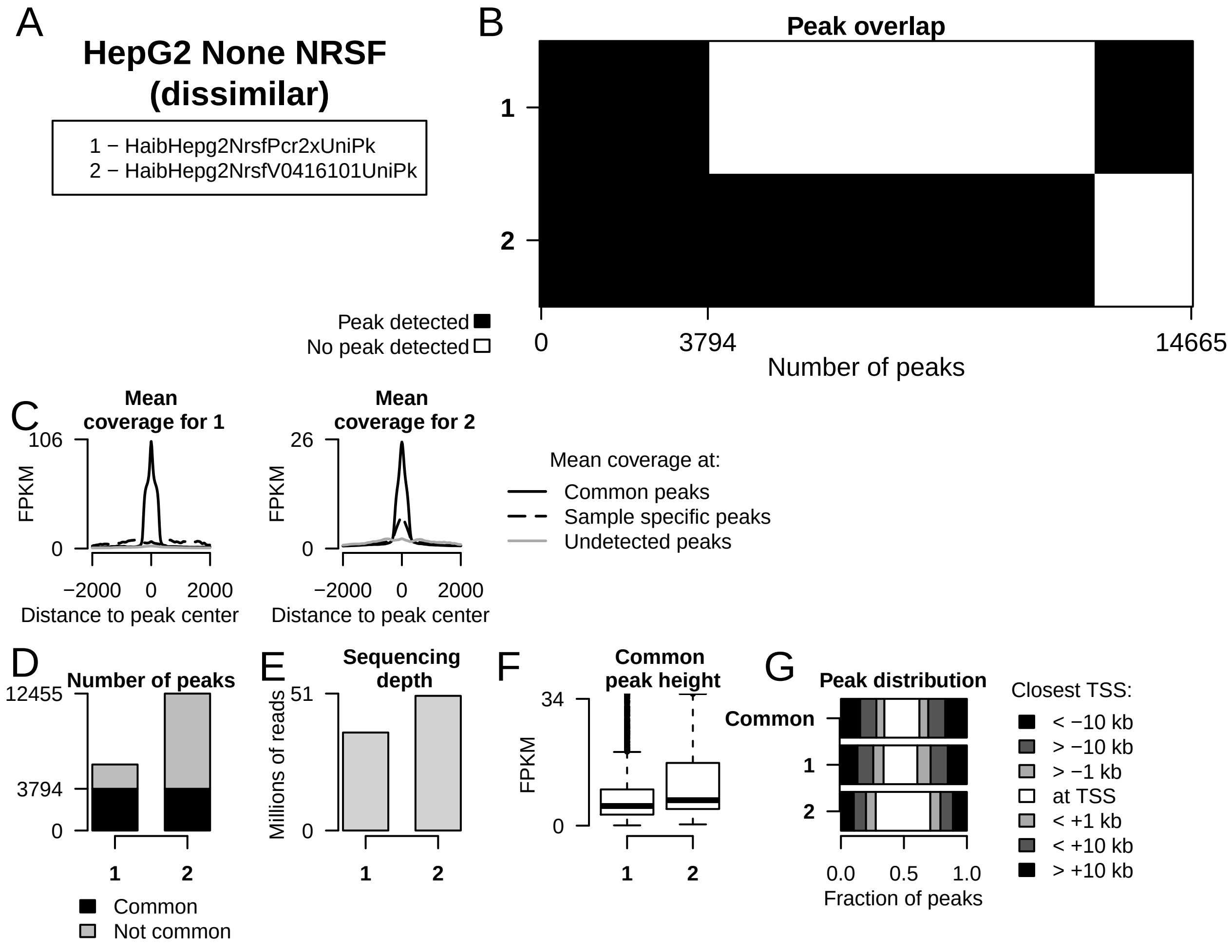

Supplementary figure 36: HepG2 None NRSF (dissimilar)

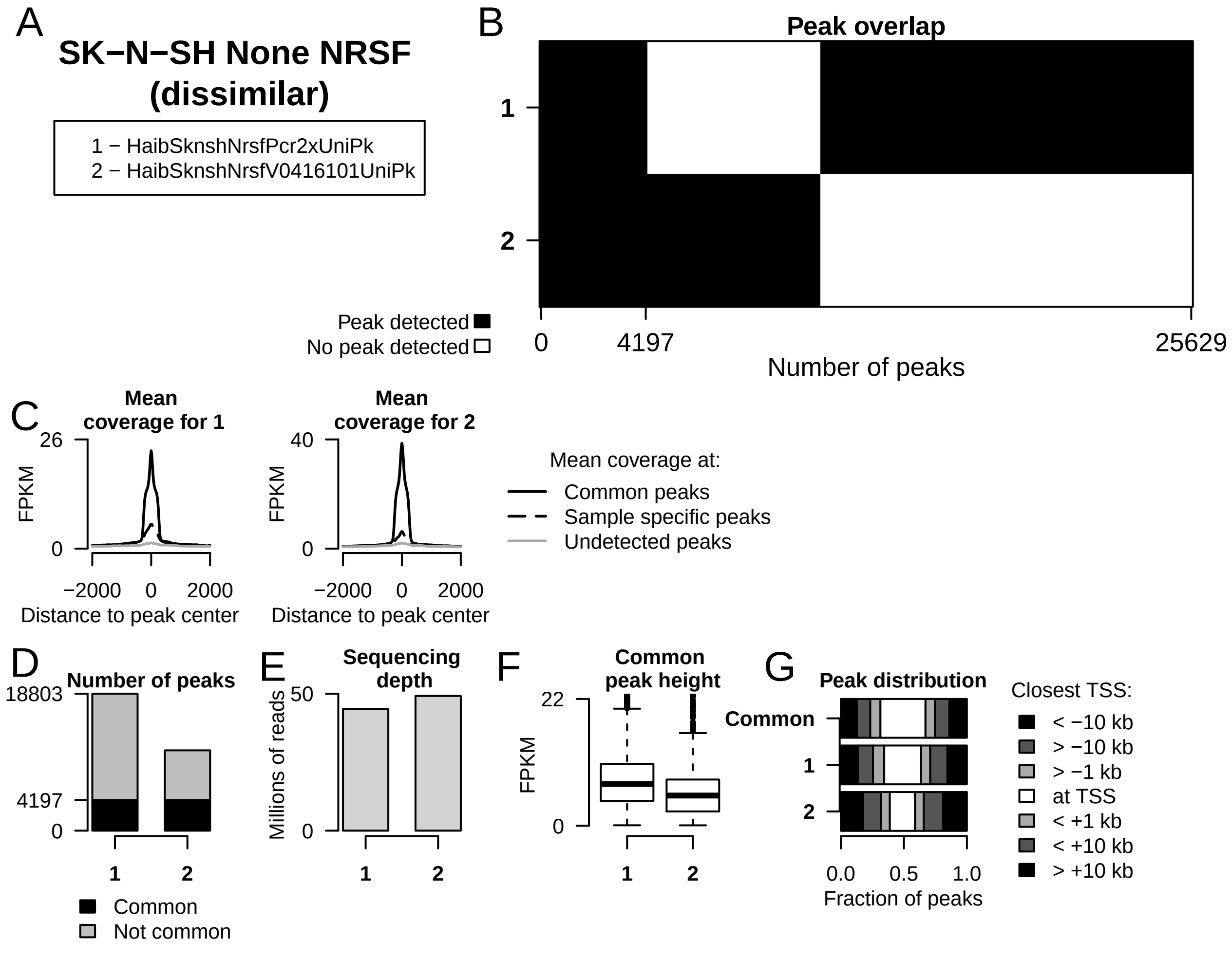

Supplementary figure 37: SK−N−SH None NRSF (dissimilar)

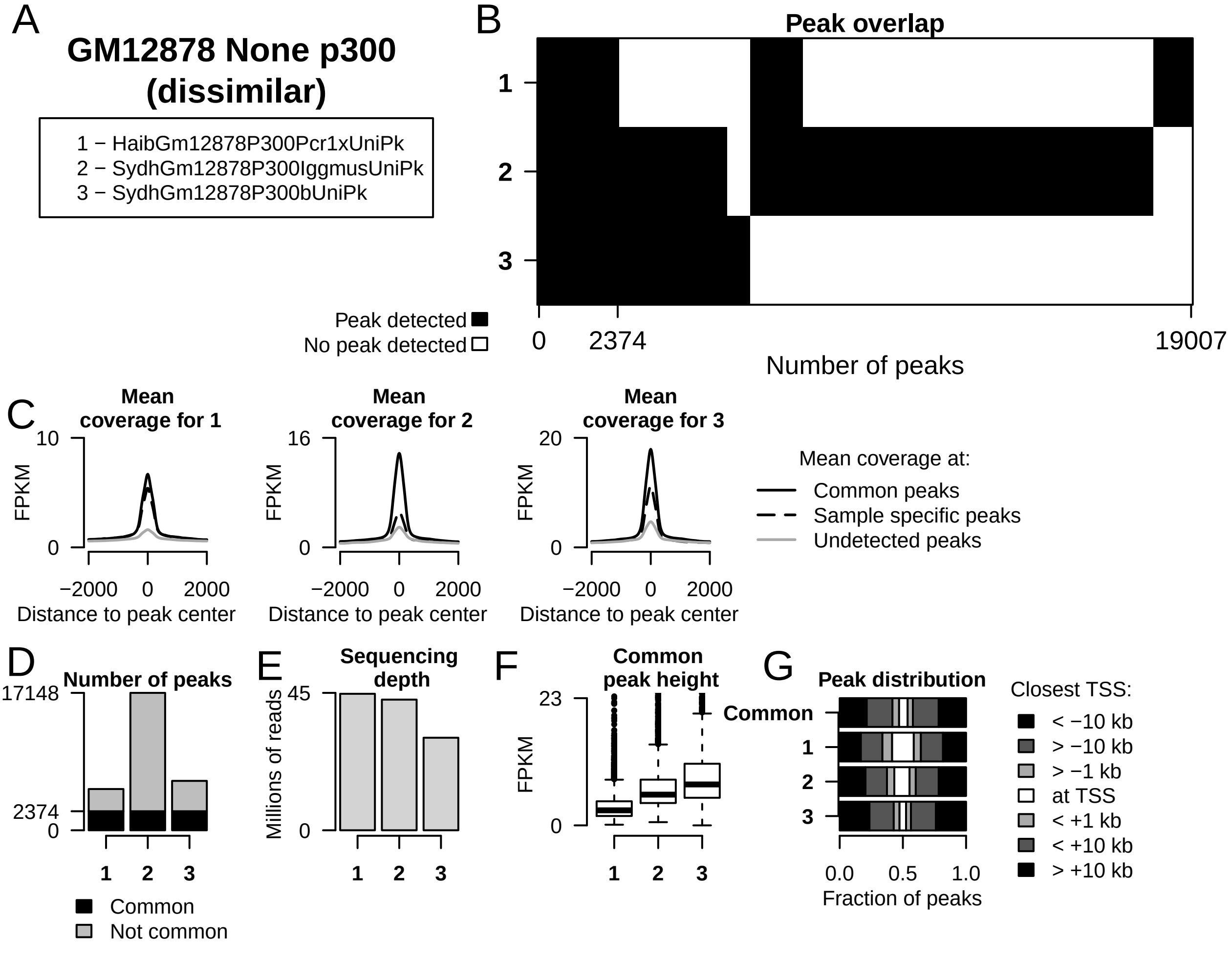

Supplementary figure 38: GM12878 None p300 (dissimilar)

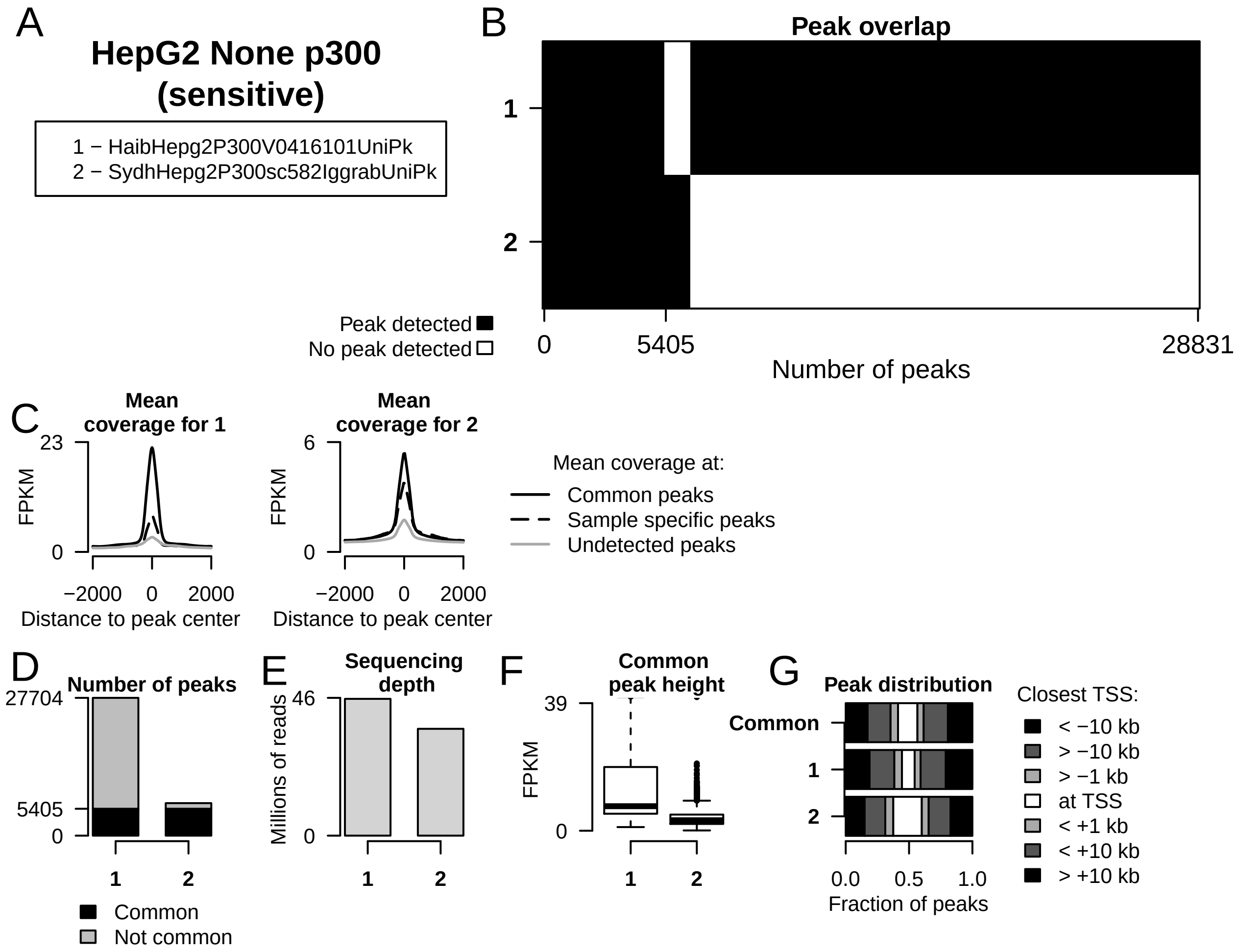

Supplementary figure 39: HepG2 None p300 (sensitive)

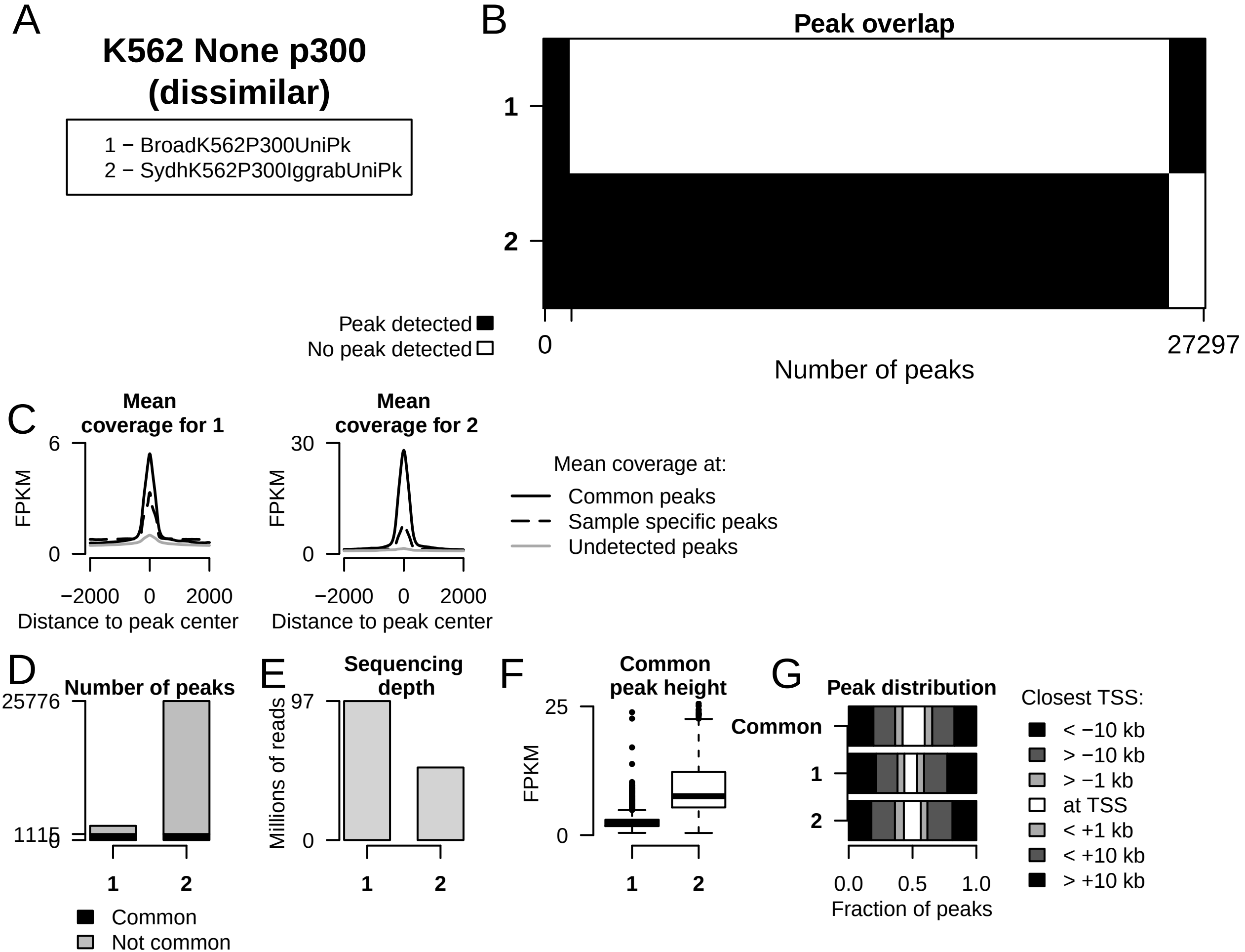

Supplementary figure 40: K562 None p300 (dissimilar)

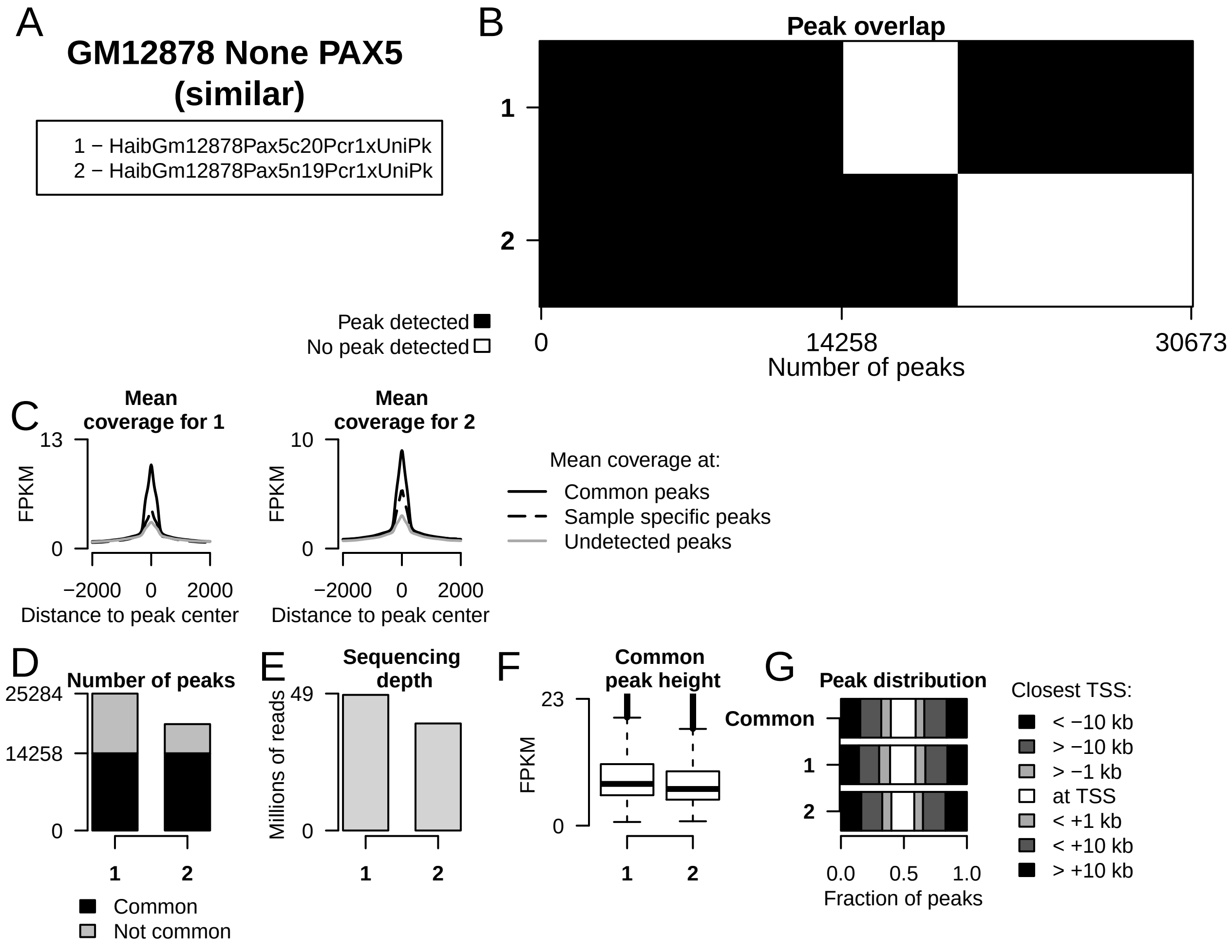

Supplementary figure 41: GM12878 None PAX5 (similar)

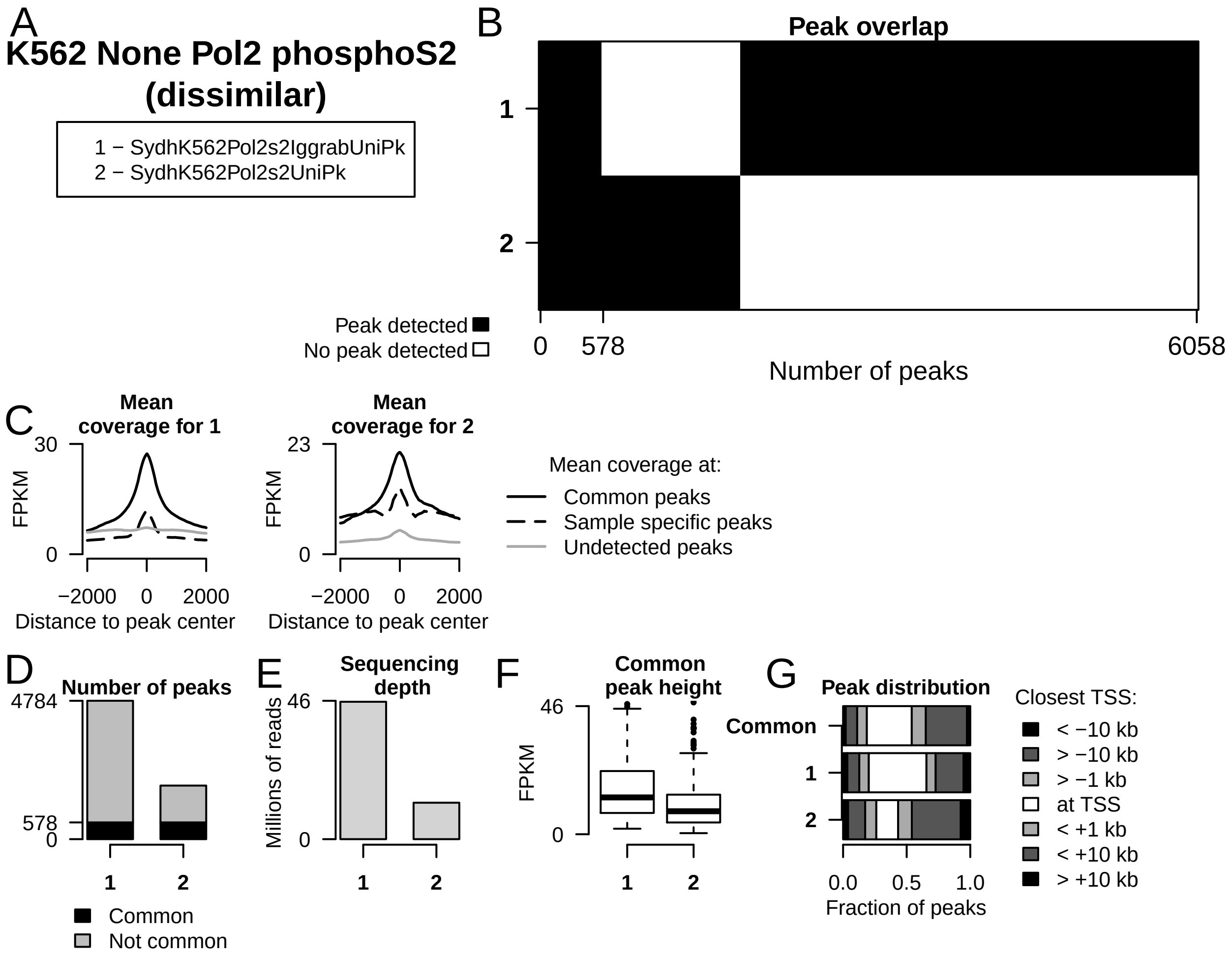

Supplementary figure 42: K562 None Pol2 phosphoS2 (dissimilar)

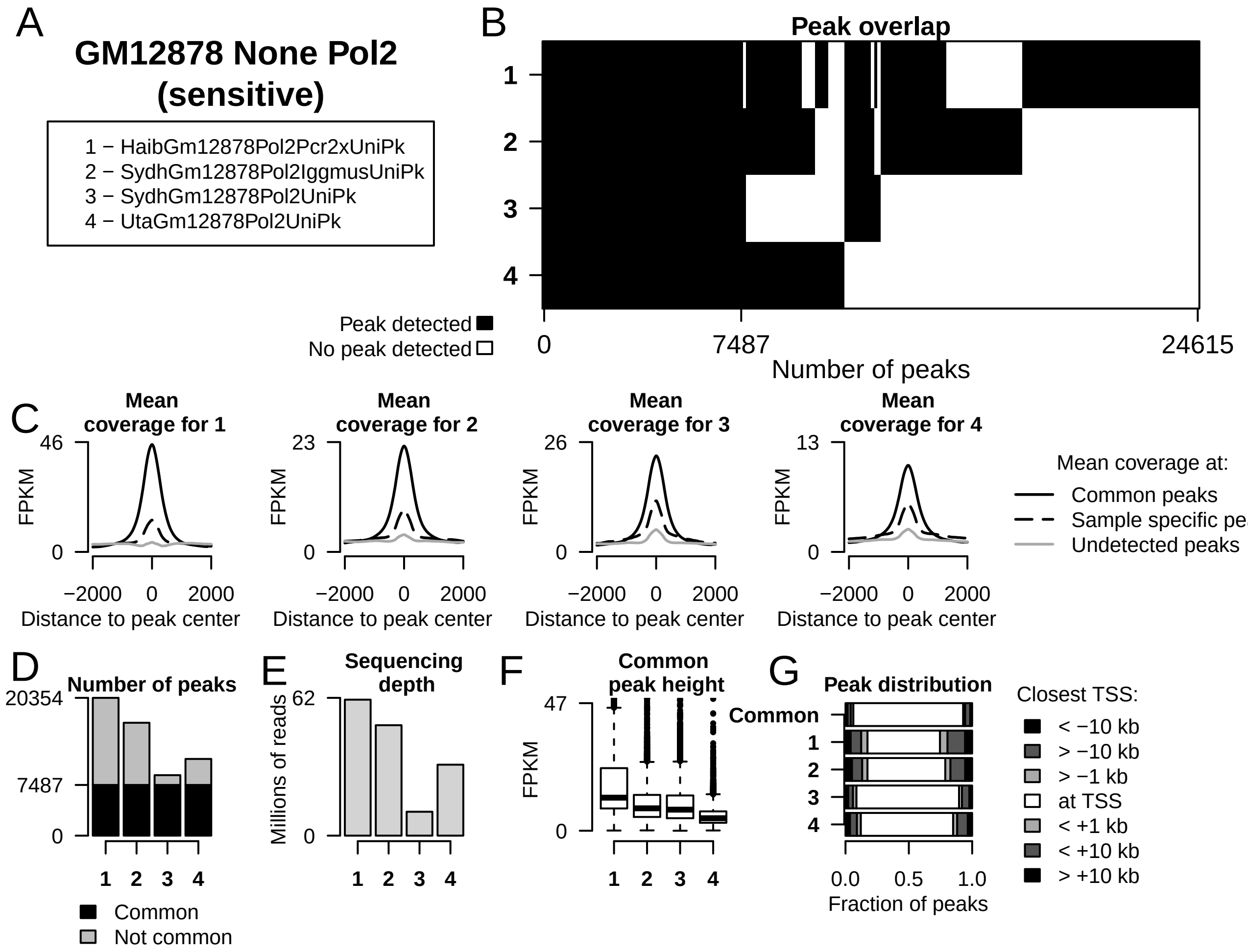

Supplementary figure 43: GM12878 None Pol2 (sensitive)

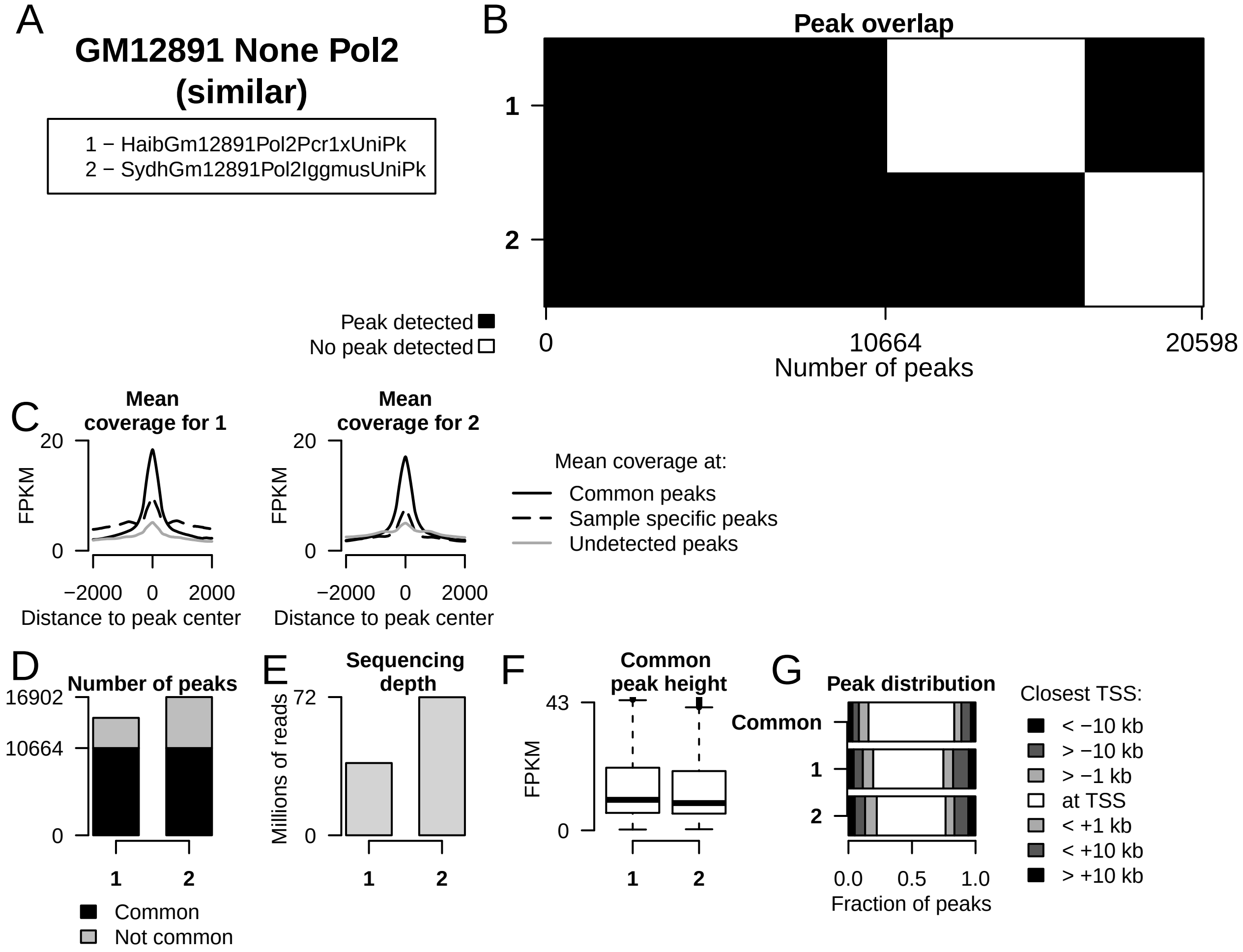

Supplementary figure 44: GM12891 None Pol2 (similar)

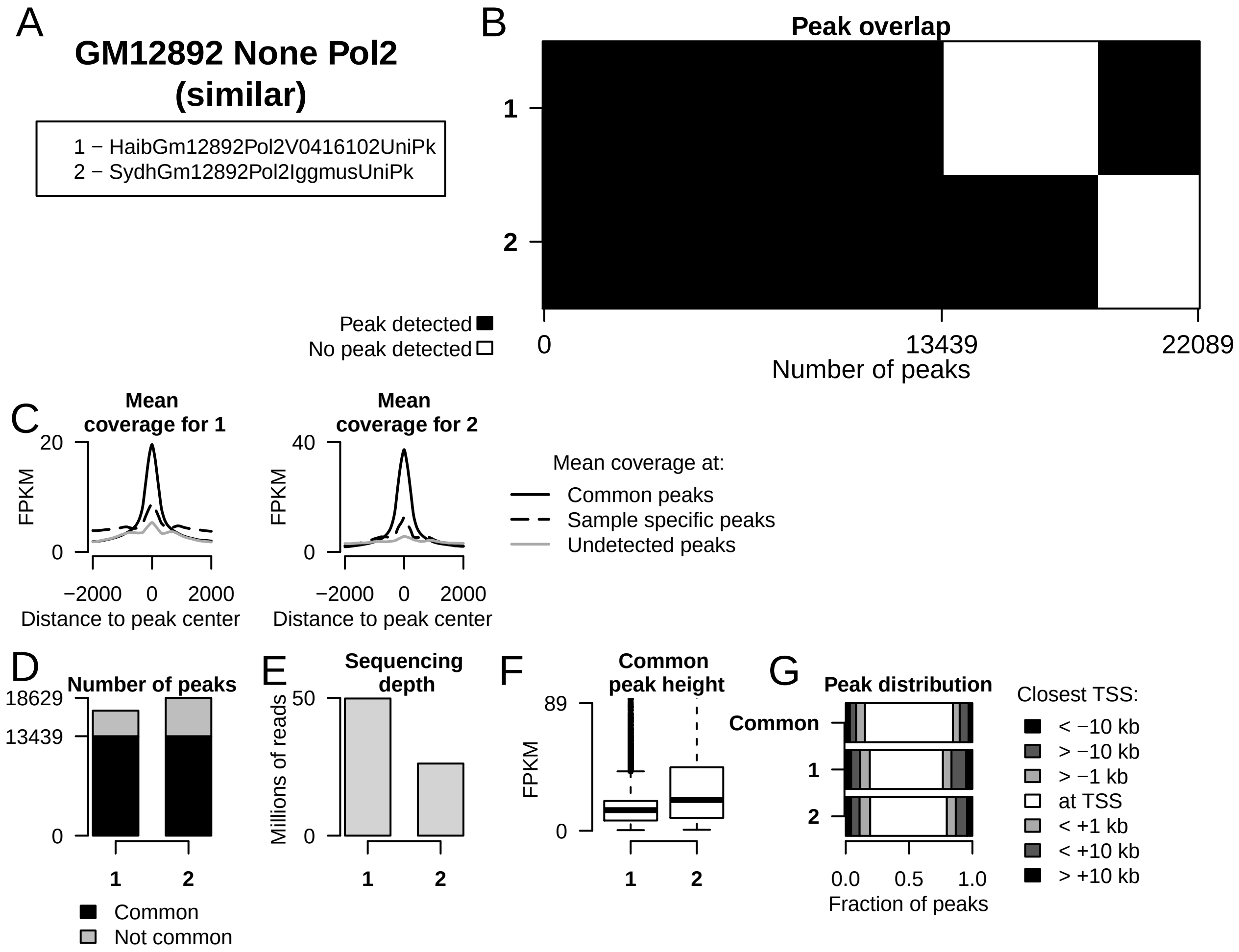

Supplementary figure 45: GM12892 None Pol2 (similar)

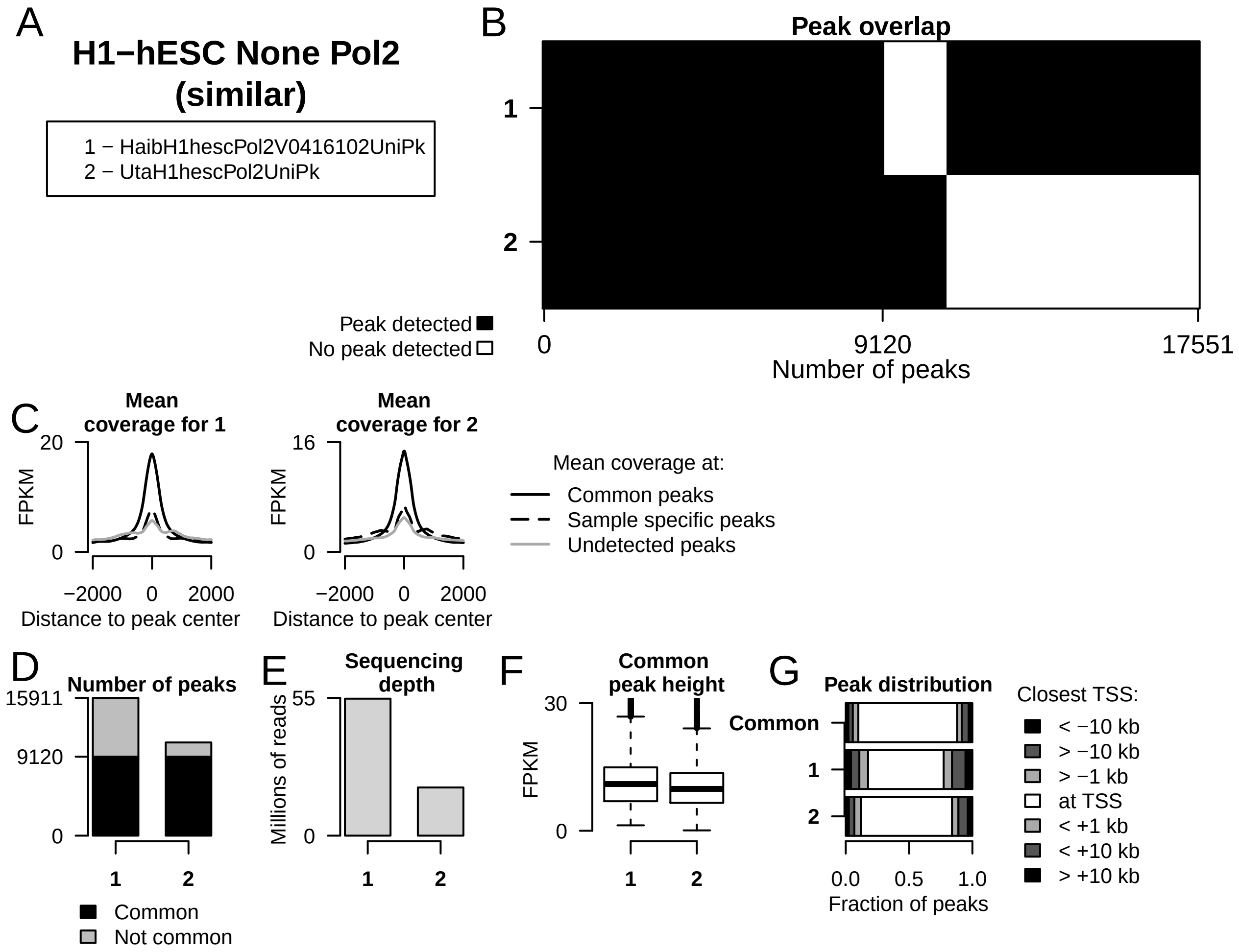

Supplementary figure 46: H1−hESC None Pol2 (similar)

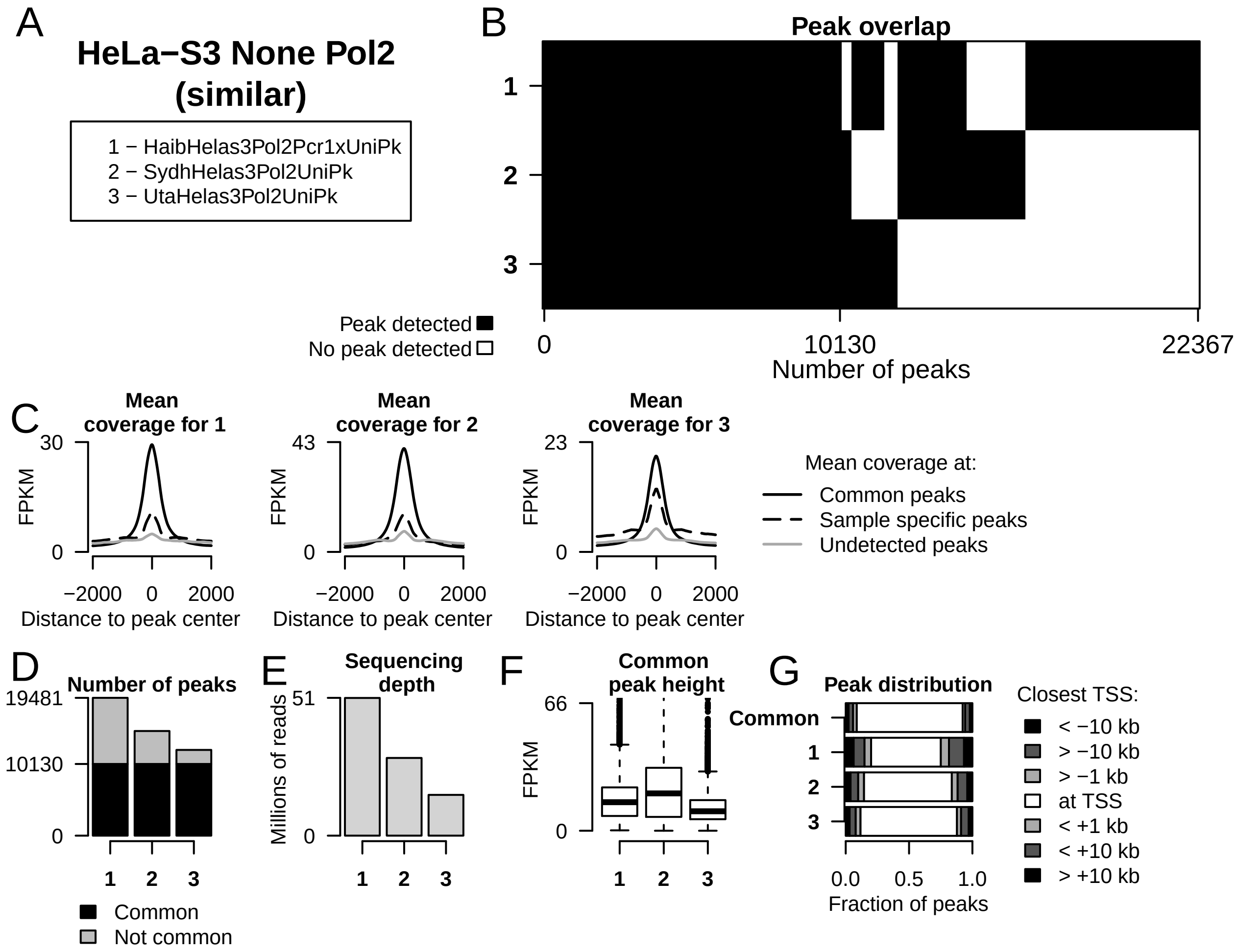

Supplementary figure 47: HeLa−S3 None Pol2 (similar)

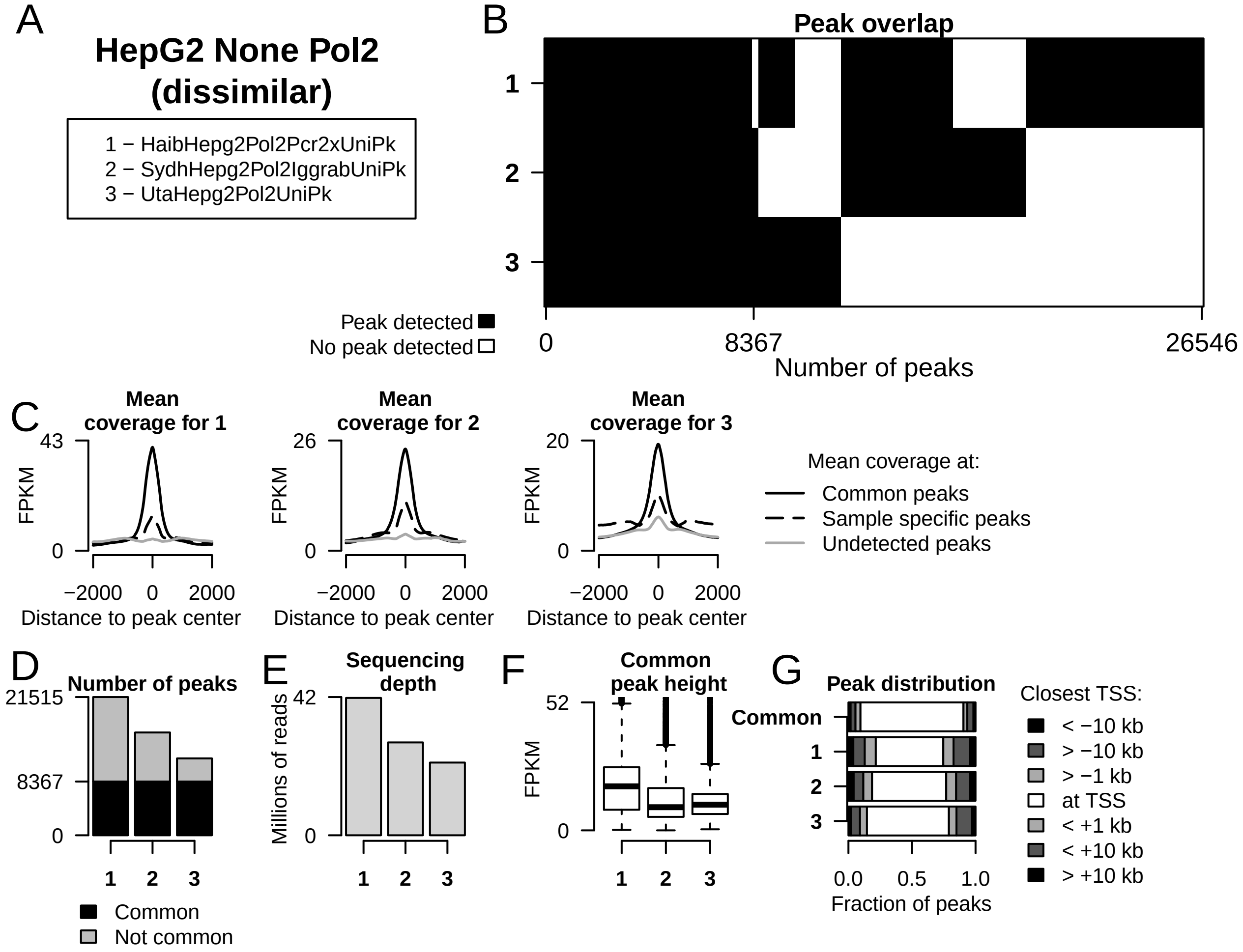

Supplementary figure 48: HepG2 None Pol2 (dissimilar)

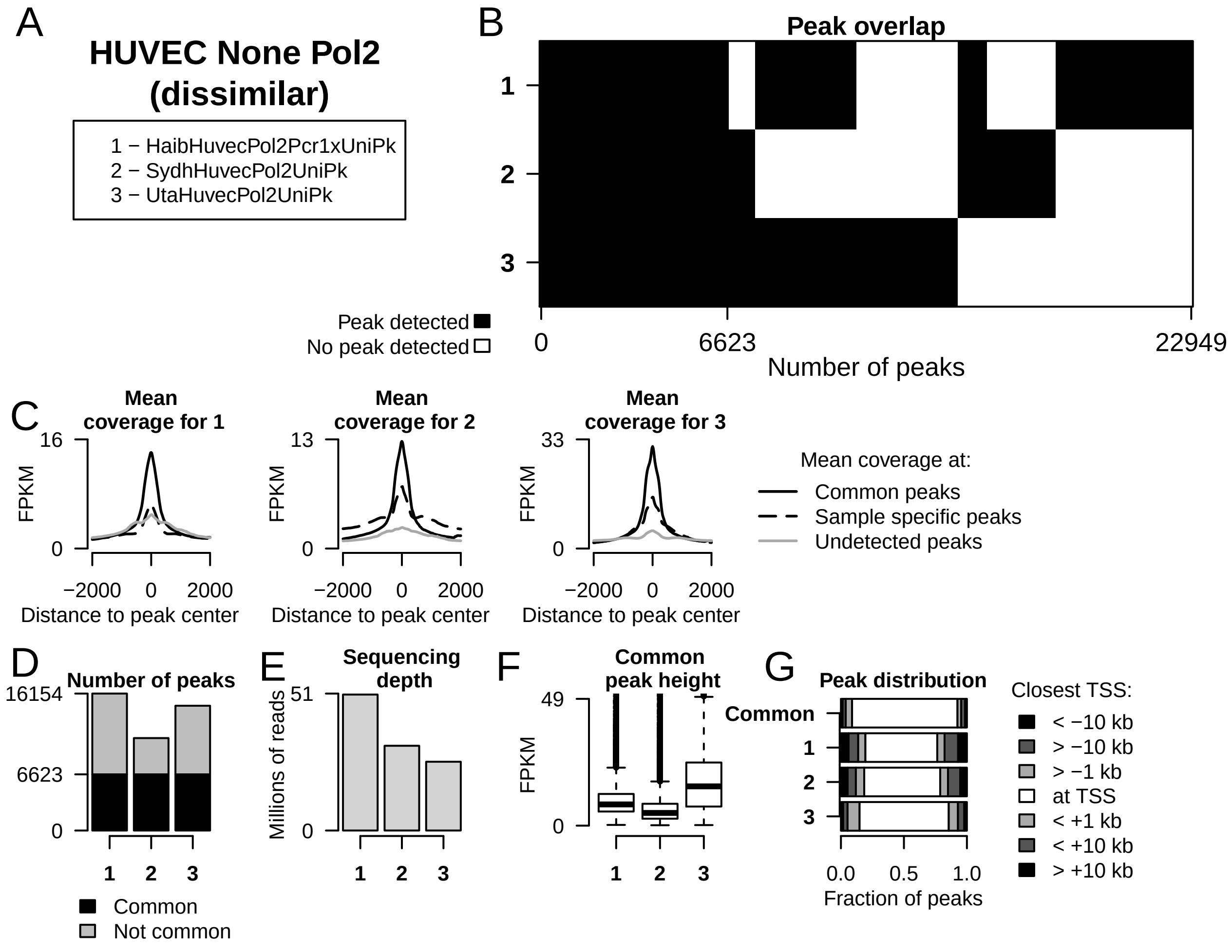

Supplementary figure 49: HUVEC None Pol2 (dissimilar)

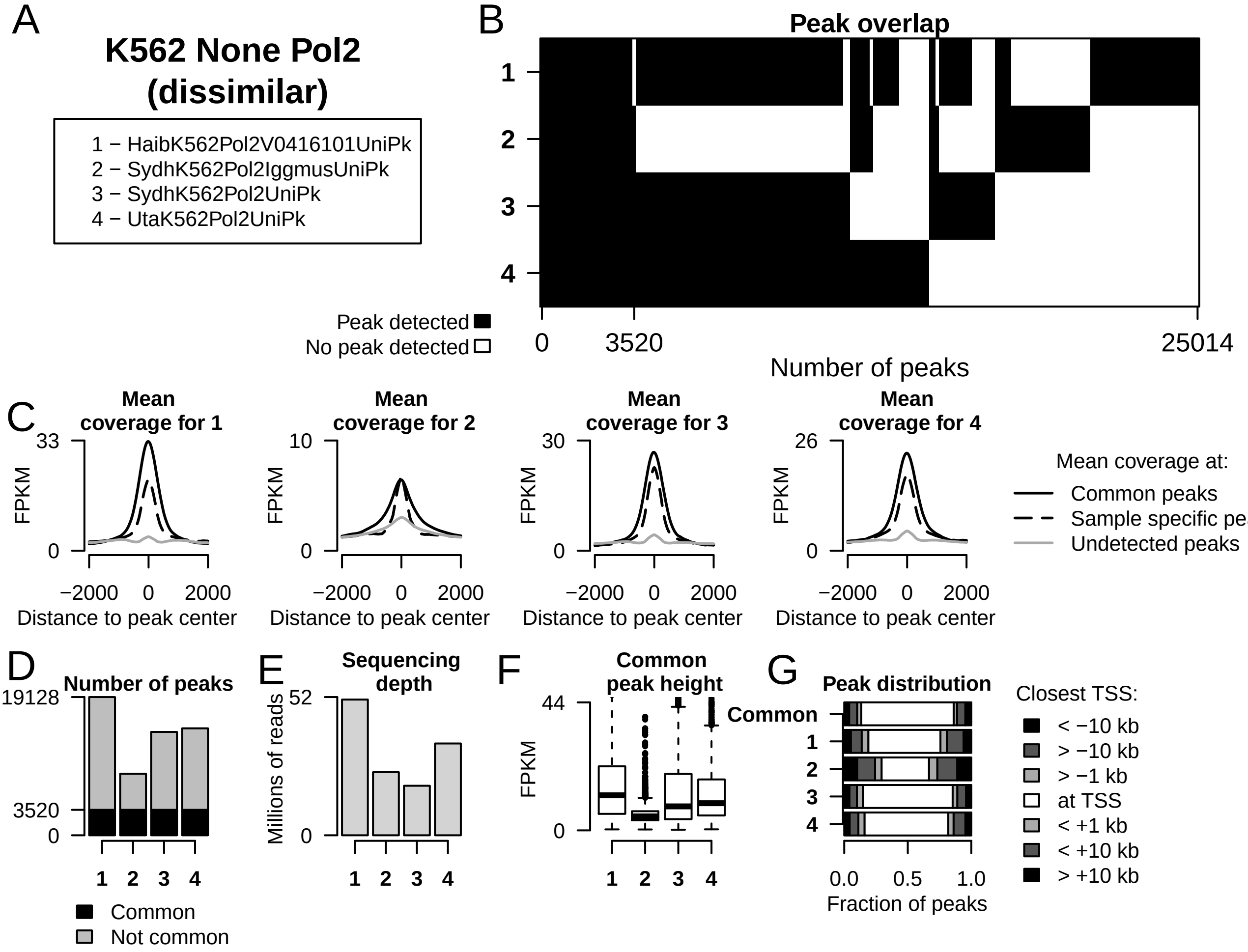

Supplementary figure 50: K562 None Pol2 (dissimilar)

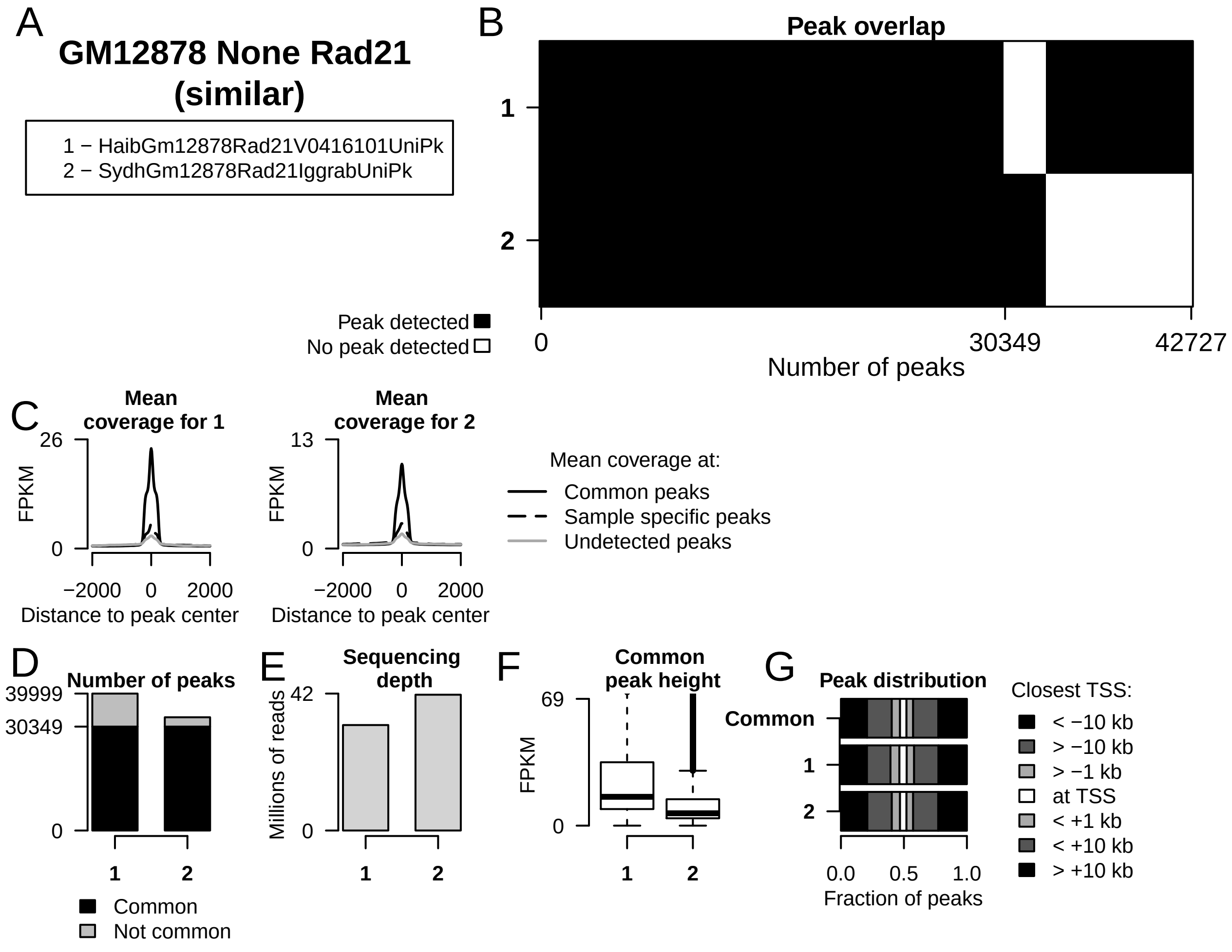

Supplementary figure 51: GM12878 None Rad21 (similar)

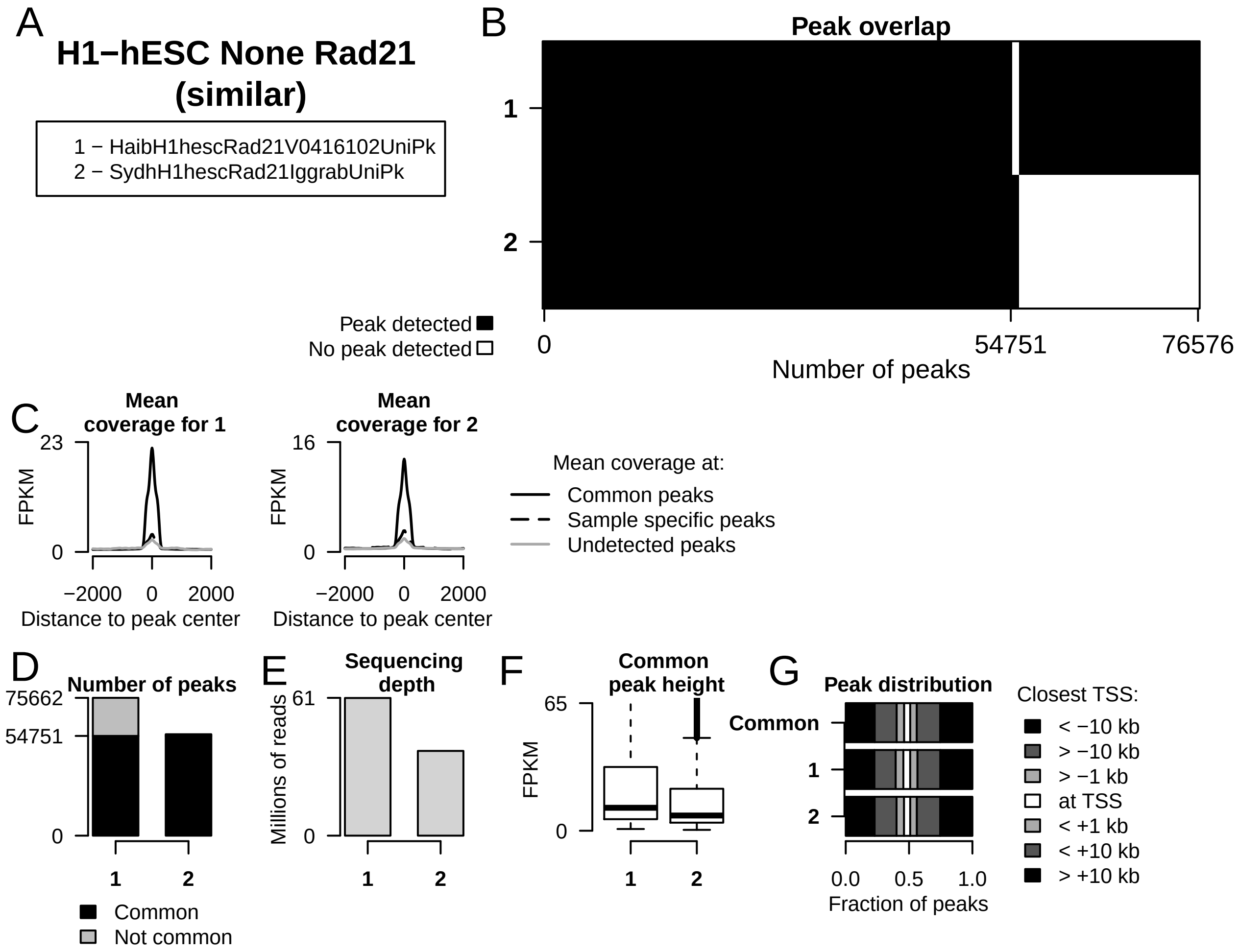

Supplementary figure 52: H1−hESC None Rad21 (similar)

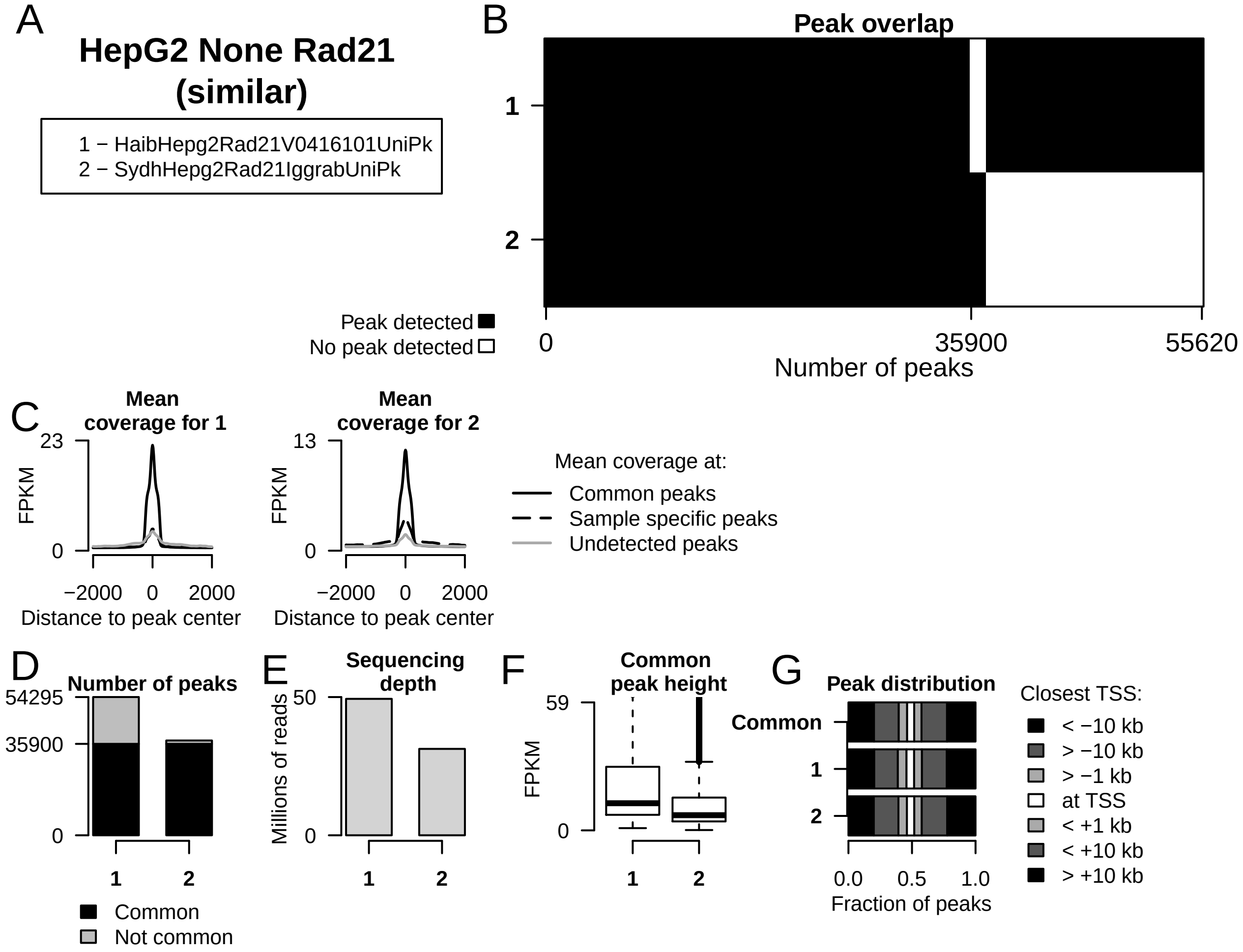

Supplementary figure 53: HepG2 None Rad21 (similar)

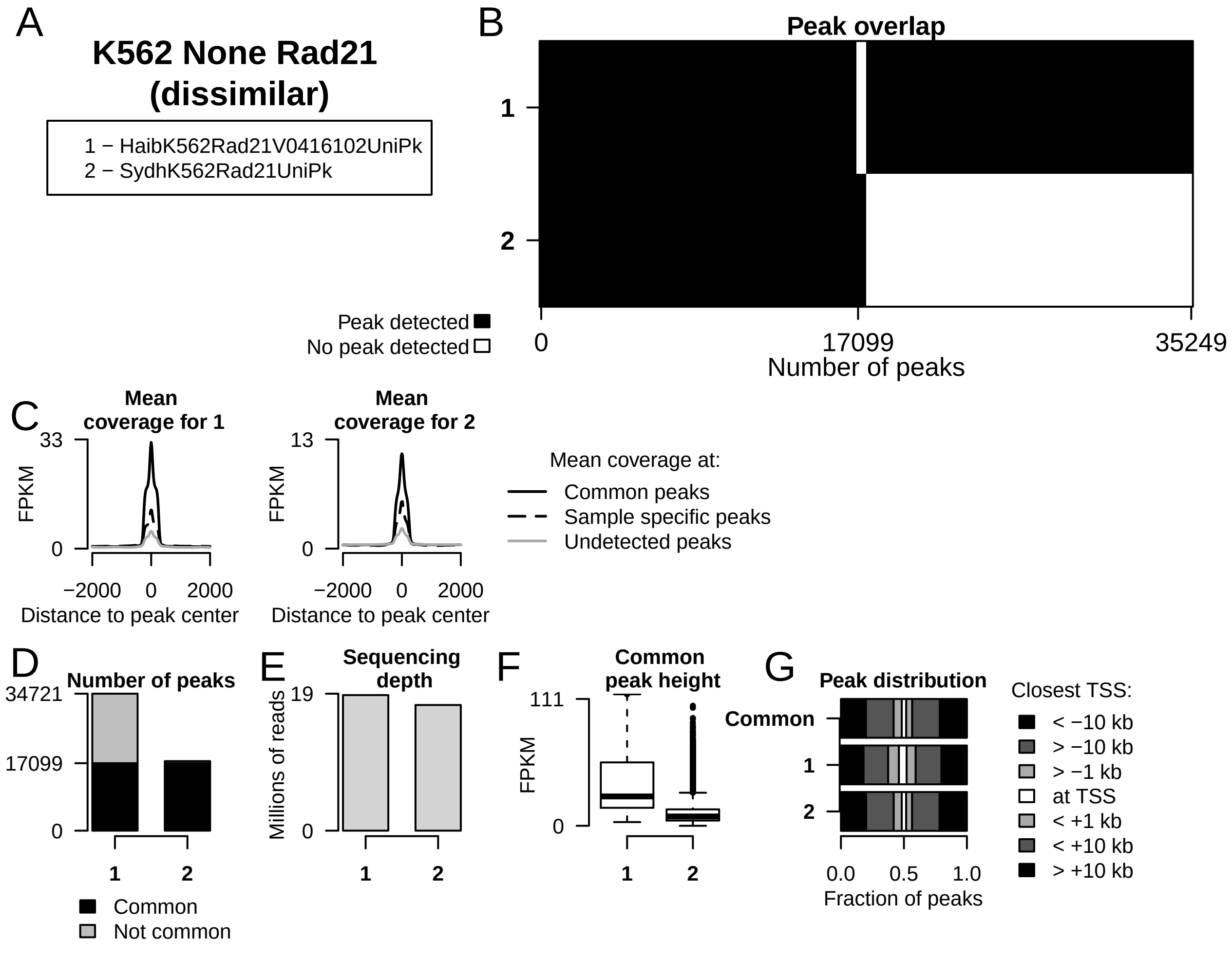

Supplementary figure 54: K562 None Rad21 (dissimilar)

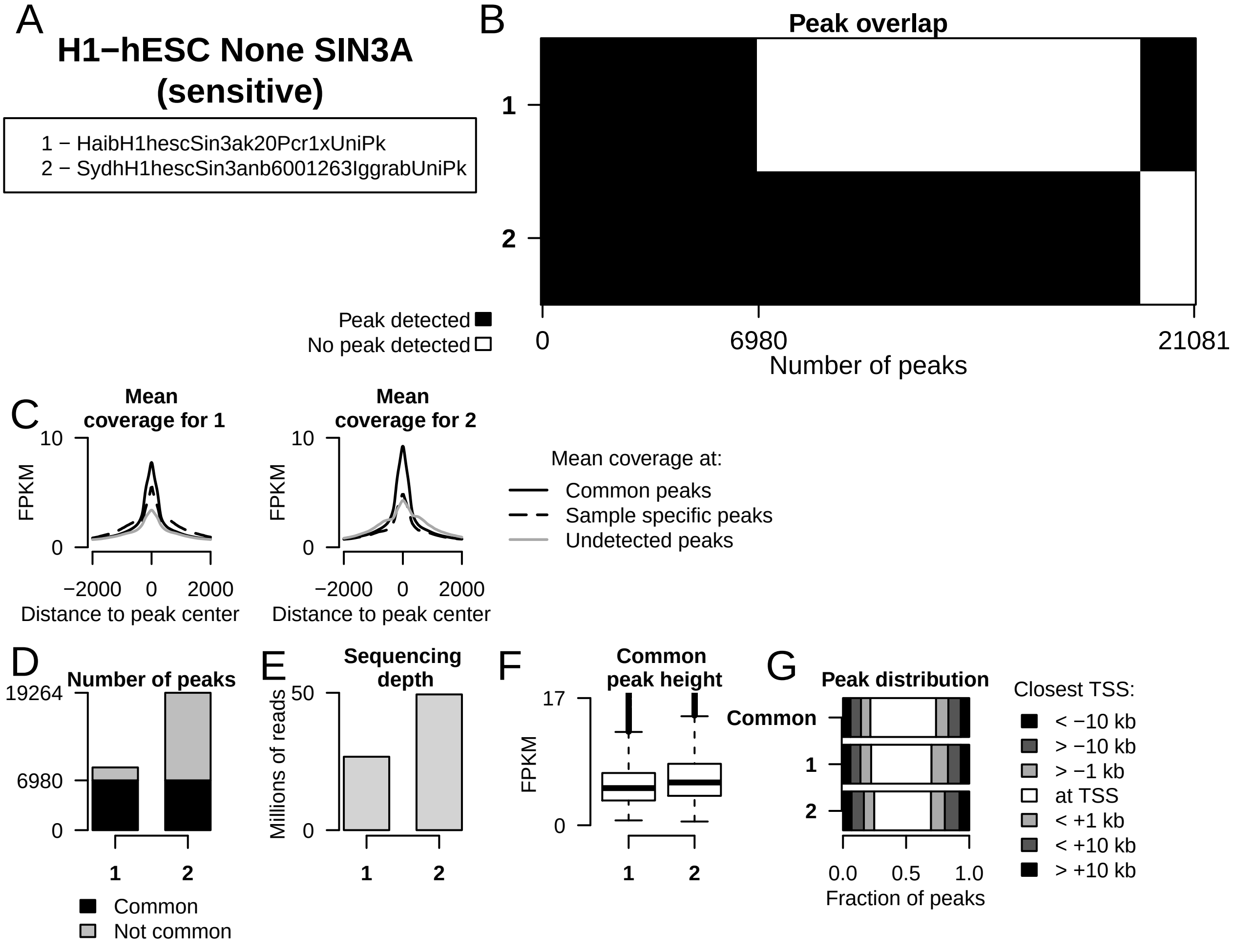

Supplementary figure 55: H1−hESC None SIN3A (sensitive)

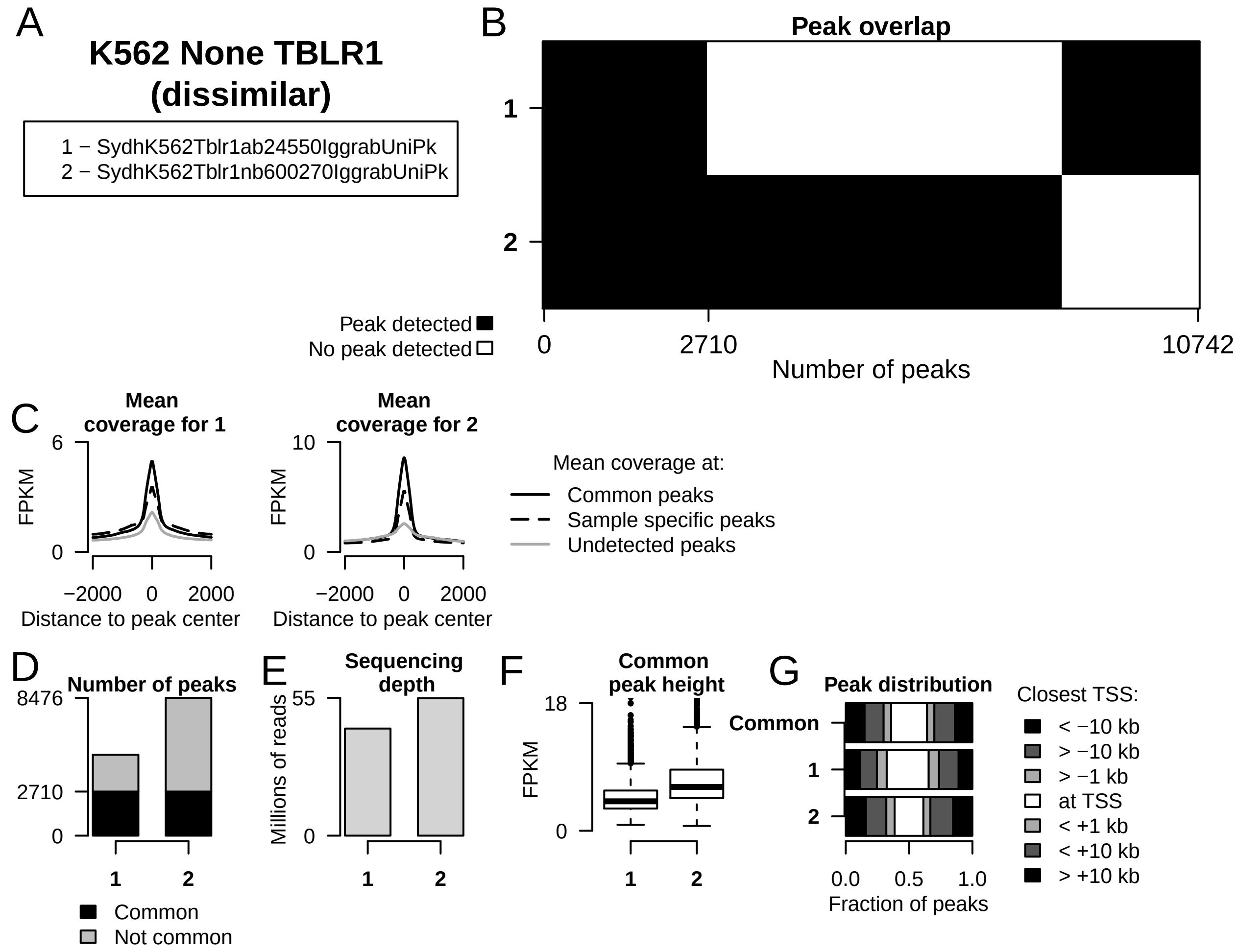

Supplementary figure 56: K562 None TBLR1 (dissimilar)

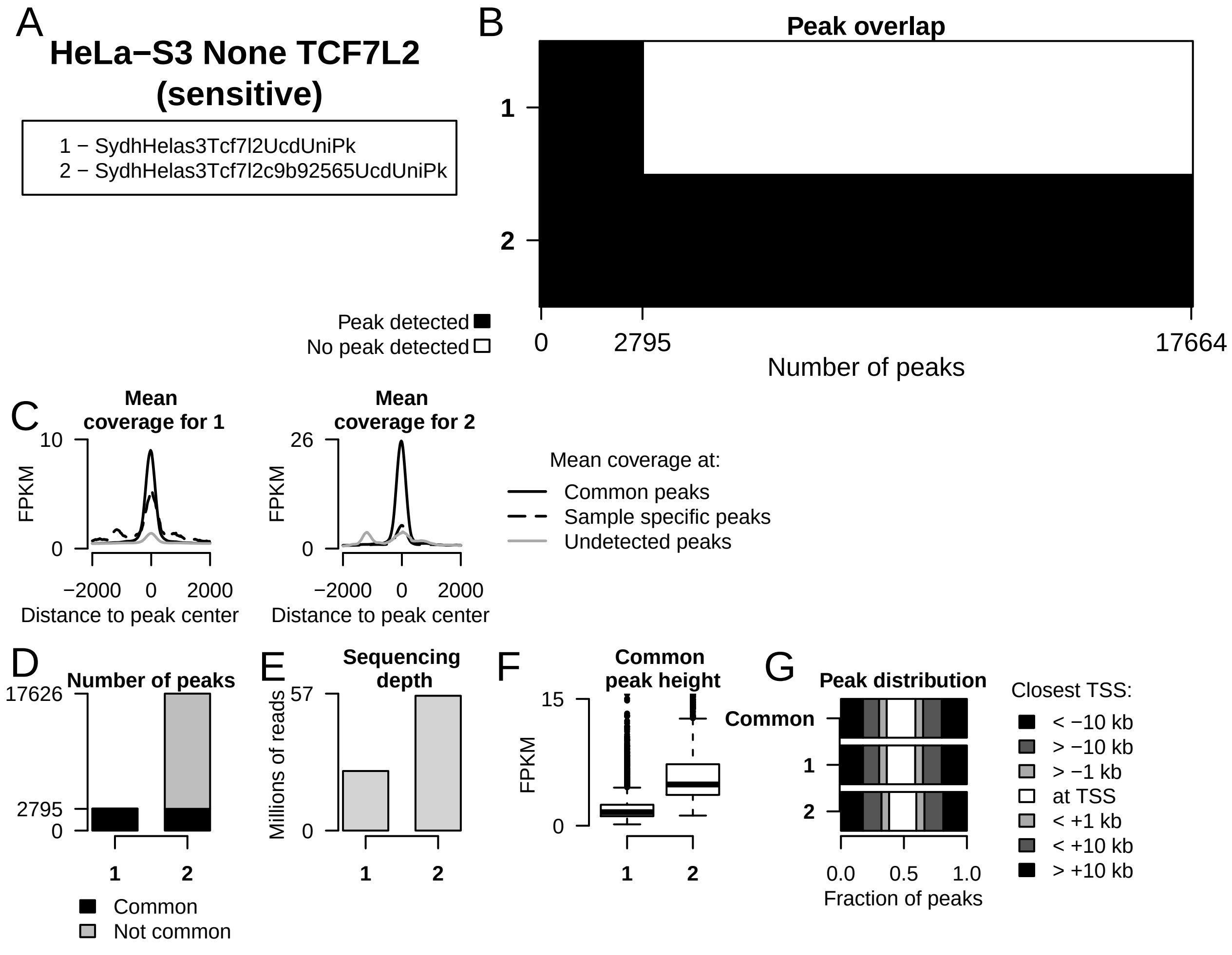

Supplementary figure 57: HeLa−S3 None TCF7L2 (sensitive)

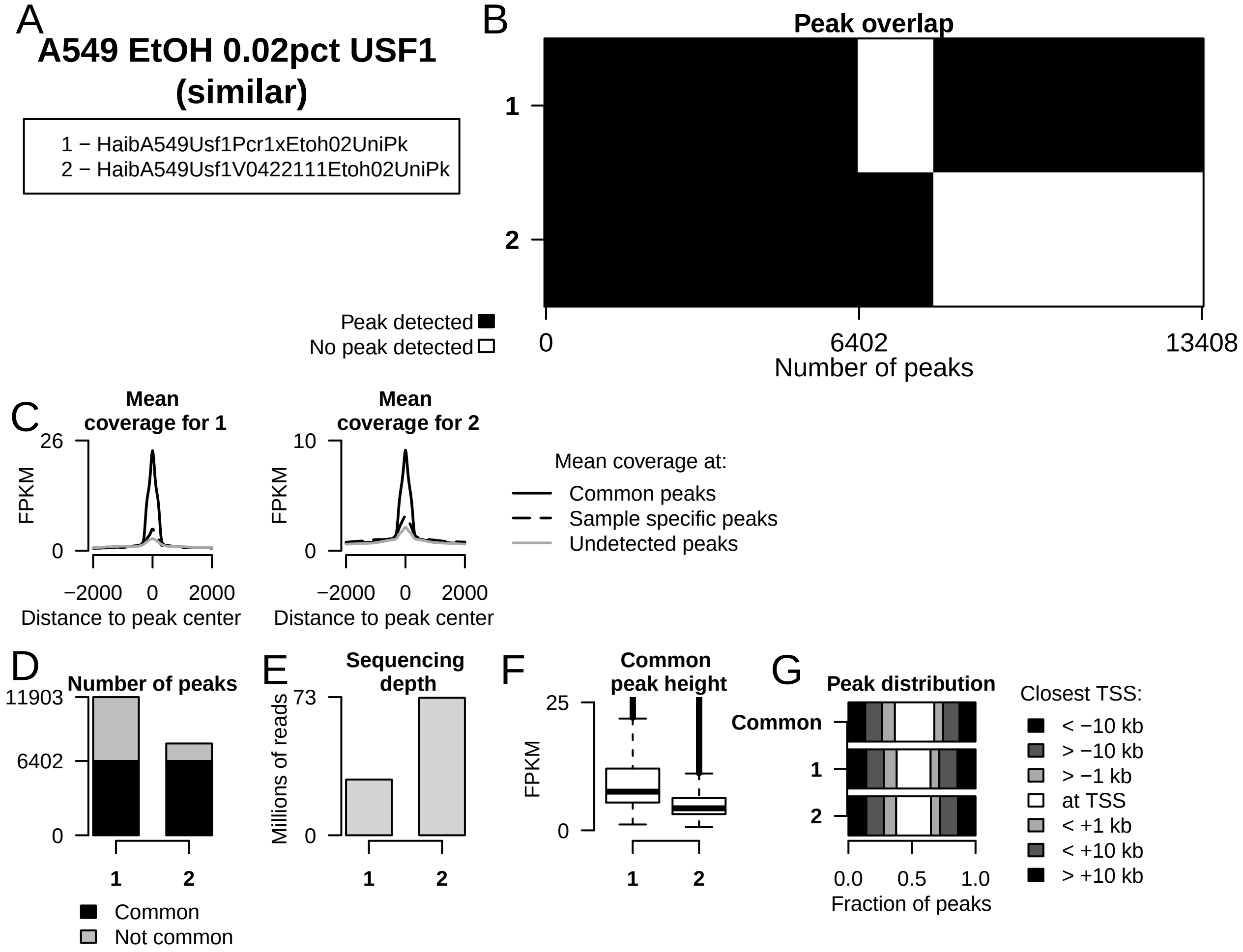

Supplementary figure 58: A549 EtOH 0.02pct USF1 (similar)

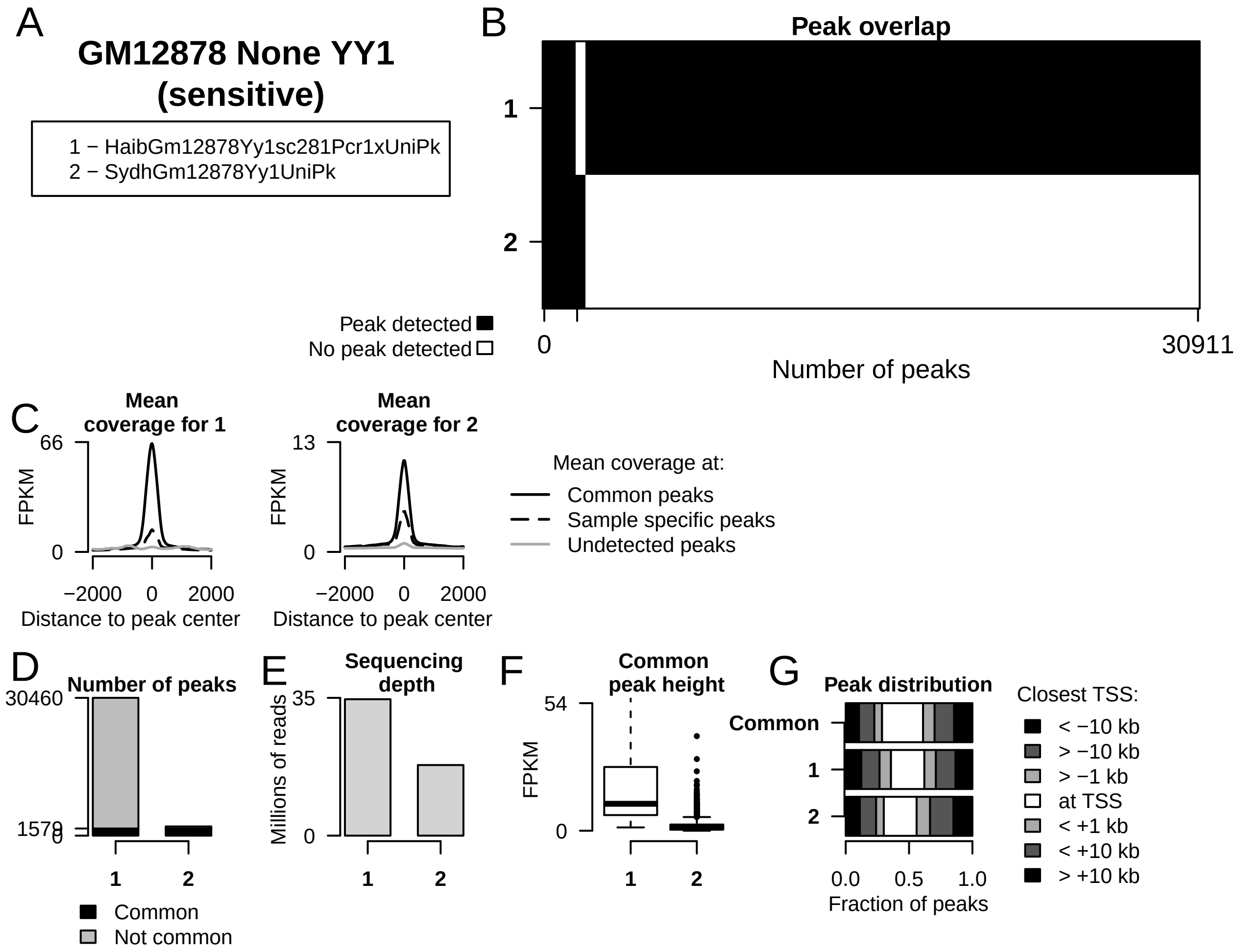

Supplementary figure 59: GM12878 None YY1 (sensitive)

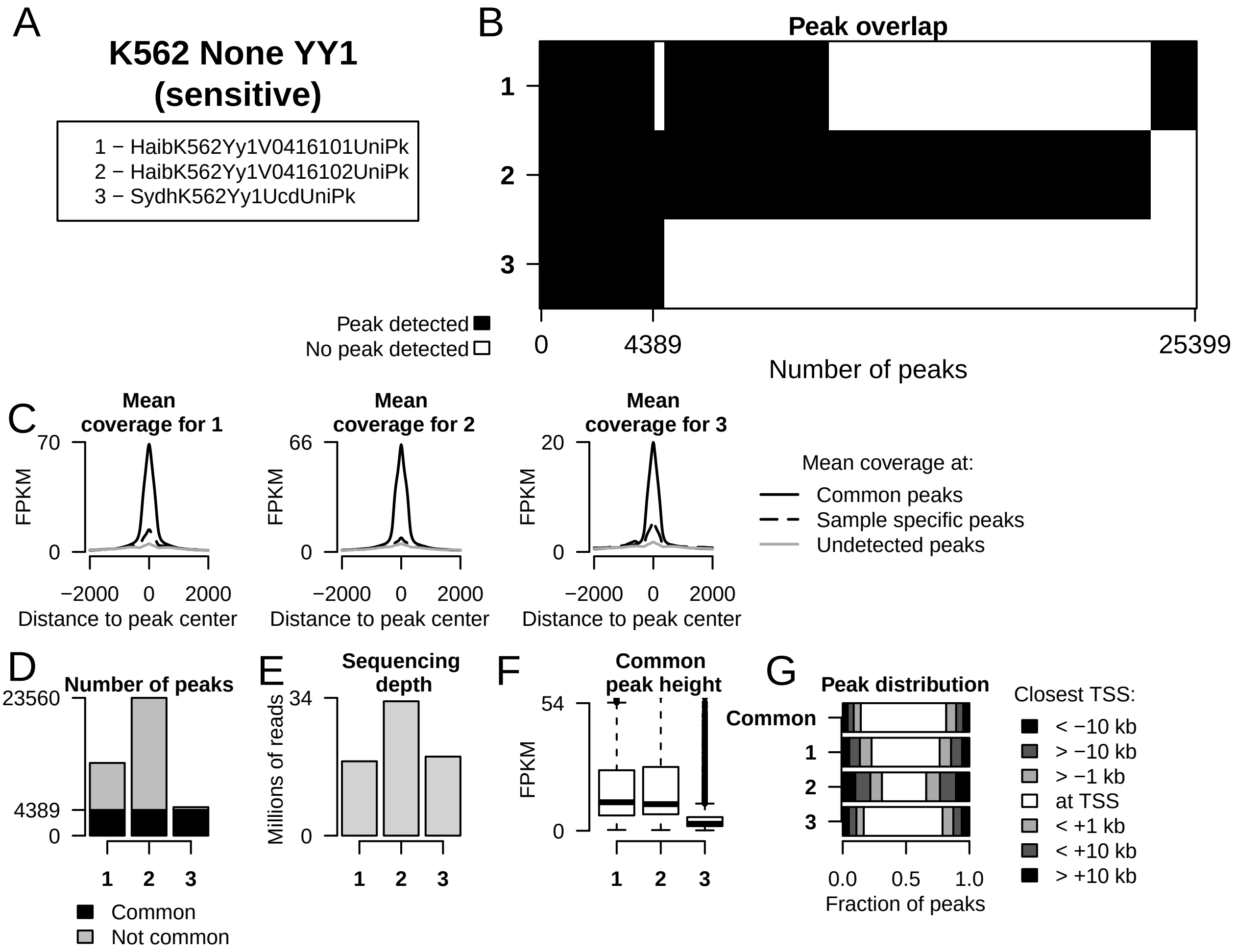

Supplementary figure 60: K562 None YY1 (sensitive)

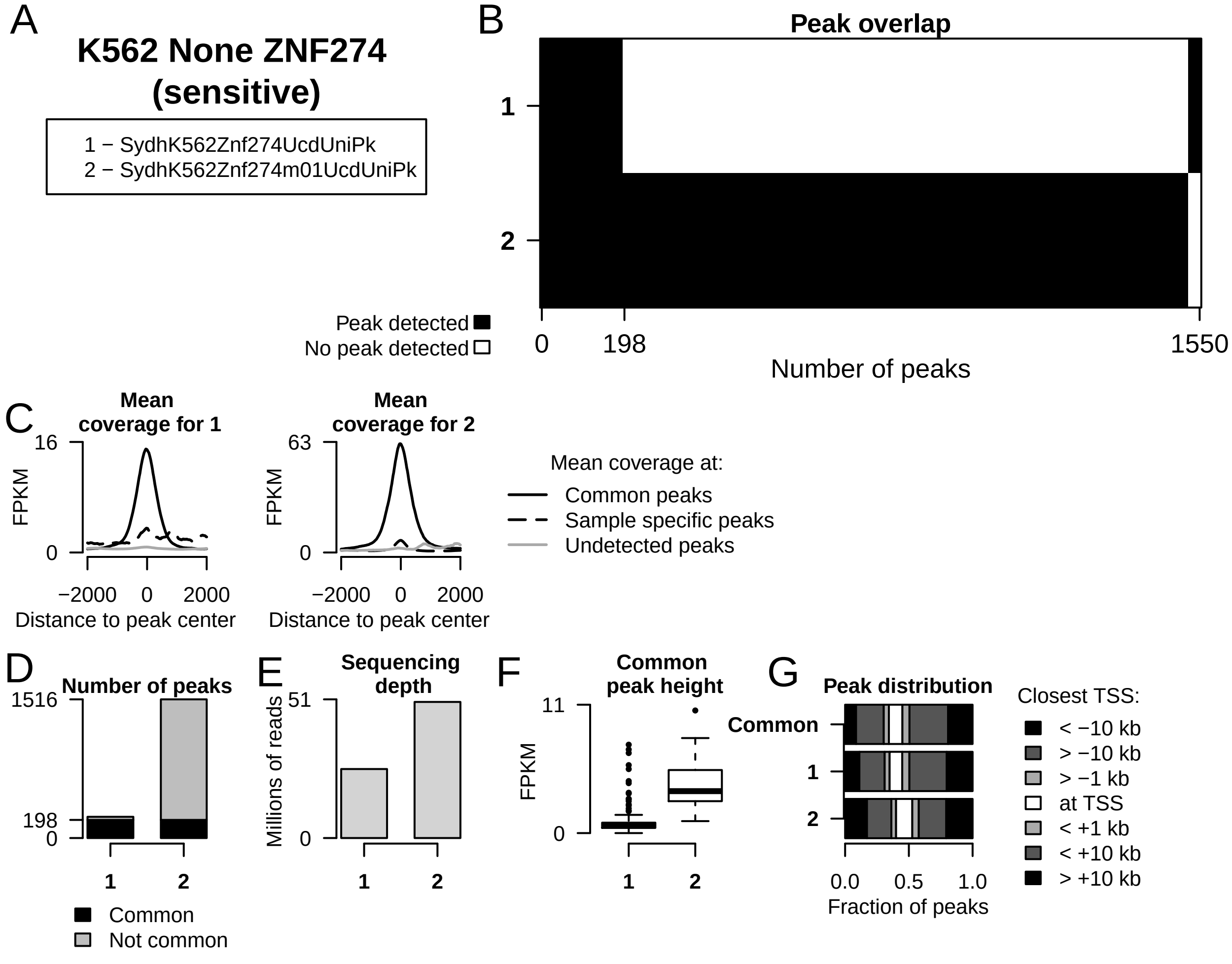

Supplementary figure 61: K562 None ZNF274 (sensitive)
